# Cytosine base editors optimized for genome editing in potato protoplasts

**DOI:** 10.3389/fgeed.2023.1247702

**Published:** 2023-08-30

**Authors:** Ida Westberg, Frida Meijer Carlsen, Ida Elisabeth Johansen, Bent Larsen Petersen

**Affiliations:** Department of Plant and Environmental Sciences, Faculty of Science, The University of Copenhagen, Frederiksberg, Denmark

**Keywords:** cytosine base editor, U6 promotor, native promotor, protoplast, potato, genome editing

## Abstract

In this study, we generated and compared three cytidine base editors (CBEs) tailor-made for potato (*Solanum tuberosum*), which conferred up to 43% C-to-T conversion of all alleles in the protoplast pool. Earlier, gene-edited potato plants were successfully generated by polyethylene glycol-mediated CRISPR/Cas9 transformation of protoplasts followed by explant regeneration. In one study, a 3–4-fold increase in editing efficiency was obtained by replacing the standard *Arabidopsis thaliana At*U6-1 promotor with endogenous potato *St*U6 promotors driving the expression of the gRNA. Here, we used this optimized construct (*Sp*Cas9/*St*U6-1::gRNA1, target gRNA sequence GGTC_4_C_5_TTGGAGC_12_AAAAC_17_TGG) for the generation of CBEs tailor-made for potato and tested for C-to-T base editing in the granule-bound starch synthase 1 gene in the cultivar Desiree. First, the *Streptococcus pyogenes* Cas9 was converted into a (D10A) nickase (nCas9). Next, one of three cytosine deaminases from human hAPOBEC3A (A3A), rat (evo_rAPOBEC1) (rA1), or sea lamprey (evo_*Pm*CDA1) (CDA1) was C-terminally fused to nCas9 and a uracil-DNA glycosylase inhibitor, with each module interspaced with flexible linkers. The CBEs were overall highly efficient, with A3A having the best overall base editing activity, with an average 34.5%, 34.5%, and 27% C-to-T conversion at C4, C5, and C12, respectively, whereas CDA1 showed an average base editing activity of 34.5%, 34%, and 14.25% C-to-T conversion at C4, C5, and C12, respectively. rA1 exhibited an average base editing activity of 18.75% and 19% at C4 and C5 and was the only base editor to show no C-to-T conversion at C12.

## Introduction

The CRISPR–Cas9 editing system/complex consists, in its basic form, of a guide RNA (gRNA) and a *Streptococcus pyogenes* nuclease *Sp*Cas9 enzyme, which generate a targeted double-stranded DNA break, leading to the formation of insertions and/or deletions (indels) via the activation of the non-homologous end joining (NHEJ) DNA repair pathway frequently resulting in frameshift of the reading frame and loss of gene function (LOF) (Jinek et al., 2012a). Basic CRISPR–*Sp*Cas9-mediated gene editing has been further developed into cytidine base editors (CBEs), where single targeted cytosines are converted into thymines (C-to-T) (Komor et al., 2016) and later expanded to include targeted adenine-to-guanine (A-to-G) adenine base editors (ABEs) (Gaudelli et al., 2017) and C-to-G base editors (Kurt et al., 2021). Base editing (BE) was first and mainly employed in mammalian systems (Komor et al., 2016; Nishida et al., 2016; Gaudelli et al., 2017; Komor et al., 2017; Koblan et al., 2018; Thuronyi et al., 2019; Koblan et al., 2021; Kurt et al., 2021) but have since been adjusted to plants, including crops such as rice (Shimatani et al., 2017; Zong et al., 2017; Zong et al., 2018; Jin et al., 2019; Li C. et al., 2020; Hua et al., 2020; Xiong et al., 2022), wheat (Zong et al., 2017; Zong et al., 2018), maize (Zong et al., 2017) potato (Zong et al., 2018; Veillet et al., 2019a; Veillet et al., 2019b; Veillet et al., 2020a; Veillet et al., 2020b), and tomato (Shimatani et al., 2017; Veillet et al., 2019b; Veillet et al., 2020b). BE has been introduced and tested in potato protoplasts using Agrobacterium-mediated delivery of integrative constructs followed by editing analysis of regenerated explants (Zong et al., 2018; Veillet et al., 2019a; Veillet et al., 2019b; Veillet et al., 2020a; Veillet et al., 2020b) and using PEG-mediated delivery of non-integrative constructs into potato protoplasts (Zong et al., 2018). Both approaches included targeting Granular-bound starch synthase (*St*GBSS), where Agrobacterium-mediated delivery generally conferred high C-to-T conversion, some indel formation, and undesired C-to-A and C-to-G conversions in the explants examined (Veillet et al. (2019a), and delivery to protoplasts, in one instance, conferred an average of up to 18%–20% of C-to-T editing (Zong et al., 2018). Prime editing (PE) is a recent additional editing tool that allows controlled editing directly into the target site through the use of a reverse transcriptase and a specialized prime editing guide RNA (pegRNA), which confers the targeting and editing specificity and the binding capability to the nickase (nCas9) of the prime editing complex (Anzalone et al., 2019). PE has interesting potential within clinical applications (Surun et al., 2020; Geurts et al., 2021; Happi Mbakam et al., 2022a; Happi Mbakam et al., 2022b; Tremblay et al., 2022) and in crop breeding (Jiang et al., 2020b; Li H. Y. et al., 2020; Lin et al., 2020). Implementation of PE in plants on a wider scale, however, has proven difficult, perhaps due to the mode of action and the complex pegRNA structure (Zhao et al., 2023), underpinning the continued relevance of base editing. However, the applicability of base editing on a wider scale is constrained by moderate targeting specificity and efficiencies, which, to some degree, may be alleviated by design and efficiency optimizations of the BE construct at hand. Here, we further developed a non-integrative CRISPR/*Sp*Cas9 construct, optimized and custom-made for potato protoplasts via replacement of the standard *At*U6-1 promotor with a native potato *St*U6-1 promotor, to generate and compare three CBE constructs with different origins of the deaminase. When targeted to the granule-bound starch synthase (GBSS) 1 gene and tested on protoplasts of the cultivar Desiree, the three BEs generally conferred high C-to-T base editing efficiencies with, in one instance, 43% C-to-T conversion of a single cytosine.

## Materials and methods

### Strains and cultivars

Potato (*Solanum tuberosum*) cultivar Desiree plantlets were grown and maintained *in vitro* on medium A, as described in the work of Nicolia et al. (2015) and Nicolia et al. (2021). The potato plants were grown in a Fitotron growth cabinet model SGC 120 from Weiss Technik with a diurnal rhythm of 16/8 h, 24°C/20°C, 70% humidity, at a light intensity of 65 μE.

### Base editor construct assembly

The basic construct, *Sp*Cas9/*St*U6-1::sgRNA1, comprising the 35SPPDK::*Sp*Cas9 cassette, driving the expression of the codon-optimized *Streptococcus pyogenes* Cas9 nuclease (*Sp*Cas9) originally from the plasmid pHBT-pcoCas9 (Li et al., 2015) (Addgene plasmid #52254) and the *St*U6-1 promoter::sgRNA-1 cassette (*St*U6-1 promoter (NCBI accession no. Z17290)) described in the work of Johansen et al. (2019) was used as the basis for generation of the base editing constructs. Each of the cytosine deaminases, hAPOBEC3A (A3A) (Zong et al., 2018) (Addgene #119768) from human, evo_rAPOBEC1 (rA1) (Thuronyi et al., 2019) (Addgene #122611) from rat, and evo_*Pm*CDA1 (CDA1) (Thuronyi et al., 2019) (Addgene #122608) from sea lamprey (*Petromyzon marinus*), were codon-optimized for potato and purchased from GenScript (https://www.genscript.com), delivered in the pUC57 vector. The plasmid *Sp*Cas9/*St*U6-1::sgRNA1 cassette was used first for the generation of the nCas9 nickase (D10A) using site-directed mutagenesis, which then served as the basis for the generation of the base editing constructs via Gibson construct assembly with either of the cytosine deaminases using the NEBuilder HiFi DNA Assembly Master Mix (New England BioLabs) according to the manufacturer’s recommendations. Final constructs consisted of the 35SPPDK::nCas9 cassette, the *St*U6-1 promoter::sgRNA1, and one of three deaminases A3A, rA1, and CDA1, a uracil glycosylase inhibitor (UGI), interconnected by flexible serine–glycine (SG)-extended XTEN linker (SGGSSGGSSGSETPGTSESATPESSGGSSGGS) and serine–glycine–glycine–serine (SGGS) linkers.

Codon-optimized nucleotide sequences including assembly overhangs are provided in Supplementary Information.

### Site-directed mutagenesis

The D10A mutation was introduced into *Sp*Cas9/*St*U6-1::sgRNA1 by site-directed mutagenesis PCR using 12.5 pmol of primer 567 and 12.5 pmol of primer 568, 12.5 μL 2 X CloneAmp^TM^HiFi PCR Premix from Takara, and 100 ng of template (*Sp*Cas9/*St*U6-1::sgRNA1) in a total reaction volume of 25 μL. PCR cycle parameters were 98°C 3 min, followed by 15 cycles of 98°C for 10 s, 55°C for 15 s, and 72°C for 5 min.

### Oligonucleotide primers

Primers were ordered from TAG Copenhagen A/S (https://www.tagc.com) and are listed in Supplementary Table S1. For working applications, 5 pmol/μL dilutions in Milli-Q water were prepared.

### Gibson assembly mix transformation and sequence verification

2 μL of Gibson assembly mix (Base editor construct assembly) was transformed into and multiplied in *E. coli*. Plasmids were extracted using the E.Z.N.A.(R) Plasmid DNA Mini Kit I (D6943-02) from Omega Bio-tek according to the manufacturer’s instructions and sequenced by EZ-sequencing services provided by Macrogen to ascertain the correct sequence.

### Large-scale plasmid editor purification and preparation for transformation

Following confirmation of the correct sequence, plasmids were amplified in *E. coli* and isolated by CTAB large-scale-prep plasmid phenol extraction and then diluted to a concentration of 1 μg/μL to be used for protoplast transformation.

### Protoplast isolation and transformation

Media used for isolation and transformation include medium B, plasmolysis solution, medium C, wash solution, sucrose solution, transformation buffer 1, transformation buffer 2, PEG solution, and medium E, with recipes outlined in the work of Nicolia et al. (2021). Protoplast isolation was carried out as described in the work of Nicolia et al. (2015) and Nicolia et al. (2021). The intactness and purity of isolated protoplasts were checked by light microscopy and diluted to a concentration of ca. 1.6 × 10^3^ protoplasts/μL in transformation buffer 2. Then, 110 µL protoplasts (ca. 1.6 × 10^3^ protoplasts/μL) in transformation buffer 2 were gently mixed with 10 µL (1 μg/μL) of base editing plasmid, and 110 µL 25% PEG solution was added, gently mixed, and incubated for 3 min at RT. Transfection was stopped by adding 6 mL of wash solution and then spun at 500 RPM for 5 min (minimum acceleration and deceleration), RT, the wash solution was carefully removed, and 1 mL of ½ medium E (diluted with 0.4 M sorbitol) was added. Protoplasts were then incubated in the dark for 2 days at 60 RPM, RT, which yielded optimal editing when using the original construct (Johansen et al., 2019). Following incubation, the protoplasts were harvested by spinning for 3 min at 4000 RPM, and the pellet was re-dissolved in 50 µL of Milli-Q water, frozen in N_2_, heated for 15 min at 96°C, and stored at −20°C. The protoplast slurry was thawed, placed on ice, and then, vortexed prior to entering as a template in PCR amplifications.

### PCR amplification and product purification

PCR amplification of the target region of GBSS1 was performed using 6.25 pmol of primer 472 and 6.25 pmol of primer 384, 12.5 µL 2 X CloneAmp^TM^HiFi PCR Premix from Takara, and 1 µL of protoplast slurry (ca. 1.6 × 10^3^ protoplasts/μL) in a total volume of 25 µL. PCR cycle parameters were 2 min at 98°C, followed by 40 cycles of 10 s at 98°C, 15 s at 64°C, and 30 s at 72°C, followed by 2 min at 72°C. PCR products were purified using the BioLine ISOLATE II PCR & Gel Kit or NucleoSpin Gel and the PCR Clean-up Mini kit from Macherey-Nagel according to the manufacturer’s recommendations. PCR products were then sent for sequencing (Sequencing directly on PCR products).

### Indel detection amplicon analysis

PCR amplification of the GBSS1 target region was performed using 6.25 pmol of primer 475 and 6.25 pmol of primer FAM481 (5’ end labeled with fluorescein amidite (FAM)), 12.5 µL 2 X CloneAmp^TM^HiFi PCR Premix from Takara, and 1 µL of protoplast slurry in a total volume of 25 µL. PCR cycle parameters were 2 min at 98°C, followed by 40 cycles of 10 s at 98°C, 15 s at 64°C, and 30 s at 72°C, followed by 2 min at 72°C. PCR amplicons were wrapped in aluminum foil and stored at −20°C until being subjected to indel detection amplicon analysis (IDAA) analysis at COBO Technologies Aps, Denmark, where the fluorescently labeled fragments were run on a sequenator 3500xL Genetic Analyzer (Applied Biosystems) and separated according to size by capillary electrophoresis, with a separation resolution down to fragments differing ±1 bp in length as described in the work of Yang et al. (2015).

### Restriction digestion

StyI digestions were performed in a total volume of 10 μL containing 80 ng of PCR fragment DNA, 1 μL 10 x CutSmart Buffer (New England BioLabs), and 2 U StyI enzyme (New England BioLabs) and incubated at 37°C for 3 h. Then, 4 U of StyI enzyme was additionally added and incubated for 1 h. BsrI digestion was performed in a total volume of 10 μL containing 80 ng of PCR fragment DNA, 1 μL 10 x NEBuffer 3.1 (New England BioLabs), and 2 U BsrI enzyme (New England BioLabs) and incubated at 65°C for 2 h.

### Sequencing directly on PCR products

Editing was also analyzed by Sanger sequencing, using the EZ-seq sequencing services provided by Macrogen, directly on PCR products using 20 ng of purified PCR product (PCR amplification and product purification) and 25 pmol of primer 589. It should be noted that for direct sequencing on PCR amplicons of the protoplast cell pool, discernable/readable sequence chromatograms were only obtained when using ca. 20 ng of purified PCR product as opposed to the 50–75 ng recommended by Macrogen EZ-seq.

### Data analysis

Editing efficiency was determined by analyzing sequence chromatograms using the EditR software (Kluesner et al., 2018). IDAA chromatograms were obtained using the online software VIKING (https://viking-suite.com/).

## Results

**FIGURE 1:**
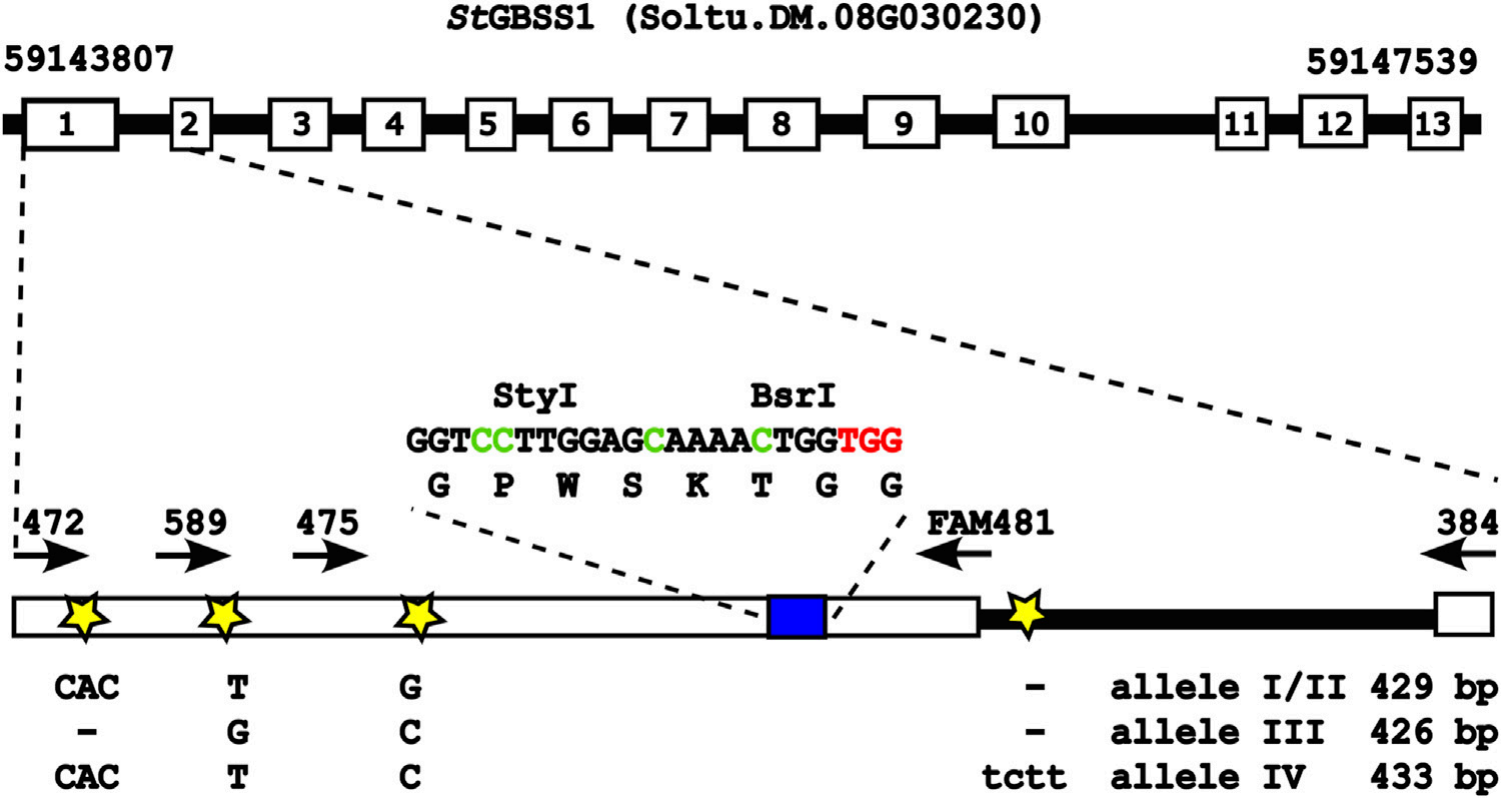
GBSS 1 target gene (cultivar Desiree). Exon 1 of the GBSS 1 target gene with both length and SNPs between the four alleles in the cultivar Desiree is indicated. The target gRNA sequence GGT​C_4_C_5_TTG​GAG​C_12_AAA​AC_17_TGG (blue box), with target cytosines (Cs) in green, PAM in red, and the diagnostic restrictions sites StyI and BsrI, is indicated. White numbered boxes depict exons, while stars indicate SNPs or size polymorphisms between the four alleles. Arrows indicate diagnostic PCR primers for editing scoring, amplifying the target region inside or outside the length polymorphisms. The figure is based on and adapted from the work of Johansen et al. (2019).

Earlier, we used CRISPR/Cas9 for knockout of the GBSS 1 target gene in potato (*Solanum tuberosum*) (cultivar Desiree and Wotan), where the CRISPR/Cas9 components were transiently expressed from plasmids delivered by polyethylene glycol (PEG)-mediated transformation to protoplasts and explants regenerated from single edited protoplast cells (Johansen et al., 2019). Here, the target region of *St*GBSS1 (5’ UTR, exon 1, intron 1, including length and single-nucleotide polymorphisms (SNPs)) in the potato cultivars Desiree and Wotan were sequenced and mapped, providing the allele-specific foundation for gRNA and diagnostic PCR primer designs for targeting and editing scoring of the CBE editors A3A, rA1, and CDA1 (Figure 1) in the present study.

**FIGURE 2:**
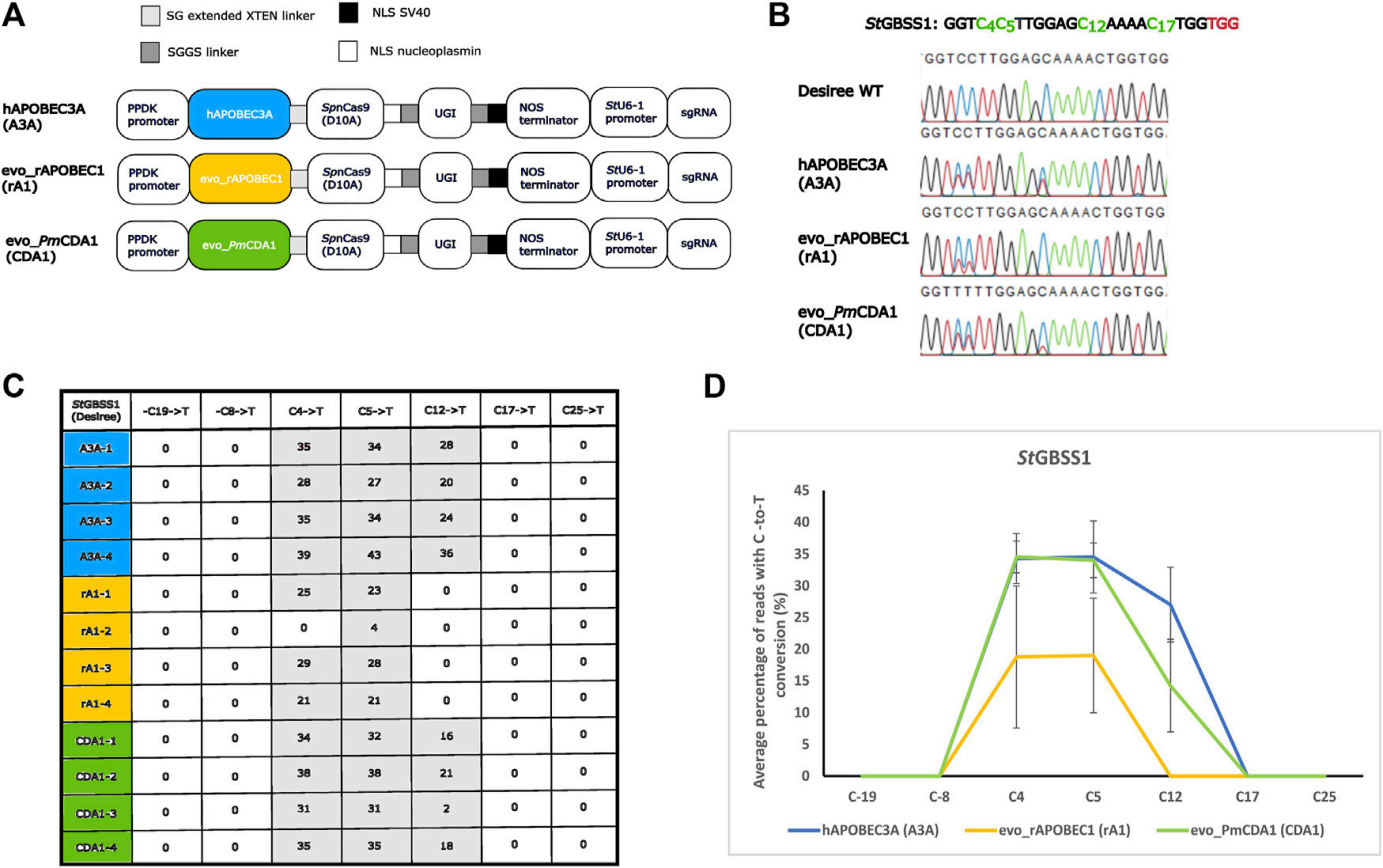
C-to-T conversion derived from cytosine base editors A3A, rA1, and CDA1 optimized for potato. **(A)** Three cytosine base editor constructs comprising a cassette driving expression of the gRNA (target conferring part, GGT​C_4_C_5_TTG​GAG​C_12_AAA​AC_17_TGG) from the *St*U6-1 promotor (Johansen et al., 2019), the *Sp*Cas9 nickase (nCas9) in fusion with one of the deaminases hAPOBEC3A (A3A) from human, evo_rAPOBEC1 (rA1) from rat, or sea lamprey evo_*Pm*CDA1 (CDA1), followed by a uracil-DNA glycosylase inhibitor (UGI), interspaced by long flexible linkers are depicted. NLS, nuclear localization signal; serine (S) and glycine (G) linkers: SG and SGGS. **(B)** Chromatograms from direct sequencing of PCR products from the protoplast cell pool transformed with the base editing constructs with A3A showing 39% (C4), 43% (C5), and 36% (C12) editing (sample A3A replicate 4), rA1 showing 29% (C4) and 28% (C5) (sample rA1 replicate 3), and CDA1 showing 38% (C4), 38% (C5), and 21% (C12) (sample CDA1 replicate 2) C-to-T conversion in the target region (exon 1 of GBSS1 (cultivar Desiree)) when compared to the WT sequence. Target Cs in the gRNA and adjacent PAM site are shown in green and red, respectively. **(C)** C-to-T conversion (%) of Cs within (C4, C5, C12, and C17) and most closely adjacent (−C19, −C8 and C25) to the gRNA of protoplasts transformed with A3A, rA1, and CDA1 as evidenced by EditR analysis. Numbers 1–4 indicate replicates. **(D)** Average percentage of reads with C-to-T conversion (%) rates of protoplasts transformed with A3A, rA1, and CDA1 as shown for Cs within (C4, C5, C12, and C17) and most closely adjacent (−C19, −C8 and C25) to the gRNA. Data are shown as mean ± sd of four biological replicates.

Nickase and cytidine base editing activities were tested by the transient expression of the SpCas9/StU6-1::sgRNA1 nickase construct or the C-to-T base editors A3A, rA1, and CDA1 using PEG transformation of isolated potato protoplasts (cell pool) of cultivar Desiree, which were then cultured for 2 days as described in the work of Nicolia et al. (2015) and Nicolia et al. (2021) and outlined in Materials and methods, after which the target region was PCR-amplified, and each PCR amplicon was analyzed by both IDAA and amplicon sequencing, including EditR analysis, for potential nuclease-induced indels and C-to-T base editing activity. First, the *Sp*Cas9 in the construct *Sp*Cas9/*St*U6-1::sgRNA1 (Johansen et al., 2019) was converted into a nickase (nCas9) by changing the aspartic acid (Asp10) into alanine (Ala10) (D10A) (Jinek et al., 2012b) through the use of site-directed mutagenesis. The absence of nuclease activity from nCas9 was confirmed by full digestion of the BsrI restriction site situated 3 bp upstream of the protospacer adjacent motif (PAM) and confirmed by IDAA (Yang et al., 2015; Bennett et al., 2020), which displayed a PCR amplicon with unchanged length (see Supplementary Information) and sanger sequencing of PCR products (data not shown). Effect of placement of the three deaminases, A3A (human hAPOBEC3A), rA1 (rat evo_rAPOBEC1), and CDA1 (sea lamprey *Petromyzon marinus*, evo_*Pm*CDA1), and the use of different linkers between fusion partners have been investigated earlier (Nishida et al., 2016; Zong et al., 2018; Tan et al., 2019; Thuronyi et al., 2019; Choi et al., 2021; Huang et al., 2021). In the present study, the deaminase was fused to the N-terminal of nCas9 because the three deaminases have been proven to be functionally active in this design and in order to enable comparison between the three CBEs. The CBEs, A3A, rA1, and CDA1, were initially scored for editing activity by checking for destruction of the StyI restriction site (C_4_C_5_WWGG) 11 bp upstream of the PAM site (TGG), where conversion of either or both of the two cytosines C4 and C5 would lead to resistance to StyI digestion (see Supplementary Information). C-to-T editing efficiencies of A3A, rA1, and CDA1 were confirmed and scored by direct sequencing and quantified using the EditR software (Kluesner et al., 2018), with A3A having the best overall activity with an average 34.5%, 34.5%, and 27% C-to-T conversion at C4, C5, and C12 in the target (gRNA) sequence (GGT​C_4_C_5_TTG​GAG​C_12_AAA​AC_17_TGG), respectively, whereas CDA1 showed an average C-to-T conversion of 34.5%, 34%, and 14.25% at C4, C5, and C12, respectively. rA1 showed an average C-to-T conversion of 18.75% and 19% at C4 and C5 and was the only base editor to show no C-to-T conversion at C12. C17 conversion was not observed for any of the three base editors. All three base editors showed stable conversion rates with at least 21% C-to-T conversion for C4 and C5 (a single exception being rA1 replicate 2), and A3A and CDA1 showed an average 34% conversion rate for C4 and C5. The highest C-to-T conversion was observed for A3A replicate 4, which showed 39%, 43%, and 36% for C4, C5, and C12, respectively (Figures 2B–D). Neither indel formation, as evidenced by IDAA and direct sequencing results (Supplementary Information), nor unintended C-to-A or C-to-G changes, as evidenced by direct sequencing results (Figure 2B and Supplementary Information) and EditR analysis (Figure 2C and Supplementary Information), were encountered in the present study, which, however, was confined to the protoplast pool. Direct sequencing identified two allele-specific SNPs (Figure 1 and Supplementary Information), indicating amplification of the four alleles. Detailed information regarding constructs, protoplast isolation, PEG-mediated transformation and incubation, restriction enzyme, IDAA analyses, and direct sequencing on the protoplast cell pool is provided in Materials and methods and Supplementary Information.

## Discussion

The use of CRISPR-based precise gene editing, including base and prime editing, for crop improvement has recently been reviewed (Butt et al., 2020; Gurel et al., 2020; Mishra et al., 2020), with a particular focus on potato protoplasts, e.g., provided in the work of Hofvander et al. (2022).

Here, we further developed a CRISPR/*Sp*Cas9 construct optimized for potato, where replacement of the standard *Arabidopsis thaliana At*U6-1 promotor driving the expression of the gRNA, with the endogenous potato *St*U6-1 promotor, resulted in a 3–4-fold increase in editing efficiencies at the protoplast cell pool level (Johansen et al., 2019), into CBE constructs. We used three different CBE constructs, in which either of three deaminases, A3A (human hAPOBEC3A), rA1 (rat evo_rAPOBEC1), and CDA1 (sea lamprey *Petromyzon marinus*, evo_*Pm*CDA1), were C-terminally fused to a *Sp*Cas9 nickase (nCas9) and uracil-DNA glycosylase inhibitor (UGI), which were combined with the native potato *St*U6-1::gRNA-1 cassette expressing the gRNA. Each CBE was targeted to exon 1 of the GBSS1 gene and transformed into protoplasts of potato cultivar Desiree with their base editing conversions scored. All three constructs displayed high C-to-T conversion activities, peaking at C4 and C5 in the target (GGT​C_4_C_5_TTG​GAG​C_12_AAA​AC_17_TGG​TGG) sequence, with A3A, CDA1, and rA1 displaying average C4 and C5 C-to-T conversions of 34.5% & 34.5%, 34.5% & 34%, and 18.75% & 19%, respectively. A3A and CDA1 displayed 27% and 14.25% C-to-T conversion at C12, and rA1 showed no C12 C-to-T conversion, which is in agreement with the fact that the rAPOBEC1 deaminase, from which rA1 is derived, has previously been reported to be inefficient in a GC context (Zong et al., 2018). The importance of the sequential location of Cs and different sequence preferences for different deaminases have been highlighted in other studies (Tan et al., 2019; Tan et al., 2020; Huang et al., 2021).

With the exception of a single replicate, all three CBEs conferred >= 21% C-to-T conversion of C4 and C5 in the target sequence (see Figure 2C rA1-2), with an average 34% C-to-T conversion for C4 and C5 for both the A3A and CDA1, which, to our knowledge, are the highest average C-to-T conversions obtained when employing PEG-mediated delivery into potato protoplasts. In comparison, Zong et al. (2018) obtained, in one instance, an average of up to 18%–20% C-to-T editing when targeting the *St*GBSS1 gene in potato protoplasts (Zong et al., 2018). Protoplasts transformed with non-integrative constructs will, unlike agrobacterium-transformed plants that may display chimerism (Faize et al., 2010), generate single-protoplast-cell-derived genetically uniform explants and enable a potential replacement of plasmid with ribonucleoprotein (RNP), thereby excluding the presence of DNA in the entire editing process.

The averaged significantly higher editing efficiency obtained in the present study may be attributed to the use of the native potato *St*U6-1 promoter, driving the gRNA, which appeared rate limiting in Johansen et al.’s (2019) study, although differences in CBE construct architecture and composition or methodology may also be contributing factors. Zong et al., 2018 pioneered the implementation of C-to-T base editing in plants using the human APOBEC3A-based and the rat APOBEC1-based cytidine deaminase construct (Zong et al., 2018). The APOBEC3A- and APOBEC1-based CBEs were delivered into cells of potato, rice, and wheat by PEG-mediated transformation of protoplasts, Agrobacterium-mediated transformation of callus, or biolistic delivery into immature embryo cells. In most experiments, the human APOBEC3A-based CBE outperformed the rat APOBEC1-based CBE, a tendency which was confirmed for the A3A and rA1 CBEs generated and tested in the present study.

Distribution of C-to-T conversion across the target sequence, i.e., editing frequencies at C4, C5, C12 and C17, seemed to be somewhat similar to what has been reported for other base editing constructs (Huang et al., 2021). However, careful construct design/architecture, e.g., adjustments of flexible linker lengths, may elevate a desired target position accuracy (Tan et al., 2019; Tan et al., 2020). In addition, the development of base editors with alternative PAM specificities expands the freedom to operate and may potentially affect precision (Veillet et al., 2020a; Veillet et al., 2020b). The base editing efficiencies presented here were obtained via transient non-integrative PEG transformation of the protoplast cell pool level, where the A3A CBE, in one instance, conferred 43% C-to-T conversion of C5.

CBEs have, in some settings, been reported to additionally generate C-to-G or C-to-A conversions, although at lower frequencies than the targeted C-to-T conversions (Komor et al., 2017) and indels, whereas in one study, 75% of explants transformed with an agrobacterium-mediated integrative CBE construct were found to contain indels (Veillet et al., 2019b). Similar undesired conversions or indel formation, e.g., as evidenced by direct sequencing, EditR analysis, and IDAA, were, within the resolution of the analytic methods applied, not encountered in the present study, which, however, was confined to the protoplast cell pool.

PE enables controlled generation of small insertions, deletions, or base substitutions as part of the prime editing guide RNA (pegRNA) and was originally described as a tool for correcting DNA in humans in relation to disease (Anzalone et al., 2019). PE has also been applied in plants, such as rice (Li H. Y. et al., 2020; Lin et al., 2020) and maize (Jiang et al., 2020a), with moderate success, highlighting the importance of testing a range of pegRNAs. Recent implementation of PE in the model plant *Physcomitrium patens* and tetraploid potato also pinpointed limitations of the technology, which need to be overcome before PE may become a versatile efficient tool in precision plant breeding (Perroud et al., 2022). The PE repertoire has, as in the case of the base editor repertoire, been expanded with alternative PAM specificities (Kweon et al., 2021).

The construct design and protocols for scoring C-to-T base editing presented in this study may readily be converted into A-to-G base editors (ABEs), probably with comparable efficiencies. Thus, for now, and with the editing efficacies obtained in this study, BE still remains a competitive relevant tool in the toolbox for precise plant breeding.

## Data Availability

The original contributions presented in the study are included in the article/Supplementary Material; further inquiries can be directed to the corresponding author.
