# Supplementary material for: Cytosine base editors optimized for genome editing in potato protoplasts
Source: Front Genome Ed. 2023 Aug 30;5:1247702. doi: 10.3389/fgeed.2023.1247702 (PMC10502308; doi:10.3389/fgeed.2023.1247702)

**Supplementary Information**

**Oligonucleotide primers**

Table 1.

| Name | Sequence 5’-3’ | Purpose |
| --- | --- | --- |
| 472 | GGCAAGCATCACAGCTTCACAC | GBSS1 PCR amplification |
| 384 | CGATGTCCGCGGGCCTAAATG | GBSS1 PCR amplification |
| 589 | CACTAGACACCAAATCAACCTTG | GBSS1 Sequencing |
| 475 | CTGTTAACAAGCTTGATGGGCTCC | GBSS1 IDAA PCR |
| FAM481 | FAM-ACTTACTGCAAGGGCTGGTGG | GBSS1 IDAA PCR |
| 567 | CTCTATCGGACTTGCGATCGGAACCAACTC | Site directed mutagenesis (D🡪A) |
| 568 | GAGTTGGTTCCGATCGCAAGTCCGATAGAG | Site directed mutagenesis (D🡪A) |

FAM481 - 5’ end labeled with Fluorescein amidite (FAM)

**Plasmid and base editor sequences**

*Sp*Cas9 enzyme in blue

PPDK35S promoter in pink

NOS terminator in dark grey

Intron in Cas9 in lower case letters

*Solanum tuberosum* U6 promoter in green

gRNA in red

gRNA scaffold in yellow

D10A mutation in black

Deaminases hAPOBEC3A, rAPOBEC1 and evo_*Pm*CDA1 in purple

SG extended XTEN linker in teal

Nucleoplasmin NLS in dark red

SGGS linker in dark blue

UGI in dark yellow

SV40 NLS in light grey

**>** *Sp*Cas9/*St*U6-1::sgRNA1 nickase

CTCGTGATACGCCTATTTTTATAGGTTAATGTCATGATAATAATGGTTTCTTAGACGTCAGGTGGCACTTTTCGGGGAAATGTGCGCGGAACCCCTATTTGTTTATTTTTCTAAATACATTCAAATATGTATCCGCTCATGAGACAATAACCCTGATAAATGCTTCAATAATATTGAAAAAGGAAGAGTATGAGTATTCAACATTTCCGTGTCGCCCTTATTCCCTTTTTTGCGGCATTTTGCCTTCCTGTTTTTGCTCACCCAGAAACGCTGGTGAAAGTAAAAGATGCTGAAGATCAGTTGGGTGCACGAGTGGGTTACATCGAACTGGATCTCAACAGCGGTAAGATCCTTGAGAGTTTTCGCCCCGAAGAACGTTTTCCAATGATGAGCACTTTTAAAGTTCTGCTATGTGGCGCGGTATTATCCCGTATTGACGCCGGGCAAGAGCAACTCGGTCGCCGCATACACTATTCTCAGAATGACTTGGTTGAGTACTCACCAGTCACAGAAAAGCATCTTACGGATGGCATGACAGTAAGAGAATTATGCAGTGCTGCCATAACCATGAGTGATAACACTGCGGCCAACTTACTTCTGACAACGATCGGAGGACCGAAGGAGCTAACCGCTTTTTTGCACAACATGGGGGATCATGTAACTCGCCTTGATCGTTGGGAACCGGAGCTGAATGAAGCCATACCAAACGACGAGCGTGACACCACGATGCCTGTAGCAATGGCAACAACGTTGCGCAAACTATTAACTGGCGAACTACTTACTCTAGCTTCCCGGCAACAATTAATAGACTGGATGGAGGCGGATAAAGTTGCAGGACCACTTCTGCGCTCGGCCCTTCCGGCTGGCTGGTTTATTGCTGATAAATCTGGAGCCGGTGAGCGTGGGTCTCGCGGTATCATTGCAGCACTGGGGCCAGATGGTAAGCCCTCCCGTATCGTAGTTATCTACACGACGGGGAGTCAGGCAACTATGGATGAACGAAATAGACAGATCGCTGAGATAGGTGCCTCACTGATTAAGCATTGGTAACTGTCAGACCAAGTTTACTCATATATACTTTAGATTGATTTAAAACTTCATTTTTAATTTAAAAGGATCTAGGTGAAGATCCTTTTTGATAATCTCATGACCAAAATCCCTTAACGTGAGTTTTCGTTCCACTGAGCGTCAGACCCCGTAGAAAAGATCAAAGGATCTTCTTGAGATCCTTTTTTTCTGCGCGTAATCTGCTGCTTGCAAACAAAAAAACCACCGCTACCAGCGGTGGTTTGTTTGCCGGATCAAGAGCTACCAACTCTTTTTCCGAAGGTAACTGGCTTCAGCAGAGCGCAGATACCAAATACTGTCCTTCTAGTGTAGCCGTAGTTAGGCCACCACTTCAAGAACTCTGTAGCACCGCCTACATACCTCGCTCTGCTAATCCTGTTACCAGTGGCTGCTGCCAGTGGCGATAAGTCGTGTCTTACCGGGTTGGACTCAAGACGATAGTTACCGGATAAGGCGCAGCGGTCGGGCTGAACGGGGGGTTCGTGCACACAGCCCAGCTTGGAGCGAACGACCTACACCGAACTGAGATACCTACAGCGTGAGCTATGAGAAAGCGCCACGCTTCCCGAAGGGAGAAAGGCGGACAGGTATCCGGTAAGCGGCAGGGTCGGAACAGGAGAGCGCACGAGGGAGCTTCCAGGGGGAAACGCCTGGTATCTTTATAGTCCTGTCGGGTTTCGCCACCTCTGACTTGAGCGTCGATTTTTGTGATGCTCGTCAGGGGGGCGGAGCCTATGGAAAAACGCCAGCAACGCGGCCTTTTTACGGTTCCTGGCCTTTTGCTGGCCTTTTGCTCACATGTTCTTTCCTGCGTTATCCCCTGATTCTGTGGATAACCGTATTACCGCCTTTGAGTGAGCTGATACCGCTCGCCGCAGCCGAACGACCGAGCGCAGCGAGTCAGTGAGCGAGGAAGCGGAAGAGCGCCCAATACGCAAACCGCCTCTCCCCGCGCGTTGGCCGATTCATTAATGCAGCTGGCACGACAGGTTTCCCGACTGGAAAGCGGGCAGTGAGCGCAACGCAATTAATGTGAGTTAGCTCACTCATTAGGCACCCCAGGCTTTACACTTTATGCTTCCGGCTCGTATGTTGTGTGGAATTGTGAGCGGATAACAATTTCACACAGGAAACAGCTATGACCATGATTACGAATTCTCATGTTTGACAGCTTATCATCGGATCTAGTAACATAGATGACACCGCGCGCGATAATTTATCCTAGTTTGCGCGCTATATTTTGTTTTCTATCGCGTATTAAATGTATAATTGCGGGACTCTAATCATAAAAACCCATCTCATAAATAACGTCATGCATTACATGTTAATTATTACATGCTTAACGTAATCAACAGAAATATATATGATAATCATCGCAAGACCGGCAACAGGATTCAATCTTAAGAAACTTTATTGCCAAATGTTTGAACGATCTGCAGTCACTTCTTCTTCTTAGCCTGTCCAGCCTTCTTGGTAGCAGCTGGTCTCTTATCTCCTCCAAGCTGAGAAAGATCGATTCTGGTCTCGTAAAGTCCGGTGATAGACTGGTGGATAAGGGTAGCATCAAGAACCTCCTTGGTAGAGGTGTATCTTTTTCTATCGATGGTGGTATCGAAGTACTTGAAAGCAGCTGGAGCACCAAGGTTGGTAAGGGTGAAAAGGTGGATGATGTTCTCAGCCTGCTCTCTGATTGGCTTATCTCTGTGCTTGTTGTAAGCAGAAAGAACCTTATCAAGGTTAGCATCAGCAAGGATAACTCTCTTAGAGAACTCAGAGATTTGCTCGATGATCTCATCAAGGTAGTGCTTGTGCTGCTCAACGAAAAGCTGCTTCTGCTCGTTATCCTCTGGAGATCCCTTCAACTTCTCGTAGTGAGAAGCAAGGTAAAGGAAGTTAACGTACTTAGATGGAAGAGCAAGCTCGTTTCCCTTCTGAAGCTCTCCAGCAGAAGCAAGCATTCTCTTTCTTCCGTTCTCAAGCTCGAAAAGAGAGTACTTTGGCAACTTGATGATAAGATCCTTCTTAACCTCCTTGTATCCCTTAGCCTCAAGGAAATCGATTGGGTTCTTCTCGAAAGAAGAACGCTCCATGATGGTGATTCCAAGAAGCTCCTTAACAGACTTCAACTTCTTAGACTTTCCCTTCTCAACCTTAGCAACAACAAGAACAGAGTAAGCAACGGTTGGAGAATCGAATCCTCCGTACTTCTTTGGGTCCCAATCCTTCTTTCTAGCGATCAACTTATCAGAGTTTCTCTTTGGAAGGATAGACTCCTTAGAGAATCCTCCGGTCTGAACCTCGGTTTTCTTAACGATGTTAACCTGTGGCATAGAAAGAACCTTTCTAACGGTAGCGAAATCTCTTCCCTTATCCCAAACGATCTCTCCGGTCTCTCCGTTGGTCTCGATAAGTGGTCTCTTTCTGATCTCTCCGTTAGCAAGGGTGATCTCGGTCTTGAAGAAGTTCATGATGTTAGAGTAGAAGAAGTACTTAGCGGTAGCCTTTCCGATCTCCTGCTCAGACTTAGCGATCATCTTTCTAACATCGTAAACCTTGTAATCTCCGTAAACGAACTCAGACTCCAACTTTGGGTACTTCTTGATAAGAGCGGTTCCAACAACAGCGTTAAGGTAAGCATCGTGAGCGTGGTGGTAGTTGTTGATCTCTCTAACCTTGTAGAACTGGAAATCCTTTCTGAAATCAGAAACCAACTTAGACTTCAAGGTGATAACCTTAACCTCTCTGATCAACTTATCGTTCTCATCGTACTTGGTGTTCATACGAGAATCAAGGATCTGAGCAACGTGCTTGGTGATCTGTCTGGTCTCAACAAGCTGTCTCTTGATGAATCCAGCCTTATCAAGCTCAGAAAGTCCTCCTCTCTCAGCCTTGGTAAGGTTATCGAACTTTCTCTGGGTGATCAACTTAGCGTTAAGAAGCTGTCTCCAGTAGTTCTTCATCTTCTTAACAACCTCCTCAGATGGAACGTTATCAGACTTTCCTCTGTTCTTATCAGAACGGGTAAGAACCTTGTTATCGATAGAATCATCCTTCAAGAAAGACTGTGGAACGATGTGATCAACATCGTAATCAGAAAGTCTGTTGATGTCAAGCTCCTGATCAACGTACATATCTCTTCCGTTCTGAAGGTAGTAAAGGTACAACTTCTCGTTCTGAAGCTGGGTGTTCTCAACTGGGTGCTCCTTCAAGATTTGAGATCCAAGCTCCTTGATTCCCTCCTCGATTCTCTTCATTCTCTCACGAGAGTTCTTCTGTCCCTTCTGGGTGGTCTGGTTCTCTCTAGCCATCTCGATAACGATGTTCTCTGGCTTGTGTCTACCCATAACCTTAACAAGCTCATCAACAACCTTAACGGTCTGAAGGATTCCCTTCTTGATAGCTGGAGATCCAGCAAGGTTAGCGATGTGCTCGTGAAGAGAATCTCCCTGTCCAGAAACCTGAGCCTTCTGGATGTCCTCCTTGAAGGTAAGAGAATCATCGTGGATAAGCTGCATGAAGTTTCTGTTAGCGAATCCATCAGACTTCAAGAAATCAAGGATGGTCTTTCCAGACTGCTTATCTCTGATTCCGTTGATCAACTTACGAGAAAGTCTACCCCATCCGGTGTATCTTCTTCTCTTCAACTGCTTCATAACCTTATCATCGAAAAGGTGAGCGTAGGTCTTGAGTCTCTCCTCGATCATCTCTCTATCCTCGAAAAGGGTAAGGGTAAGAACGATGTCCTCAAGGATGTCCTCGTTCTCCTCGTTATCAAGGAAATCCTTATCCTTGATGATCTTCAAAAGATCGTGGTAGGTTCCAAGAGAAGCGTTGAATCTATCCTCAACTCCAGAGATTTCAACAGAATCGAAGCACTCGATCTTCTTGAAGTAATCCTCCTTCAACTGCTTAACGGTAACCTTTCTGTTGGTCTTGAAAAGAAGATCAACGATAGCCTTCTTCTGCTCTCCAGAAAGGAAAGCTGGCTTTCTCATTCCCTCGGTAACGTACTTAACCTTGGTAAGCTCGTTGTAAACGGTGAAGTACTCGTAAAGAAGAGAGTGCTTTGGAAGAACCTTCTCGTTTGGAAGGTTCTTATCGAAGTTGGTCATTCTCTCGATGAAAGACTGAGCAGAAGCTCCCTTATCAACAACctgcacatcaacaaattttggtcatatattagaaaagttataaattaaaatatacacacttataaactacagaaaagcaattgctatatactacattcttttattttgaaaaaaatatttgaaatattatattactactaattaatgataattattatatatatatcaaaggtagaagcagaaacttacCTCCTCGAAGTTCCAAGGGGTGATGGTCTCCTCAGACTTTCTGGTCATCCAAGCGAAACGAGAGTTTCCTCTAGCAAGTGGTCCAACGTAGTATGGGATTCTGAAGGTAAGGATCTTCTCGATCTTCTCTCTGTTATCCTTCAAGAATGGGTAGAAATCCTCCTGTCTACGAAGGATAGCGTGAAGCTCTCCAAGGTGGATCTGGTGTGGGATAGATCCGTTATCGAAGGTTCTCTGCTTTCTAAGAAGATCCTCTCTGTTCAACTTAACAAGAAGCTCCTCGGTTCCATCCATCTTCTCAAGGATTGGCTTGATGAACTTGTAGAACTCCTCCTGAGAAGCTCCTCCATCGATGTATCCAGCGTATCCGTTCTTAGACTGATCGAAGAAGATTTCCTTGTACTTCTCTGGAAGCTGCTGTCTAACAAGAGCCTTCAAAAGGGTAAGATCCTGGTGGTGCTCATCGTATCTCTTGATCATAGAAGCAGAAAGTGGAGCCTTGGTGATCTCGGTGTTAACTCTAAGGATGTCAGAAAGAAGGATAGCATCAGAAAGGTTCTTAGCAGCAAGGAAAAGATCAGCGTACTGATCTCCGATCTGAGCAAGAAGGTTATCAAGATCATCATCGTAGGTATCCTTAGAAAGCTGCAACTTAGCATCCTCAGCAAGATCGAAGTTAGACTTGAAGTTTGGGGTAAGTCCAAGAGAAAGAGCGATAAGGTTTCCGAAAAGTCCGTTCTTCTTCTCTCCTGGAAGCTGAGCGATAAGGTTCTCAAGTCTACGAGACTTAGAAAGTCTAGCAGAAAGGATAGCCTTAGCATCAACTCCAGAAGCGTTGATTGGGTTCTCCTCGAAAAGCTGGTTGTAGGTCTGAACAAGCTGGATGAACAACTTATCAACATCAGAGTTATCTGGGTTAAGGTCTCCCTCGATAAGGAAGTGTCCTCTGAACTTGATCATGTGAGCAAGAGCAAGGTAGATAAGTCTAAGATCAGCCTTATCGGTAGAATCAACCAACTTCTTTCTAAGGTGGTAGATGGTTGGGTACTTCTCGTGGTAAGCAACCTCATCAACGATGTTTCCGAAGATTGGGTGTCTCTCGTGCTTCTTATCCTCCTCAACAAGGAAAGACTCCTCAAGTCTGTGGAAGAAAGAATCATCAACCTTAGCCATCTCGTTAGAGAAGATTTCCTGAAGGTAGCAGATTCTGTTCTTTCTTCTGGTGTATCTTCTTCTAGCGGTTCTCTTCAATCTGGTAGCCTCAGCGGTCTCTCCAGAATCGAAAAGAAGAGCACCGATAAGGTTCTTCTTGATAGAGTGTCTATCGGTGTTTCCAAGAACCTTGAACTTCTTAGATGGAACCTTGTACTCATCGGTGATAACAGCCCATCCAACAGAGTTGGTTCCGATCGCAAGTCCGATAGAGTACTTCTTATCAGCAGCTGGAACTCCGTGGATTCCAACCTTTCTCTTCTTCTTTGGAGCCATCTTATCATCATCATCCTTGTAATCCTTATCATCATCATCCTTGTAATCCATGGATCCACGGAGCAAGGGGAGAGGGAGCCGAGAGCTGCTTACTACGTGACCGCCCCGCGCCTGCTGCTGTCCGGAGCTCCAACGGAGCTGGAGAGGGGGTGTGTGTGGCGAGAGAGTACAGCGAAAAGGTGAGAGGCGAACGGGTGAATCCTGCACGCGGAATGCCTTTATTCTTATACCATCCGTGGGGCGCGACCCAAGCTTGGGCCCAAGCTTGGGGCGAAGGATAGTGGGATTGTGCGTCATCCCTTACGTCAGTGGAGATATCACATCAATCCACTTGCTTTGAAGACGTGGTTGGAACGTCTTCTTTTTCCACGATGCTCCTTGTGGGTGGGGGTCCATCTTTGGGACCACTGTCGGCAGAGGCATCTTGAACGATAGCCTTTCCTTTATCGCAATGATGGCATTTGTAGGTGCCACCTTCCTTTTCTACTGTCCTTTTGATGAAGTGACAGATAGCTGGGCAATGGAATCCGAGGAGGTTTCCCGATATTACCCTTTGTTGAAAAGTCTCAATAGCCCTTTGGTCTTCTGAGACTGTATCTTTGATATTCTTGGAGTAAGCTTCTCGAGAGCTTGGCACTGGCCGTCGTTTTACAACGTCGTGACTGGGAAAACCCTGGCGTTACCCAACTTAATCGCCTTGCAGCACATCCCCCTTTCGCCAGCTGGCGTAATAGCCACGGAGCAAGGGGAGAGGGAGCCGAGAGCTGCTTACTACGTGACCGCCGAAGAGGCCCGCACCGATCGCCCTTCCCAACAGTTGCGCAGCCTGAATGGCGAATGGCGGGGATCCTCTAGTCGACAGAAATCTCAAAATTCCGGCAGAACAATTTTGAATCTCGATCCGTAGTTTCTTTTGTGTAAAATTTCAACGGTTAAACCGAATGGTCGTTTTATAACAGTTTAACATATTTACAAATTGACAACCGTTAAGTCGAATCAATAGACTTCAAAACCAAACAAACCGATACGGGCACTAATTTCAATAACCAAATGGTACAAGTTGAATATGGGGGCAAATCTGGACTCTAGGCTTAGTTGGGCTCTATGTGCATGAATGAACATATAAGCAAGAGCAAAAACCTGTAGCTAGGTCCAGGCCCATGCCTTTGGAAAAACTCAATGTGCTAATTCTCCCTCATCGTCTGCAGAGAGAAGCTTCGCTGTGTTTATATAATTGAACAGTAACATGTATGCTTGGTCCTTGGAGCAAAACTGGGTTTTAGAGCTAGAAATAGCAAGTTAAAATAAGGCTAGTCCGTTATCAACTTGAAAAAGTGGCACCGAGTCGGTGCTTTTTTTCTAGAGGCCTGATGCGGTATTTTCTCCTTACGCATCTGTGCGGTATTTCACACCGCATATGGTGCACTCTCAGTACAATCTGCTCTGATGCCGCATAGTTAAGCCAGCCCCGACACCCGCCAACACCCGCTGACGCGCCCTGACGGGCTTGTCTGCTCCCGGCATCCGCTTACAGACAAGCTGTGACCGTCTCCGGGAGCTGCATGTGTCAGAGGTTTTCACCGTCATCACCGAAACGCGCGAGACGAAAGGGC

**>** hAPOBEC3A (A3A)

GCCCTTTCGTCTCGCGCGTTTCGGTGATGACGGTGAAAACCTCTGACACATGCAGCTCCCGGAGACGGTCACAGCTTGTCTGTAAGCGGATGCCGGGAGCAGACAAGCCCGTCAGGGCGCGTCAGCGGGTGTTGGCGGGTGTCGGGGCTGGCTTAACTATGCGGCATCAGAGCAGATTGTACTGAGAGTGCACCATATGCGGTGTGAAATACCGCACAGATGCGTAAGGAGAAAATACCGCATCAGGCCTCTAGAAAAAAAGCACCGACTCGGTGCCACTTTTTCAAGTTGATAACGGACTAGCCTTATTTTAACTTGCTATTTCTAGCTCTAAAACCCAGTTTTGCTCCAAGGACCAAGCATACATGTTACTGTTCAATTATATAAACACAGCGAAGCTTCTCTCTGCAGACGATGAGGGAGAATTAGCACATTGAGTTTTTCCAAAGGCATGGGCCTGGACCTAGCTACAGGTTTTTGCTCTTGCTTATATGTTCATTCATGCACATAGAGCCCAACTAAGCCTAGAGTCCAGATTTGCCCCCATATTCAACTTGTACCATTTGGTTATTGAAATTAGTGCCCGTATCGGTTTGTTTGGTTTTGAAGTCTATTGATTCGACTTAACGGTTGTCAATTTGTAAATATGTTAAACTGTTATAAAACGACCATTCGGTTTAACCGTTGAAATTTTACACAAAAGAAACTACGGATCGAGATTCAAAATTGTTCTGCCGGAATTTTGAGATTTCTGTCGACTAGAGGATCCCCGCCATTCGCCATTCAGGCTGCGCAACTGTTGGGAAGGGCGATCGGTGCGGGCCTCTTCGCTATTACGCCAGCTGGCGAAAGGGGGATGTGCTGCAAGGCGATTAAGTTGGGTAACGCCAGGGTTTTCCCAGTCACGACGTTGTAAAACGACGGCCAGTGCCAAGCTCTCGAGAAGCTTACTCCAAGAATATCAAAGATACAGTCTCAGAAGACCAAAGGGCTATTGAGACTTTTCAACAAAGGGTAATATCGGGAAACCTCCTCGGATTCCATTGCCCAGCTATCTGTCACTTCATCAAAAGGACAGTAGAAAAGGAAGGTGGCACCTACAAATGCCATCATTGCGATAAAGGAAAGGCTATCGTTCAAGATGCCTCTGCCGACAGTGGTCCCAAAGATGGACCCCCACCCACAAGGAGCATCGTGGAAAAAGAAGACGTTCCAACCACGTCTTCAAAGCAAGTGGATTGATGTGATATCTCCACTGACGTAAGGGATGACGCACAATCCCACTATCCTTCGCCCCAAGCTTGGGCCCAAGCTTGGGTCGCGCCCCACGGATGGTATAAGAATAAAGGCATTCCGCGTGCAGGATTCACCCGTTCGCCTCTCACCTTTTCGCTGTACTCTCTCGCCACACACACCCCCTCTCCAGCTCCGTTGGAGCTCCGGACAGCAGCAGGCGCGGGGCGGTCACGTAGTAAGCAGCTCTCGGCTCCCTCTCCCCTTGCTCCGTGGATCCATGGAGGCTTCTCCAGCAAGTGGCCCTAGGCATCTTATGGACCCTCATATTTTCACTTCAAACTTCAATAATGGAATTGGAAGACACAAAACATACTTGTGCTATGAAGTGGAGAGATTGGATAATGGCACCTCTGTCAAAATGGATCAGCACAGAGGCTTCTTGCACAACCAGGCTAAGAATCTGCTTTGTGGATTTTATGGTAGACATGCTGAGCTGCGTTTCTTAGACTTGGTACCCTCACTACAACTAGATCCTGCTCAAATTTACCGTGTGACATGGTTTATTTCATGGTCTCCTTGTTTTTCTTGGGGTTGTGCTGGAGAAGTAAGGGCATTTCTTCAAGAAAATACTCATGTTCGGTTAAGGATATTTGCCGCTCGAATATATGACTATGATCCATTATACAAAGAAGCGTTGCAAATGCTTAGAGATGCAGGTGCACAAGTTTCCATCATGACTTATGATGAGTTCAAGCATTGTTGGGACACATTTGTTGATCATCAGGGTTGCCCATTTCAGCCATGGGATGGACTTGATGAACATTCTCAAGCTCTGAGTGGACGTCTCCGCGCCATCCTCCAGAATCAAGGAAACTCGGGTGGGAGCAGCGGAGGATCATCGGGCAGTGAAACACCGGGGACGTCAGAGAGCGCTACTCCTGAATCCTCAGGTGGGAGTAGTGGTGGTTCTGATAAGAAATACTCTATCGGACTTGCGATCGGAACCAACTCTGTTGGATGGGCTGTTATCACCGATGAGTACAAGGTTCCATCTAAGAAGTTCAAGGTTCTTGGAAACACCGATAGACACTCTATCAAGAAGAACCTTATCGGTGCTCTTCTTTTCGATTCTGGAGAGACCGCTGAGGCTACCAGATTGAAGAGAACCGCTAGAAGAAGATACACCAGAAGAAAGAACAGAATCTGCTACCTTCAGGAAATCTTCTCTAACGAGATGGCTAAGGTTGATGATTCTTTCTTCCACAGACTTGAGGAGTCTTTCCTTGTTGAGGAGGATAAGAAGCACGAGAGACACCCAATCTTCGGAAACATCGTTGATGAGGTTGCTTACCACGAGAAGTACCCAACCATCTACCACCTTAGAAAGAAGTTGGTTGATTCTACCGATAAGGCTGATCTTAGACTTATCTACCTTGCTCTTGCTCACATGATCAAGTTCAGAGGACACTTCCTTATCGAGGGAGACCTTAACCCAGATAACTCTGATGTTGATAAGTTGTTCATCCAGCTTGTTCAGACCTACAACCAGCTTTTCGAGGAGAACCCAATCAACGCTTCTGGAGTTGATGCTAAGGCTATCCTTTCTGCTAGACTTTCTAAGTCTCGTAGACTTGAGAACCTTATCGCTCAGCTTCCAGGAGAGAAGAAGAACGGACTTTTCGGAAACCTTATCGCTCTTTCTCTTGGACTTACCCCAAACTTCAAGTCTAACTTCGATCTTGCTGAGGATGCTAAGTTGCAGCTTTCTAAGGATACCTACGATGATGATCTTGATAACCTTCTTGCTCAGATCGGAGATCAGTACGCTGATCTTTTCCTTGCTGCTAAGAACCTTTCTGATGCTATCCTTCTTTCTGACATCCTTAGAGTTAACACCGAGATCACCAAGGCTCCACTTTCTGCTTCTATGATCAAGAGATACGATGAGCACCACCAGGATCTTACCCTTTTGAAGGCTCTTGTTAGACAGCAGCTTCCAGAGAAGTACAAGGAAATCTTCTTCGATCAGTCTAAGAACGGATACGCTGGATACATCGATGGAGGAGCTTCTCAGGAGGAGTTCTACAAGTTCATCAAGCCAATCCTTGAGAAGATGGATGGAACCGAGGAGCTTCTTGTTAAGTTGAACAGAGAGGATCTTCTTAGAAAGCAGAGAACCTTCGATAACGGATCTATCCCACACCAGATCCACCTTGGAGAGCTTCACGCTATCCTTCGTAGACAGGAGGATTTCTACCCATTCTTGAAGGATAACAGAGAGAAGATCGAGAAGATCCTTACCTTCAGAATCCCATACTACGTTGGACCACTTGCTAGAGGAAACTCTCGTTTCGCTTGGATGACCAGAAAGTCTGAGGAGACCATCACCCCTTGGAACTTCGAGGAGgtaagtttctgcttctacctttgatatatatataataattatcattaattagtagtaatataatatttcaaatatttttttcaaaataaaagaatgtagtatatagcaattgcttttctgtagtttataagtgtgtatattttaatttataacttttctaatatatgaccaaaatttgttgatgtgcagGTTGTTGATAAGGGAGCTTCTGCTCAGTCTTTCATCGAGAGAATGACCAACTTCGATAAGAACCTTCCAAACGAGAAGGTTCTTCCAAAGCACTCTCTTCTTTACGAGTACTTCACCGTTTACAACGAGCTTACCAAGGTTAAGTACGTTACCGAGGGAATGAGAAAGCCAGCTTTCCTTTCTGGAGAGCAGAAGAAGGCTATCGTTGATCTTCTTTTCAAGACCAACAGAAAGGTTACCGTTAAGCAGTTGAAGGAGGATTACTTCAAGAAGATCGAGTGCTTCGATTCTGTTGAAATCTCTGGAGTTGAGGATAGATTCAACGCTTCTCTTGGAACCTACCACGATCTTTTGAAGATCATCAAGGATAAGGATTTCCTTGATAACGAGGAGAACGAGGACATCCTTGAGGACATCGTTCTTACCCTTACCCTTTTCGAGGATAGAGAGATGATCGAGGAGAGACTCAAGACCTACGCTCACCTTTTCGATGATAAGGTTATGAAGCAGTTGAAGAGAAGAAGATACACCGGATGGGGTAGACTTTCTCGTAAGTTGATCAACGGAATCAGAGATAAGCAGTCTGGAAAGACCATCCTTGATTTCTTGAAGTCTGATGGATTCGCTAACAGAAACTTCATGCAGCTTATCCACGATGATTCTCTTACCTTCAAGGAGGACATCCAGAAGGCTCAGGTTTCTGGACAGGGAGATTCTCTTCACGAGCACATCGCTAACCTTGCTGGATCTCCAGCTATCAAGAAGGGAATCCTTCAGACCGTTAAGGTTGTTGATGAGCTTGTTAAGGTTATGGGTAGACACAAGCCAGAGAACATCGTTATCGAGATGGCTAGAGAGAACCAGACCACCCAGAAGGGACAGAAGAACTCTCGTGAGAGAATGAAGAGAATCGAGGAGGGAATCAAGGAGCTTGGATCTCAAATCTTGAAGGAGCACCCAGTTGAGAACACCCAGCTTCAGAACGAGAAGTTGTACCTTTACTACCTTCAGAACGGAAGAGATATGTACGTTGATCAGGAGCTTGACATCAACAGACTTTCTGATTACGATGTTGATCACATCGTTCCACAGTCTTTCTTGAAGGATGATTCTATCGATAACAAGGTTCTTACCCGTTCTGATAAGAACAGAGGAAAGTCTGATAACGTTCCATCTGAGGAGGTTGTTAAGAAGATGAAGAACTACTGGAGACAGCTTCTTAACGCTAAGTTGATCACCCAGAGAAAGTTCGATAACCTTACCAAGGCTGAGAGAGGAGGACTTTCTGAGCTTGATAAGGCTGGATTCATCAAGAGACAGCTTGTTGAGACCAGACAGATCACCAAGCACGTTGCTCAGATCCTTGATTCTCGTATGAACACCAAGTACGATGAGAACGATAAGTTGATCAGAGAGGTTAAGGTTATCACCTTGAAGTCTAAGTTGGTTTCTGATTTCAGAAAGGATTTCCAGTTCTACAAGGTTAGAGAGATCAACAACTACCACCACGCTCACGATGCTTACCTTAACGCTGTTGTTGGAACCGCTCTTATCAAGAAGTACCCAAAGTTGGAGTCTGAGTTCGTTTACGGAGATTACAAGGTTTACGATGTTAGAAAGATGATCGCTAAGTCTGAGCAGGAGATCGGAAAGGCTACCGCTAAGTACTTCTTCTACTCTAACATCATGAACTTCTTCAAGACCGAGATCACCCTTGCTAACGGAGAGATCAGAAAGAGACCACTTATCGAGACCAACGGAGAGACCGGAGAGATCGTTTGGGATAAGGGAAGAGATTTCGCTACCGTTAGAAAGGTTCTTTCTATGCCACAGGTTAACATCGTTAAGAAAACCGAGGTTCAGACCGGAGGATTCTCTAAGGAGTCTATCCTTCCAAAGAGAAACTCTGATAAGTTGATCGCTAGAAAGAAGGATTGGGACCCAAAGAAGTACGGAGGATTCGATTCTCCAACCGTTGCTTACTCTGTTCTTGTTGTTGCTAAGGTTGAGAAGGGAAAGTCTAAGAAGTTGAAGTCTGTTAAGGAGCTTCTTGGAATCACCATCATGGAGCGTTCTTCTTTCGAGAAGAACCCAATCGATTTCCTTGAGGCTAAGGGATACAAGGAGGTTAAGAAGGATCTTATCATCAAGTTGCCAAAGTACTCTCTTTTCGAGCTTGAGAACGGAAGAAAGAGAATGCTTGCTTCTGCTGGAGAGCTTCAGAAGGGAAACGAGCTTGCTCTTCCATCTAAGTACGTTAACTTCCTTTACCTTGCTTCTCACTACGAGAAGTTGAAGGGATCTCCAGAGGATAACGAGCAGAAGCAGCTTTTCGTTGAGCAGCACAAGCACTACCTTGATGAGATCATCGAGCAAATCTCTGAGTTCTCTAAGAGAGTTATCCTTGCTGATGCTAACCTTGATAAGGTTCTTTCTGCTTACAACAAGCACAGAGATAAGCCAATCAGAGAGCAGGCTGAGAACATCATCCACCTTTTCACCCTTACCAACCTTGGTGCTCCAGCTGCTTTCAAGTACTTCGATACCACCATCGATAGAAAAAGATACACCTCTACCAAGGAGGTTCTTGATGCTACCCTTATCCACCAGTCTATCACCGGACTTTACGAGACCAGAATCGATCTTTCTCAGCTTGGAGGAGATAAGAGACCAGCTGCTACCAAGAAGGCTGGACAGGCAAAGAAGAAAAAAACAAGAGACTCCGGGGGCAGCACAAATCTCTCTGATATTATTGAGAAAGAAACTGGAAAGCAGTTAGTCATTCAAGAATCTATCTTGATGCTTCCTGAGGAAGTTGAAGAAGTGATTGGAAACAAACCAGAGAGTGACATACTTGTTCATACCGCTTATGATGAGTCTACTGATGAAAATGTTATGTTACTAACGTCAGATGCTCCTGAATACAAGCCATGGGCACTGGTAATACAAGATTCAAATGGAGAGAACAAGATCAAAATGTTGAGTGGTGGTTCGCCCAAAAAGAAAAGGAAGGTGTAGCTGCAGATCGTTCAAACATTTGGCAATAAAGTTTCTTAAGATTGAATCCTGTTGCCGGTCTTGCGATGATTATCATATATATTTCTGTTGATTACGTTAAGCATGTAATAATTAACATGTAATGCATGACGTTATTTATGAGATGGGTTTTTATGATTAGAGTCCCGCAATTATACATTTAATACGCGATAGAAAACAAAATATAGCGCGCAAACTAGGATAAATTATCGCGCGCGGTGTCATCTATGTTACTAGATCCGATGATAAGCTGTCAAACATGAGAATTCGTAATCATGGTCATAGCTGTTTCCTGTGTGAAATTGTTATCCGCTCACAATTCCACACAACATACGAGCCGGAAGCATAAAGTGTAAAGCCTGGGGTGCCTAATGAGTGAGCTAACTCACATTAATTGCGTTGCGCTCACTGCCCGCTTTCCAGTCGGGAAACCTGTCGTGCCAGCTGCATTAATGAATCGGCCAACGCGCGGGGAGAGGCGGTTTGCGTATTGGGCGCTCTTCCGCTTCCTCGCTCACTGACTCGCTGCGCTCGGTCGTTCGGCTGCGGCGAGCGGTATCAGCTCACTCAAAGGCGGTAATACGGTTATCCACAGAATCAGGGGATAACGCAGGAAAGAACATGTGAGCAAAAGGCCAGCAAAAGGCCAGGAACCGTAAAAAGGCCGCGTTGCTGGCGTTTTTCCATAGGCTCCGCCCCCCTGACGAGCATCACAAAAATCGACGCTCAAGTCAGAGGTGGCGAAACCCGACAGGACTATAAAGATACCAGGCGTTTCCCCCTGGAAGCTCCCTCGTGCGCTCTCCTGTTCCGACCCTGCCGCTTACCGGATACCTGTCCGCCTTTCTCCCTTCGGGAAGCGTGGCGCTTTCTCATAGCTCACGCTGTAGGTATCTCAGTTCGGTGTAGGTCGTTCGCTCCAAGCTGGGCTGTGTGCACGAACCCCCCGTTCAGCCCGACCGCTGCGCCTTATCCGGTAACTATCGTCTTGAGTCCAACCCGGTAAGACACGACTTATCGCCACTGGCAGCAGCCACTGGTAACAGGATTAGCAGAGCGAGGTATGTAGGCGGTGCTACAGAGTTCTTGAAGTGGTGGCCTAACTACGGCTACACTAGAAGGACAGTATTTGGTATCTGCGCTCTGCTGAAGCCAGTTACCTTCGGAAAAAGAGTTGGTAGCTCTTGATCCGGCAAACAAACCACCGCTGGTAGCGGTGGTTTTTTTGTTTGCAAGCAGCAGATTACGCGCAGAAAAAAAGGATCTCAAGAAGATCCTTTGATCTTTTCTACGGGGTCTGACGCTCAGTGGAACGAAAACTCACGTTAAGGGATTTTGGTCATGAGATTATCAAAAAGGATCTTCACCTAGATCCTTTTAAATTAAAAATGAAGTTTTAAATCAATCTAAAGTATATATGAGTAAACTTGGTCTGACAGTTACCAATGCTTAATCAGTGAGGCACCTATCTCAGCGATCTGTCTATTTCGTTCATCCATAGTTGCCTGACTCCCCGTCGTGTAGATAACTACGATACGGGAGGGCTTACCATCTGGCCCCAGTGCTGCAATGATACCGCGAGACCCACGCTCACCGGCTCCAGATTTATCAGCAATAAACCAGCCAGCCGGAAGGGCCGAGCGCAGAAGTGGTCCTGCAACTTTATCCGCCTCCATCCAGTCTATTAATTGTTGCCGGGAAGCTAGAGTAAGTAGTTCGCCAGTTAATAGTTTGCGCAACGTTGTTGCCATTGCTACAGGCATCGTGGTGTCACGCTCGTCGTTTGGTATGGCTTCATTCAGCTCCGGTTCCCAACGATCAAGGCGAGTTACATGATCCCCCATGTTGTGCAAAAAAGCGGTTAGCTCCTTCGGTCCTCCGATCGTTGTCAGAAGTAAGTTGGCCGCAGTGTTATCACTCATGGTTATGGCAGCACTGCATAATTCTCTTACTGTCATGCCATCCGTAAGATGCTTTTCTGTGACTGGTGAGTACTCAACCAAGTCATTCTGAGAATAGTGTATGCGGCGACCGAGTTGCTCTTGCCCGGCGTCAATACGGGATAATACCGCGCCACATAGCAGAACTTTAAAAGTGCTCATCATTGGAAAACGTTCTTCGGGGCGAAAACTCTCAAGGATCTTACCGCTGTTGAGATCCAGTTCGATGTAACCCACTCGTGCACCCAACTGATCTTCAGCATCTTTTACTTTCACCAGCGTTTCTGGGTGAGCAAAAACAGGAAGGCAAAATGCCGCAAAAAAGGGAATAAGGGCGACACGGAAATGTTGAATACTCATACTCTTCCTTTTTCAATATTATTGAAGCATTTATCAGGGTTATTGTCTCATGAGCGGATACATATTTGAATGTATTTAGAAAAATAAACAAATAGGGGTTCCGCGCACATTTCCCCGAAAAGTGCCACCTGACGTCTAAGAAACCATTATTATCATGACATTAACCTATAAAAATAGGCGTATCACGAG

>evo_rAPOBEC1 (rA1)

GCCCTTTCGTCTCGCGCGTTTCGGTGATGACGGTGAAAACCTCTGACACATGCAGCTCCCGGAGACGGTCACAGCTTGTCTGTAAGCGGATGCCGGGAGCAGACAAGCCCGTCAGGGCGCGTCAGCGGGTGTTGGCGGGTGTCGGGGCTGGCTTAACTATGCGGCATCAGAGCAGATTGTACTGAGAGTGCACCATATGCGGTGTGAAATACCGCACAGATGCGTAAGGAGAAAATACCGCATCAGGCCTCTAGAAAAAAAGCACCGACTCGGTGCCACTTTTTCAAGTTGATAACGGACTAGCCTTATTTTAACTTGCTATTTCTAGCTCTAAAACCCAGTTTTGCTCCAAGGACCAAGCATACATGTTACTGTTCAATTATATAAACACAGCGAAGCTTCTCTCTGCAGACGATGAGGGAGAATTAGCACATTGAGTTTTTCCAAAGGCATGGGCCTGGACCTAGCTACAGGTTTTTGCTCTTGCTTATATGTTCATTCATGCACATAGAGCCCAACTAAGCCTAGAGTCCAGATTTGCCCCCATATTCAACTTGTACCATTTGGTTATTGAAATTAGTGCCCGTATCGGTTTGTTTGGTTTTGAAGTCTATTGATTCGACTTAACGGTTGTCAATTTGTAAATATGTTAAACTGTTATAAAACGACCATTCGGTTTAACCGTTGAAATTTTACACAAAAGAAACTACGGATCGAGATTCAAAATTGTTCTGCCGGAATTTTGAGATTTCTGTCGACTAGAGGATCCCCGCCATTCGCCATTCAGGCTGCGCAACTGTTGGGAAGGGCGATCGGTGCGGGCCTCTTCGCTATTACGCCAGCTGGCGAAAGGGGGATGTGCTGCAAGGCGATTAAGTTGGGTAACGCCAGGGTTTTCCCAGTCACGACGTTGTAAAACGACGGCCAGTGCCAAGCTCTCGAGAAGCTTACTCCAAGAATATCAAAGATACAGTCTCAGAAGACCAAAGGGCTATTGAGACTTTTCAACAAAGGGTAATATCGGGAAACCTCCTCGGATTCCATTGCCCAGCTATCTGTCACTTCATCAAAAGGACAGTAGAAAAGGAAGGTGGCACCTACAAATGCCATCATTGCGATAAAGGAAAGGCTATCGTTCAAGATGCCTCTGCCGACAGTGGTCCCAAAGATGGACCCCCACCCACAAGGAGCATCGTGGAAAAAGAAGACGTTCCAACCACGTCTTCAAAGCAAGTGGATTGATGTGATATCTCCACTGACGTAAGGGATGACGCACAATCCCACTATCCTTCGCCCCAAGCTTGGGCCCAAGCTTGGGTCGCGCCCCACGGATGGTATAAGAATAAAGGCATTCCGCGTGCAGGATTCACCCGTTCGCCTCTCACCTTTTCGCTGTACTCTCTCGCCACACACACCCCCTCTCCAGCTCCGTTGGAGCTCCGGACAGCAGCAGGCGCGGGGCGGTCACGTAGTAAGCAGCTCTCGGCTCCCTCTCCCCTTGCTCCGTGGATCCATGAGTTCGAAAACAGGACCAGTTGCTGTTGATCCAACGCTGAGAAGGCGGATTGAGCCACATGAATTTGAGGTCTTCTTTGATCCTAGAGAGCTAAGGAAAGAAACTTGTCTACTTTATGAAATTAATTGGGGTGGTCGCCACAGTATATGGAGACATACTTCTCAAAATACAAACAAGCATGTTGAGGTAAACTTCATTGAAAAGTTTACAACAGAAAGATATTTTTGTCCTAATACTAGATGCTCAATAACATGGTTTCTTTCATGGTCTCCTTGTGGAGAATGTTCTCGAGCTATCACTGAGTTTTTGTCTCGTTATCCGAATGTCACCCTCTTCATCTACATCGCACGTTTGTACCATCTTGCAAATCCAAGGAACAGACAAGGACTTCGTGACTTGATTTCCAGTGGGGTGACAATTCAAATTATGACTGAACAAGAATCTGGGTATTGTTGGCATAATTTTGTGAACTATTCACCCTCAAATGAAAGTCACTGGCCAAGGTACCCTCACTTATGGGTTCGATTATATGTACTGGAGCTTTACTGCATCATATTAGGCTTACCACCGTGCTTGAATATTCTCCGCAGGAAGCAGAGCCAGCTTACCTCATTCACCATAGCCTTGCAAAGCTGTCATTATCAGAGACTCCCTCCTCATATTTTGTGGGCTACTGGTCTAAAATCGGGTGGGAGCAGCGGAGGATCATCGGGCAGTGAAACACCGGGGACGTCAGAGAGCGCTACTCCTGAATCCTCAGGTGGGAGTAGTGGTGGTTCTGATAAGAAATACTCTATCGGACTTGCGATCGGAACCAACTCTGTTGGATGGGCTGTTATCACCGATGAGTACAAGGTTCCATCTAAGAAGTTCAAGGTTCTTGGAAACACCGATAGACACTCTATCAAGAAGAACCTTATCGGTGCTCTTCTTTTCGATTCTGGAGAGACCGCTGAGGCTACCAGATTGAAGAGAACCGCTAGAAGAAGATACACCAGAAGAAAGAACAGAATCTGCTACCTTCAGGAAATCTTCTCTAACGAGATGGCTAAGGTTGATGATTCTTTCTTCCACAGACTTGAGGAGTCTTTCCTTGTTGAGGAGGATAAGAAGCACGAGAGACACCCAATCTTCGGAAACATCGTTGATGAGGTTGCTTACCACGAGAAGTACCCAACCATCTACCACCTTAGAAAGAAGTTGGTTGATTCTACCGATAAGGCTGATCTTAGACTTATCTACCTTGCTCTTGCTCACATGATCAAGTTCAGAGGACACTTCCTTATCGAGGGAGACCTTAACCCAGATAACTCTGATGTTGATAAGTTGTTCATCCAGCTTGTTCAGACCTACAACCAGCTTTTCGAGGAGAACCCAATCAACGCTTCTGGAGTTGATGCTAAGGCTATCCTTTCTGCTAGACTTTCTAAGTCTCGTAGACTTGAGAACCTTATCGCTCAGCTTCCAGGAGAGAAGAAGAACGGACTTTTCGGAAACCTTATCGCTCTTTCTCTTGGACTTACCCCAAACTTCAAGTCTAACTTCGATCTTGCTGAGGATGCTAAGTTGCAGCTTTCTAAGGATACCTACGATGATGATCTTGATAACCTTCTTGCTCAGATCGGAGATCAGTACGCTGATCTTTTCCTTGCTGCTAAGAACCTTTCTGATGCTATCCTTCTTTCTGACATCCTTAGAGTTAACACCGAGATCACCAAGGCTCCACTTTCTGCTTCTATGATCAAGAGATACGATGAGCACCACCAGGATCTTACCCTTTTGAAGGCTCTTGTTAGACAGCAGCTTCCAGAGAAGTACAAGGAAATCTTCTTCGATCAGTCTAAGAACGGATACGCTGGATACATCGATGGAGGAGCTTCTCAGGAGGAGTTCTACAAGTTCATCAAGCCAATCCTTGAGAAGATGGATGGAACCGAGGAGCTTCTTGTTAAGTTGAACAGAGAGGATCTTCTTAGAAAGCAGAGAACCTTCGATAACGGATCTATCCCACACCAGATCCACCTTGGAGAGCTTCACGCTATCCTTCGTAGACAGGAGGATTTCTACCCATTCTTGAAGGATAACAGAGAGAAGATCGAGAAGATCCTTACCTTCAGAATCCCATACTACGTTGGACCACTTGCTAGAGGAAACTCTCGTTTCGCTTGGATGACCAGAAAGTCTGAGGAGACCATCACCCCTTGGAACTTCGAGGAGgtaagtttctgcttctacctttgatatatatataataattatcattaattagtagtaatataatatttcaaatatttttttcaaaataaaagaatgtagtatatagcaattgcttttctgtagtttataagtgtgtatattttaatttataacttttctaatatatgaccaaaatttgttgatgtgcagGTTGTTGATAAGGGAGCTTCTGCTCAGTCTTTCATCGAGAGAATGACCAACTTCGATAAGAACCTTCCAAACGAGAAGGTTCTTCCAAAGCACTCTCTTCTTTACGAGTACTTCACCGTTTACAACGAGCTTACCAAGGTTAAGTACGTTACCGAGGGAATGAGAAAGCCAGCTTTCCTTTCTGGAGAGCAGAAGAAGGCTATCGTTGATCTTCTTTTCAAGACCAACAGAAAGGTTACCGTTAAGCAGTTGAAGGAGGATTACTTCAAGAAGATCGAGTGCTTCGATTCTGTTGAAATCTCTGGAGTTGAGGATAGATTCAACGCTTCTCTTGGAACCTACCACGATCTTTTGAAGATCATCAAGGATAAGGATTTCCTTGATAACGAGGAGAACGAGGACATCCTTGAGGACATCGTTCTTACCCTTACCCTTTTCGAGGATAGAGAGATGATCGAGGAGAGACTCAAGACCTACGCTCACCTTTTCGATGATAAGGTTATGAAGCAGTTGAAGAGAAGAAGATACACCGGATGGGGTAGACTTTCTCGTAAGTTGATCAACGGAATCAGAGATAAGCAGTCTGGAAAGACCATCCTTGATTTCTTGAAGTCTGATGGATTCGCTAACAGAAACTTCATGCAGCTTATCCACGATGATTCTCTTACCTTCAAGGAGGACATCCAGAAGGCTCAGGTTTCTGGACAGGGAGATTCTCTTCACGAGCACATCGCTAACCTTGCTGGATCTCCAGCTATCAAGAAGGGAATCCTTCAGACCGTTAAGGTTGTTGATGAGCTTGTTAAGGTTATGGGTAGACACAAGCCAGAGAACATCGTTATCGAGATGGCTAGAGAGAACCAGACCACCCAGAAGGGACAGAAGAACTCTCGTGAGAGAATGAAGAGAATCGAGGAGGGAATCAAGGAGCTTGGATCTCAAATCTTGAAGGAGCACCCAGTTGAGAACACCCAGCTTCAGAACGAGAAGTTGTACCTTTACTACCTTCAGAACGGAAGAGATATGTACGTTGATCAGGAGCTTGACATCAACAGACTTTCTGATTACGATGTTGATCACATCGTTCCACAGTCTTTCTTGAAGGATGATTCTATCGATAACAAGGTTCTTACCCGTTCTGATAAGAACAGAGGAAAGTCTGATAACGTTCCATCTGAGGAGGTTGTTAAGAAGATGAAGAACTACTGGAGACAGCTTCTTAACGCTAAGTTGATCACCCAGAGAAAGTTCGATAACCTTACCAAGGCTGAGAGAGGAGGACTTTCTGAGCTTGATAAGGCTGGATTCATCAAGAGACAGCTTGTTGAGACCAGACAGATCACCAAGCACGTTGCTCAGATCCTTGATTCTCGTATGAACACCAAGTACGATGAGAACGATAAGTTGATCAGAGAGGTTAAGGTTATCACCTTGAAGTCTAAGTTGGTTTCTGATTTCAGAAAGGATTTCCAGTTCTACAAGGTTAGAGAGATCAACAACTACCACCACGCTCACGATGCTTACCTTAACGCTGTTGTTGGAACCGCTCTTATCAAGAAGTACCCAAAGTTGGAGTCTGAGTTCGTTTACGGAGATTACAAGGTTTACGATGTTAGAAAGATGATCGCTAAGTCTGAGCAGGAGATCGGAAAGGCTACCGCTAAGTACTTCTTCTACTCTAACATCATGAACTTCTTCAAGACCGAGATCACCCTTGCTAACGGAGAGATCAGAAAGAGACCACTTATCGAGACCAACGGAGAGACCGGAGAGATCGTTTGGGATAAGGGAAGAGATTTCGCTACCGTTAGAAAGGTTCTTTCTATGCCACAGGTTAACATCGTTAAGAAAACCGAGGTTCAGACCGGAGGATTCTCTAAGGAGTCTATCCTTCCAAAGAGAAACTCTGATAAGTTGATCGCTAGAAAGAAGGATTGGGACCCAAAGAAGTACGGAGGATTCGATTCTCCAACCGTTGCTTACTCTGTTCTTGTTGTTGCTAAGGTTGAGAAGGGAAAGTCTAAGAAGTTGAAGTCTGTTAAGGAGCTTCTTGGAATCACCATCATGGAGCGTTCTTCTTTCGAGAAGAACCCAATCGATTTCCTTGAGGCTAAGGGATACAAGGAGGTTAAGAAGGATCTTATCATCAAGTTGCCAAAGTACTCTCTTTTCGAGCTTGAGAACGGAAGAAAGAGAATGCTTGCTTCTGCTGGAGAGCTTCAGAAGGGAAACGAGCTTGCTCTTCCATCTAAGTACGTTAACTTCCTTTACCTTGCTTCTCACTACGAGAAGTTGAAGGGATCTCCAGAGGATAACGAGCAGAAGCAGCTTTTCGTTGAGCAGCACAAGCACTACCTTGATGAGATCATCGAGCAAATCTCTGAGTTCTCTAAGAGAGTTATCCTTGCTGATGCTAACCTTGATAAGGTTCTTTCTGCTTACAACAAGCACAGAGATAAGCCAATCAGAGAGCAGGCTGAGAACATCATCCACCTTTTCACCCTTACCAACCTTGGTGCTCCAGCTGCTTTCAAGTACTTCGATACCACCATCGATAGAAAAAGATACACCTCTACCAAGGAGGTTCTTGATGCTACCCTTATCCACCAGTCTATCACCGGACTTTACGAGACCAGAATCGATCTTTCTCAGCTTGGAGGAGATAAGAGACCAGCTGCTACCAAGAAGGCTGGACAGGCAAAGAAGAAAAAAACAAGAGACTCCGGGGGCAGCACAAATCTCTCTGATATTATTGAGAAAGAAACTGGAAAGCAGTTAGTCATTCAAGAATCTATCTTGATGCTTCCTGAGGAAGTTGAAGAAGTGATTGGAAACAAACCAGAGAGTGACATACTTGTTCATACCGCTTATGATGAGTCTACTGATGAAAATGTTATGTTACTAACGTCAGATGCTCCTGAATACAAGCCATGGGCACTGGTAATACAAGATTCAAATGGAGAGAACAAGATCAAAATGTTGAGTGGTGGTTCGCCCAAAAAGAAAAGGAAGGTGTAGCTGCAGATCGTTCAAACATTTGGCAATAAAGTTTCTTAAGATTGAATCCTGTTGCCGGTCTTGCGATGATTATCATATATATTTCTGTTGATTACGTTAAGCATGTAATAATTAACATGTAATGCATGACGTTATTTATGAGATGGGTTTTTATGATTAGAGTCCCGCAATTATACATTTAATACGCGATAGAAAACAAAATATAGCGCGCAAACTAGGATAAATTATCGCGCGCGGTGTCATCTATGTTACTAGATCCGATGATAAGCTGTCAAACATGAGAATTCGTAATCATGGTCATAGCTGTTTCCTGTGTGAAATTGTTATCCGCTCACAATTCCACACAACATACGAGCCGGAAGCATAAAGTGTAAAGCCTGGGGTGCCTAATGAGTGAGCTAACTCACATTAATTGCGTTGCGCTCACTGCCCGCTTTCCAGTCGGGAAACCTGTCGTGCCAGCTGCATTAATGAATCGGCCAACGCGCGGGGAGAGGCGGTTTGCGTATTGGGCGCTCTTCCGCTTCCTCGCTCACTGACTCGCTGCGCTCGGTCGTTCGGCTGCGGCGAGCGGTATCAGCTCACTCAAAGGCGGTAATACGGTTATCCACAGAATCAGGGGATAACGCAGGAAAGAACATGTGAGCAAAAGGCCAGCAAAAGGCCAGGAACCGTAAAAAGGCCGCGTTGCTGGCGTTTTTCCATAGGCTCCGCCCCCCTGACGAGCATCACAAAAATCGACGCTCAAGTCAGAGGTGGCGAAACCCGACAGGACTATAAAGATACCAGGCGTTTCCCCCTGGAAGCTCCCTCGTGCGCTCTCCTGTTCCGACCCTGCCGCTTACCGGATACCTGTCCGCCTTTCTCCCTTCGGGAAGCGTGGCGCTTTCTCATAGCTCACGCTGTAGGTATCTCAGTTCGGTGTAGGTCGTTCGCTCCAAGCTGGGCTGTGTGCACGAACCCCCCGTTCAGCCCGACCGCTGCGCCTTATCCGGTAACTATCGTCTTGAGTCCAACCCGGTAAGACACGACTTATCGCCACTGGCAGCAGCCACTGGTAACAGGATTAGCAGAGCGAGGTATGTAGGCGGTGCTACAGAGTTCTTGAAGTGGTGGCCTAACTACGGCTACACTAGAAGGACAGTATTTGGTATCTGCGCTCTGCTGAAGCCAGTTACCTTCGGAAAAAGAGTTGGTAGCTCTTGATCCGGCAAACAAACCACCGCTGGTAGCGGTGGTTTTTTTGTTTGCAAGCAGCAGATTACGCGCAGAAAAAAAGGATCTCAAGAAGATCCTTTGATCTTTTCTACGGGGTCTGACGCTCAGTGGAACGAAAACTCACGTTAAGGGATTTTGGTCATGAGATTATCAAAAAGGATCTTCACCTAGATCCTTTTAAATTAAAAATGAAGTTTTAAATCAATCTAAAGTATATATGAGTAAACTTGGTCTGACAGTTACCAATGCTTAATCAGTGAGGCACCTATCTCAGCGATCTGTCTATTTCGTTCATCCATAGTTGCCTGACTCCCCGTCGTGTAGATAACTACGATACGGGAGGGCTTACCATCTGGCCCCAGTGCTGCAATGATACCGCGAGACCCACGCTCACCGGCTCCAGATTTATCAGCAATAAACCAGCCAGCCGGAAGGGCCGAGCGCAGAAGTGGTCCTGCAACTTTATCCGCCTCCATCCAGTCTATTAATTGTTGCCGGGAAGCTAGAGTAAGTAGTTCGCCAGTTAATAGTTTGCGCAACGTTGTTGCCATTGCTACAGGCATCGTGGTGTCACGCTCGTCGTTTGGTATGGCTTCATTCAGCTCCGGTTCCCAACGATCAAGGCGAGTTACATGATCCCCCATGTTGTGCAAAAAAGCGGTTAGCTCCTTCGGTCCTCCGATCGTTGTCAGAAGTAAGTTGGCCGCAGTGTTATCACTCATGGTTATGGCAGCACTGCATAATTCTCTTACTGTCATGCCATCCGTAAGATGCTTTTCTGTGACTGGTGAGTACTCAACCAAGTCATTCTGAGAATAGTGTATGCGGCGACCGAGTTGCTCTTGCCCGGCGTCAATACGGGATAATACCGCGCCACATAGCAGAACTTTAAAAGTGCTCATCATTGGAAAACGTTCTTCGGGGCGAAAACTCTCAAGGATCTTACCGCTGTTGAGATCCAGTTCGATGTAACCCACTCGTGCACCCAACTGATCTTCAGCATCTTTTACTTTCACCAGCGTTTCTGGGTGAGCAAAAACAGGAAGGCAAAATGCCGCAAAAAAGGGAATAAGGGCGACACGGAAATGTTGAATACTCATACTCTTCCTTTTTCAATATTATTGAAGCATTTATCAGGGTTATTGTCTCATGAGCGGATACATATTTGAATGTATTTAGAAAAATAAACAAATAGGGGTTCCGCGCACATTTCCCCGAAAAGTGCCACCTGACGTCTAAGAAACCATTATTATCATGACATTAACCTATAAAAATAGGCGTATCACGAG

>evo_*Pm*CDA1 (CDA1)

GCCCTTTCGTCTCGCGCGTTTCGGTGATGACGGTGAAAACCTCTGACACATGCAGCTCCCGGAGACGGTCACAGCTTGTCTGTAAGCGGATGCCGGGAGCAGACAAGCCCGTCAGGGCGCGTCAGCGGGTGTTGGCGGGTGTCGGGGCTGGCTTAACTATGCGGCATCAGAGCAGATTGTACTGAGAGTGCACCATATGCGGTGTGAAATACCGCACAGATGCGTAAGGAGAAAATACCGCATCAGGCCTCTAGAAAAAAAGCACCGACTCGGTGCCACTTTTTCAAGTTGATAACGGACTAGCCTTATTTTAACTTGCTATTTCTAGCTCTAAAACCCAGTTTTGCTCCAAGGACCAAGCATACATGTTACTGTTCAATTATATAAACACAGCGAAGCTTCTCTCTGCAGACGATGAGGGAGAATTAGCACATTGAGTTTTTCCAAAGGCATGGGCCTGGACCTAGCTACAGGTTTTTGCTCTTGCTTATATGTTCATTCATGCACATAGAGCCCAACTAAGCCTAGAGTCCAGATTTGCCCCCATATTCAACTTGTACCATTTGGTTATTGAAATTAGTGCCCGTATCGGTTTGTTTGGTTTTGAAGTCTATTGATTCGACTTAACGGTTGTCAATTTGTAAATATGTTAAACTGTTATAAAACGACCATTCGGTTTAACCGTTGAAATTTTACACAAAAGAAACTACGGATCGAGATTCAAAATTGTTCTGCCGGAATTTTGAGATTTCTGTCGACTAGAGGATCCCCGCCATTCGCCATTCAGGCTGCGCAACTGTTGGGAAGGGCGATCGGTGCGGGCCTCTTCGCTATTACGCCAGCTGGCGAAAGGGGGATGTGCTGCAAGGCGATTAAGTTGGGTAACGCCAGGGTTTTCCCAGTCACGACGTTGTAAAACGACGGCCAGTGCCAAGCTCTCGAGAAGCTTACTCCAAGAATATCAAAGATACAGTCTCAGAAGACCAAAGGGCTATTGAGACTTTTCAACAAAGGGTAATATCGGGAAACCTCCTCGGATTCCATTGCCCAGCTATCTGTCACTTCATCAAAAGGACAGTAGAAAAGGAAGGTGGCACCTACAAATGCCATCATTGCGATAAAGGAAAGGCTATCGTTCAAGATGCCTCTGCCGACAGTGGTCCCAAAGATGGACCCCCACCCACAAGGAGCATCGTGGAAAAAGAAGACGTTCCAACCACGTCTTCAAAGCAAGTGGATTGATGTGATATCTCCACTGACGTAAGGGATGACGCACAATCCCACTATCCTTCGCCCCAAGCTTGGGCCCAAGCTTGGGTCGCGCCCCACGGATGGTATAAGAATAAAGGCATTCCGCGTGCAGGATTCACCCGTTCGCCTCTCACCTTTTCGCTGTACTCTCTCGCCACACACACCCCCTCTCCAGCTCCGTTGGAGCTCCGGACAGCAGCAGGCGCGGGGCGGTCACGTAGTAAGCAGCTCTCGGCTCCCTCTCCCCTTGCTCCGTGGATCCATGTCTACTGATGCTGAGTATGTGAGGATTCATGAAAAGCTTGACATTTACACTTTCAAAAAGCAGTTCTCAAACAACAAGAAATCTGTCAGTCATAGATGCTATGTTCTTTTTGAGCTAAAAAGGCGTGGAGAACGGAGGGCGTGCTTTTGGGGTTATGCAGTAAACAAACCTCAATCTGGAACCGAGAGAGGGATACATGCTGAAATTTTCAGTATAAGGAAAGTTGAAGAATATCTGAGAGACAATCCAGGACAATTTACTATCAATTGGTATTCCTCTTGGAGTCCTTGTGCTGATTGTGCAGAAAAAATATTGGAATGGTACAATCAAGAATTGCGAGGAAATGGACATACGTTGAAGATTTGGGTGTGCAAATTATACTATGAAAAAAATGCTCGTAACCAAATTGGTCTTTGGAATTTGAGAGATAATGGTGTTGGACTCAATGTTATGGTGTCTGAACATTATCAATGTTGTAGAAAGATATTTATTCAGTCAAGCCACAACCAGTTGAATGAGAATAGATGGCTTGAGAAAACCCTAAAGCGCGCAGAGAAGAGGAGATCAGAGTTATCGATCATGTTTCAGGTTAAAATTTTACACACAACAAAAAGTCCAGCTGTATCGGGTGGGAGCAGCGGAGGATCATCGGGCAGTGAAACACCGGGGACGTCAGAGAGCGCTACTCCTGAATCCTCAGGTGGGAGTAGTGGTGGTTCTGATAAGAAATACTCTATCGGACTTGCGATCGGAACCAACTCTGTTGGATGGGCTGTTATCACCGATGAGTACAAGGTTCCATCTAAGAAGTTCAAGGTTCTTGGAAACACCGATAGACACTCTATCAAGAAGAACCTTATCGGTGCTCTTCTTTTCGATTCTGGAGAGACCGCTGAGGCTACCAGATTGAAGAGAACCGCTAGAAGAAGATACACCAGAAGAAAGAACAGAATCTGCTACCTTCAGGAAATCTTCTCTAACGAGATGGCTAAGGTTGATGATTCTTTCTTCCACAGACTTGAGGAGTCTTTCCTTGTTGAGGAGGATAAGAAGCACGAGAGACACCCAATCTTCGGAAACATCGTTGATGAGGTTGCTTACCACGAGAAGTACCCAACCATCTACCACCTTAGAAAGAAGTTGGTTGATTCTACCGATAAGGCTGATCTTAGACTTATCTACCTTGCTCTTGCTCACATGATCAAGTTCAGAGGACACTTCCTTATCGAGGGAGACCTTAACCCAGATAACTCTGATGTTGATAAGTTGTTCATCCAGCTTGTTCAGACCTACAACCAGCTTTTCGAGGAGAACCCAATCAACGCTTCTGGAGTTGATGCTAAGGCTATCCTTTCTGCTAGACTTTCTAAGTCTCGTAGACTTGAGAACCTTATCGCTCAGCTTCCAGGAGAGAAGAAGAACGGACTTTTCGGAAACCTTATCGCTCTTTCTCTTGGACTTACCCCAAACTTCAAGTCTAACTTCGATCTTGCTGAGGATGCTAAGTTGCAGCTTTCTAAGGATACCTACGATGATGATCTTGATAACCTTCTTGCTCAGATCGGAGATCAGTACGCTGATCTTTTCCTTGCTGCTAAGAACCTTTCTGATGCTATCCTTCTTTCTGACATCCTTAGAGTTAACACCGAGATCACCAAGGCTCCACTTTCTGCTTCTATGATCAAGAGATACGATGAGCACCACCAGGATCTTACCCTTTTGAAGGCTCTTGTTAGACAGCAGCTTCCAGAGAAGTACAAGGAAATCTTCTTCGATCAGTCTAAGAACGGATACGCTGGATACATCGATGGAGGAGCTTCTCAGGAGGAGTTCTACAAGTTCATCAAGCCAATCCTTGAGAAGATGGATGGAACCGAGGAGCTTCTTGTTAAGTTGAACAGAGAGGATCTTCTTAGAAAGCAGAGAACCTTCGATAACGGATCTATCCCACACCAGATCCACCTTGGAGAGCTTCACGCTATCCTTCGTAGACAGGAGGATTTCTACCCATTCTTGAAGGATAACAGAGAGAAGATCGAGAAGATCCTTACCTTCAGAATCCCATACTACGTTGGACCACTTGCTAGAGGAAACTCTCGTTTCGCTTGGATGACCAGAAAGTCTGAGGAGACCATCACCCCTTGGAACTTCGAGGAGgtaagtttctgcttctacctttgatatatatataataattatcattaattagtagtaatataatatttcaaatatttttttcaaaataaaagaatgtagtatatagcaattgcttttctgtagtttataagtgtgtatattttaatttataacttttctaatatatgaccaaaatttgttgatgtgcagGTTGTTGATAAGGGAGCTTCTGCTCAGTCTTTCATCGAGAGAATGACCAACTTCGATAAGAACCTTCCAAACGAGAAGGTTCTTCCAAAGCACTCTCTTCTTTACGAGTACTTCACCGTTTACAACGAGCTTACCAAGGTTAAGTACGTTACCGAGGGAATGAGAAAGCCAGCTTTCCTTTCTGGAGAGCAGAAGAAGGCTATCGTTGATCTTCTTTTCAAGACCAACAGAAAGGTTACCGTTAAGCAGTTGAAGGAGGATTACTTCAAGAAGATCGAGTGCTTCGATTCTGTTGAAATCTCTGGAGTTGAGGATAGATTCAACGCTTCTCTTGGAACCTACCACGATCTTTTGAAGATCATCAAGGATAAGGATTTCCTTGATAACGAGGAGAACGAGGACATCCTTGAGGACATCGTTCTTACCCTTACCCTTTTCGAGGATAGAGAGATGATCGAGGAGAGACTCAAGACCTACGCTCACCTTTTCGATGATAAGGTTATGAAGCAGTTGAAGAGAAGAAGATACACCGGATGGGGTAGACTTTCTCGTAAGTTGATCAACGGAATCAGAGATAAGCAGTCTGGAAAGACCATCCTTGATTTCTTGAAGTCTGATGGATTCGCTAACAGAAACTTCATGCAGCTTATCCACGATGATTCTCTTACCTTCAAGGAGGACATCCAGAAGGCTCAGGTTTCTGGACAGGGAGATTCTCTTCACGAGCACATCGCTAACCTTGCTGGATCTCCAGCTATCAAGAAGGGAATCCTTCAGACCGTTAAGGTTGTTGATGAGCTTGTTAAGGTTATGGGTAGACACAAGCCAGAGAACATCGTTATCGAGATGGCTAGAGAGAACCAGACCACCCAGAAGGGACAGAAGAACTCTCGTGAGAGAATGAAGAGAATCGAGGAGGGAATCAAGGAGCTTGGATCTCAAATCTTGAAGGAGCACCCAGTTGAGAACACCCAGCTTCAGAACGAGAAGTTGTACCTTTACTACCTTCAGAACGGAAGAGATATGTACGTTGATCAGGAGCTTGACATCAACAGACTTTCTGATTACGATGTTGATCACATCGTTCCACAGTCTTTCTTGAAGGATGATTCTATCGATAACAAGGTTCTTACCCGTTCTGATAAGAACAGAGGAAAGTCTGATAACGTTCCATCTGAGGAGGTTGTTAAGAAGATGAAGAACTACTGGAGACAGCTTCTTAACGCTAAGTTGATCACCCAGAGAAAGTTCGATAACCTTACCAAGGCTGAGAGAGGAGGACTTTCTGAGCTTGATAAGGCTGGATTCATCAAGAGACAGCTTGTTGAGACCAGACAGATCACCAAGCACGTTGCTCAGATCCTTGATTCTCGTATGAACACCAAGTACGATGAGAACGATAAGTTGATCAGAGAGGTTAAGGTTATCACCTTGAAGTCTAAGTTGGTTTCTGATTTCAGAAAGGATTTCCAGTTCTACAAGGTTAGAGAGATCAACAACTACCACCACGCTCACGATGCTTACCTTAACGCTGTTGTTGGAACCGCTCTTATCAAGAAGTACCCAAAGTTGGAGTCTGAGTTCGTTTACGGAGATTACAAGGTTTACGATGTTAGAAAGATGATCGCTAAGTCTGAGCAGGAGATCGGAAAGGCTACCGCTAAGTACTTCTTCTACTCTAACATCATGAACTTCTTCAAGACCGAGATCACCCTTGCTAACGGAGAGATCAGAAAGAGACCACTTATCGAGACCAACGGAGAGACCGGAGAGATCGTTTGGGATAAGGGAAGAGATTTCGCTACCGTTAGAAAGGTTCTTTCTATGCCACAGGTTAACATCGTTAAGAAAACCGAGGTTCAGACCGGAGGATTCTCTAAGGAGTCTATCCTTCCAAAGAGAAACTCTGATAAGTTGATCGCTAGAAAGAAGGATTGGGACCCAAAGAAGTACGGAGGATTCGATTCTCCAACCGTTGCTTACTCTGTTCTTGTTGTTGCTAAGGTTGAGAAGGGAAAGTCTAAGAAGTTGAAGTCTGTTAAGGAGCTTCTTGGAATCACCATCATGGAGCGTTCTTCTTTCGAGAAGAACCCAATCGATTTCCTTGAGGCTAAGGGATACAAGGAGGTTAAGAAGGATCTTATCATCAAGTTGCCAAAGTACTCTCTTTTCGAGCTTGAGAACGGAAGAAAGAGAATGCTTGCTTCTGCTGGAGAGCTTCAGAAGGGAAACGAGCTTGCTCTTCCATCTAAGTACGTTAACTTCCTTTACCTTGCTTCTCACTACGAGAAGTTGAAGGGATCTCCAGAGGATAACGAGCAGAAGCAGCTTTTCGTTGAGCAGCACAAGCACTACCTTGATGAGATCATCGAGCAAATCTCTGAGTTCTCTAAGAGAGTTATCCTTGCTGATGCTAACCTTGATAAGGTTCTTTCTGCTTACAACAAGCACAGAGATAAGCCAATCAGAGAGCAGGCTGAGAACATCATCCACCTTTTCACCCTTACCAACCTTGGTGCTCCAGCTGCTTTCAAGTACTTCGATACCACCATCGATAGAAAAAGATACACCTCTACCAAGGAGGTTCTTGATGCTACCCTTATCCACCAGTCTATCACCGGACTTTACGAGACCAGAATCGATCTTTCTCAGCTTGGAGGAGATAAGAGACCAGCTGCTACCAAGAAGGCTGGACAGGCAAAGAAGAAAAAAACAAGAGACTCCGGGGGCAGCACAAATCTCTCTGATATTATTGAGAAAGAAACTGGAAAGCAGTTAGTCATTCAAGAATCTATCTTGATGCTTCCTGAGGAAGTTGAAGAAGTGATTGGAAACAAACCAGAGAGTGACATACTTGTTCATACCGCTTATGATGAGTCTACTGATGAAAATGTTATGTTACTAACGTCAGATGCTCCTGAATACAAGCCATGGGCACTGGTAATACAAGATTCAAATGGAGAGAACAAGATCAAAATGTTGAGTGGTGGTTCGCCCAAAAAGAAAAGGAAGGTGTAGCTGCAGATCGTTCAAACATTTGGCAATAAAGTTTCTTAAGATTGAATCCTGTTGCCGGTCTTGCGATGATTATCATATATATTTCTGTTGATTACGTTAAGCATGTAATAATTAACATGTAATGCATGACGTTATTTATGAGATGGGTTTTTATGATTAGAGTCCCGCAATTATACATTTAATACGCGATAGAAAACAAAATATAGCGCGCAAACTAGGATAAATTATCGCGCGCGGTGTCATCTATGTTACTAGATCCGATGATAAGCTGTCAAACATGAGAATTCGTAATCATGGTCATAGCTGTTTCCTGTGTGAAATTGTTATCCGCTCACAATTCCACACAACATACGAGCCGGAAGCATAAAGTGTAAAGCCTGGGGTGCCTAATGAGTGAGCTAACTCACATTAATTGCGTTGCGCTCACTGCCCGCTTTCCAGTCGGGAAACCTGTCGTGCCAGCTGCATTAATGAATCGGCCAACGCGCGGGGAGAGGCGGTTTGCGTATTGGGCGCTCTTCCGCTTCCTCGCTCACTGACTCGCTGCGCTCGGTCGTTCGGCTGCGGCGAGCGGTATCAGCTCACTCAAAGGCGGTAATACGGTTATCCACAGAATCAGGGGATAACGCAGGAAAGAACATGTGAGCAAAAGGCCAGCAAAAGGCCAGGAACCGTAAAAAGGCCGCGTTGCTGGCGTTTTTCCATAGGCTCCGCCCCCCTGACGAGCATCACAAAAATCGACGCTCAAGTCAGAGGTGGCGAAACCCGACAGGACTATAAAGATACCAGGCGTTTCCCCCTGGAAGCTCCCTCGTGCGCTCTCCTGTTCCGACCCTGCCGCTTACCGGATACCTGTCCGCCTTTCTCCCTTCGGGAAGCGTGGCGCTTTCTCATAGCTCACGCTGTAGGTATCTCAGTTCGGTGTAGGTCGTTCGCTCCAAGCTGGGCTGTGTGCACGAACCCCCCGTTCAGCCCGACCGCTGCGCCTTATCCGGTAACTATCGTCTTGAGTCCAACCCGGTAAGACACGACTTATCGCCACTGGCAGCAGCCACTGGTAACAGGATTAGCAGAGCGAGGTATGTAGGCGGTGCTACAGAGTTCTTGAAGTGGTGGCCTAACTACGGCTACACTAGAAGGACAGTATTTGGTATCTGCGCTCTGCTGAAGCCAGTTACCTTCGGAAAAAGAGTTGGTAGCTCTTGATCCGGCAAACAAACCACCGCTGGTAGCGGTGGTTTTTTTGTTTGCAAGCAGCAGATTACGCGCAGAAAAAAAGGATCTCAAGAAGATCCTTTGATCTTTTCTACGGGGTCTGACGCTCAGTGGAACGAAAACTCACGTTAAGGGATTTTGGTCATGAGATTATCAAAAAGGATCTTCACCTAGATCCTTTTAAATTAAAAATGAAGTTTTAAATCAATCTAAAGTATATATGAGTAAACTTGGTCTGACAGTTACCAATGCTTAATCAGTGAGGCACCTATCTCAGCGATCTGTCTATTTCGTTCATCCATAGTTGCCTGACTCCCCGTCGTGTAGATAACTACGATACGGGAGGGCTTACCATCTGGCCCCAGTGCTGCAATGATACCGCGAGACCCACGCTCACCGGCTCCAGATTTATCAGCAATAAACCAGCCAGCCGGAAGGGCCGAGCGCAGAAGTGGTCCTGCAACTTTATCCGCCTCCATCCAGTCTATTAATTGTTGCCGGGAAGCTAGAGTAAGTAGTTCGCCAGTTAATAGTTTGCGCAACGTTGTTGCCATTGCTACAGGCATCGTGGTGTCACGCTCGTCGTTTGGTATGGCTTCATTCAGCTCCGGTTCCCAACGATCAAGGCGAGTTACATGATCCCCCATGTTGTGCAAAAAAGCGGTTAGCTCCTTCGGTCCTCCGATCGTTGTCAGAAGTAAGTTGGCCGCAGTGTTATCACTCATGGTTATGGCAGCACTGCATAATTCTCTTACTGTCATGCCATCCGTAAGATGCTTTTCTGTGACTGGTGAGTACTCAACCAAGTCATTCTGAGAATAGTGTATGCGGCGACCGAGTTGCTCTTGCCCGGCGTCAATACGGGATAATACCGCGCCACATAGCAGAACTTTAAAAGTGCTCATCATTGGAAAACGTTCTTCGGGGCGAAAACTCTCAAGGATCTTACCGCTGTTGAGATCCAGTTCGATGTAACCCACTCGTGCACCCAACTGATCTTCAGCATCTTTTACTTTCACCAGCGTTTCTGGGTGAGCAAAAACAGGAAGGCAAAATGCCGCAAAAAAGGGAATAAGGGCGACACGGAAATGTTGAATACTCATACTCTTCCTTTTTCAATATTATTGAAGCATTTATCAGGGTTATTGTCTCATGAGCGGATACATATTTGAATGTATTTAGAAAAATAAACAAATAGGGGTTCCGCGCACATTTCCCCGAAAAGTGCCACCTGACGTCTAAGAAACCATTATTATCATGACATTAACCTATAAAAATAGGCGTATCACGAG

**Indel Detection Amplicon Analysis results (IDAA)**

| A1 | A3A replicate 1 |
| --- | --- |
| A2 | A3A replicate 2 |
| A3 | A3A replicate 3 |
| A4 | A3A replicate 4 |
| R1 | rA1 replicate 1 |
| R2 | rA1 replicate 2 |
| R3 | rA1 replicate 3 |
| R4 | rA1 replicate 4 |
| C1 | CDA1 replicate 1 |
| C2 | CDA1 replicate 2 |
| C3 | CDA1 replicate 3 |
| C4 | CDA1 replicate 4 |
| GFP-1 | GFP replicate 1 |
| nP16-1 | *Sp*Cas9/*St*U6-1::sgRNA1 nickase replicate 1 |
| nP16-2 | *Sp*Cas9/*St*U6-1::sgRNA1 nickase replicate 2 |

**
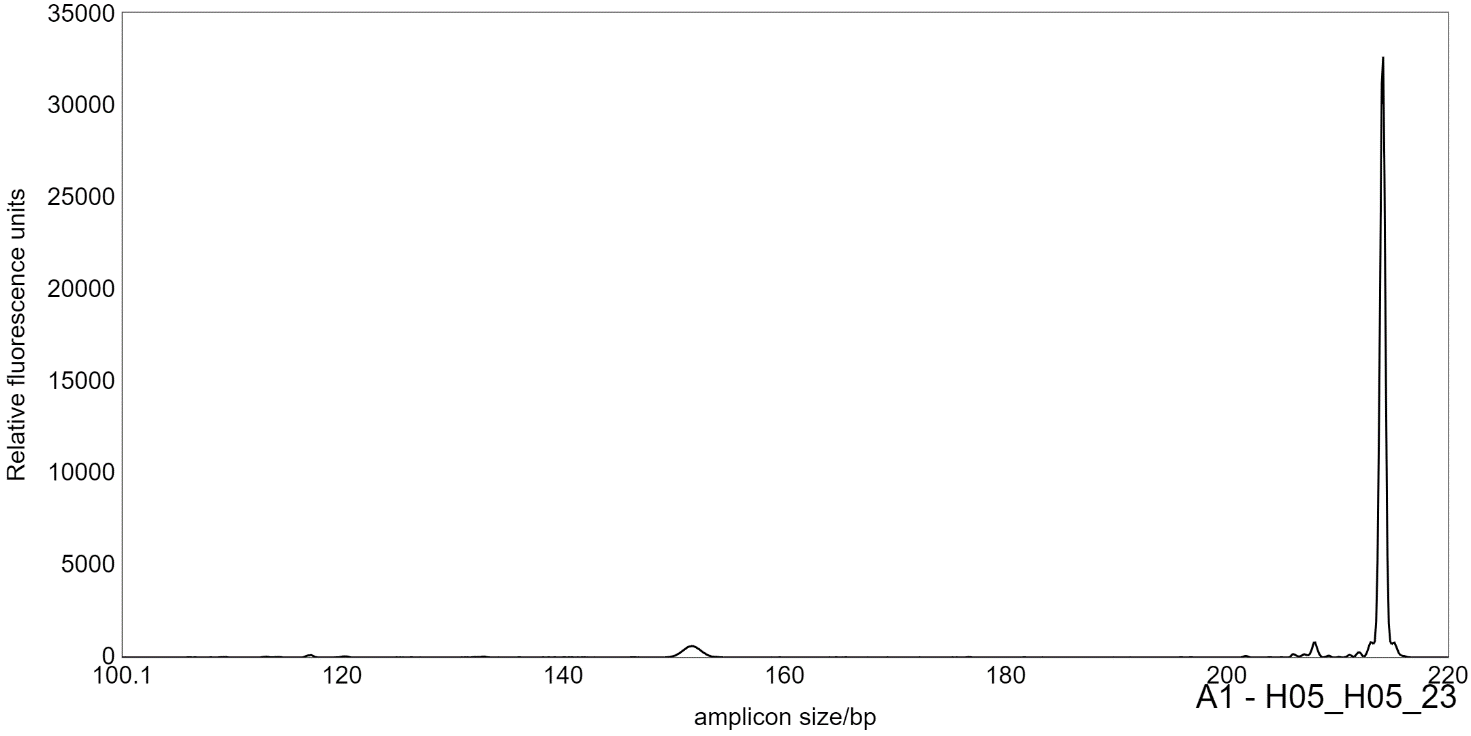
**

**
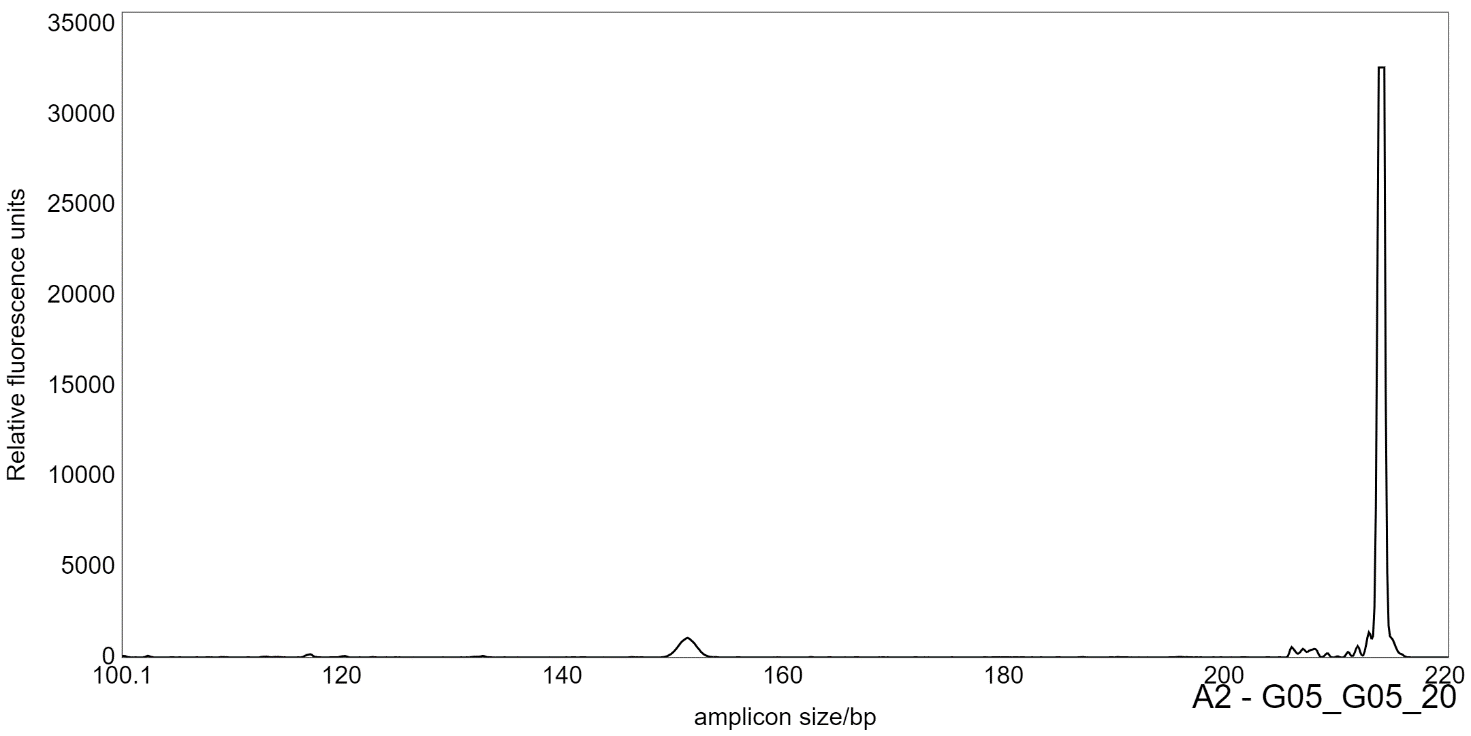
**

**
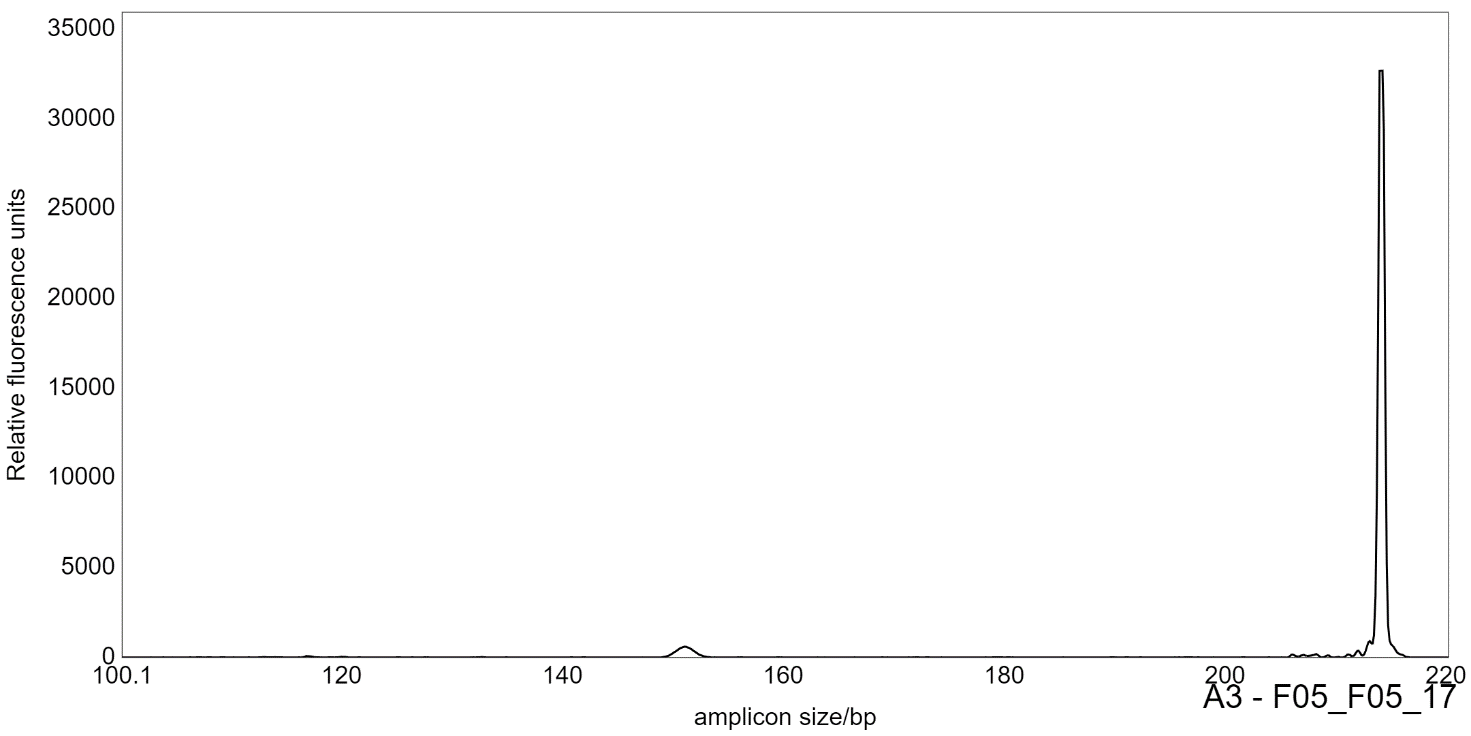
**

**
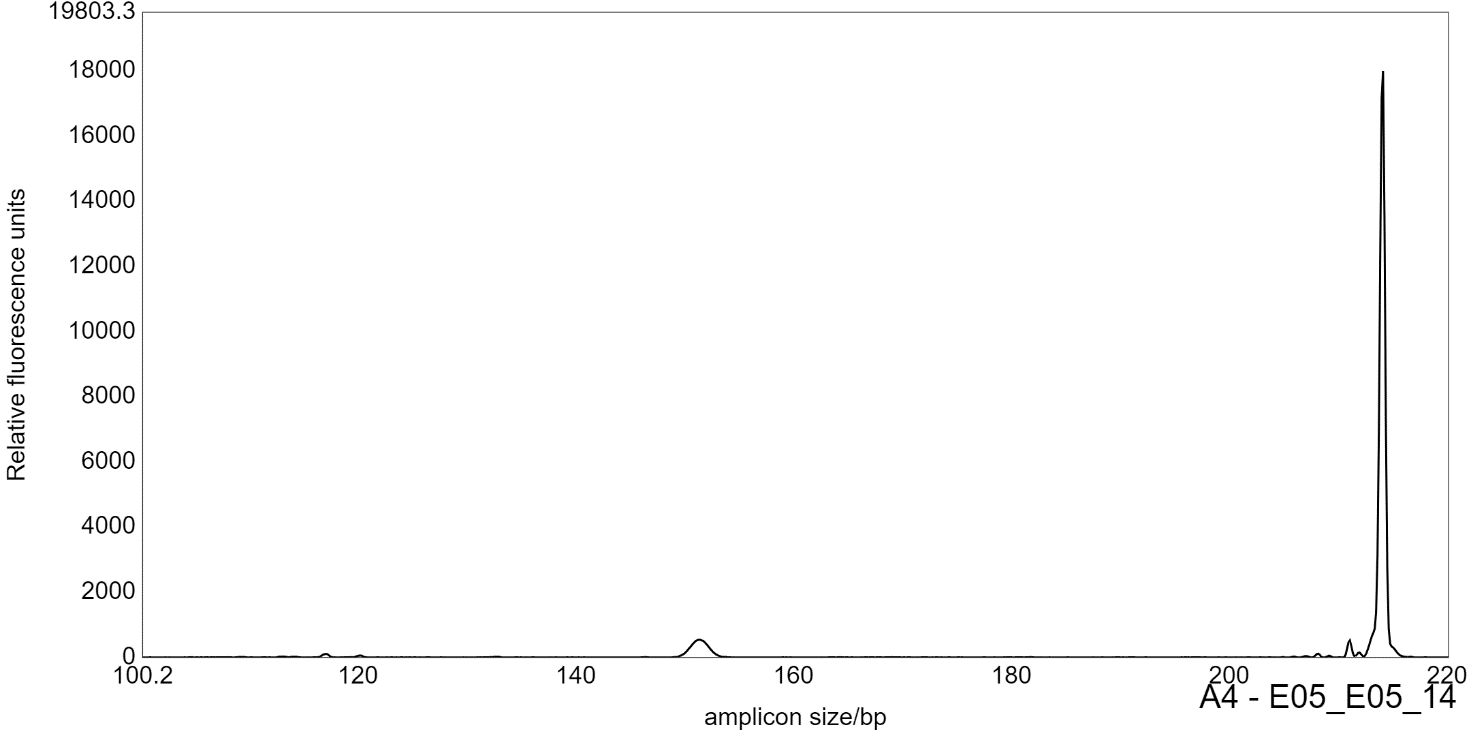
**

**
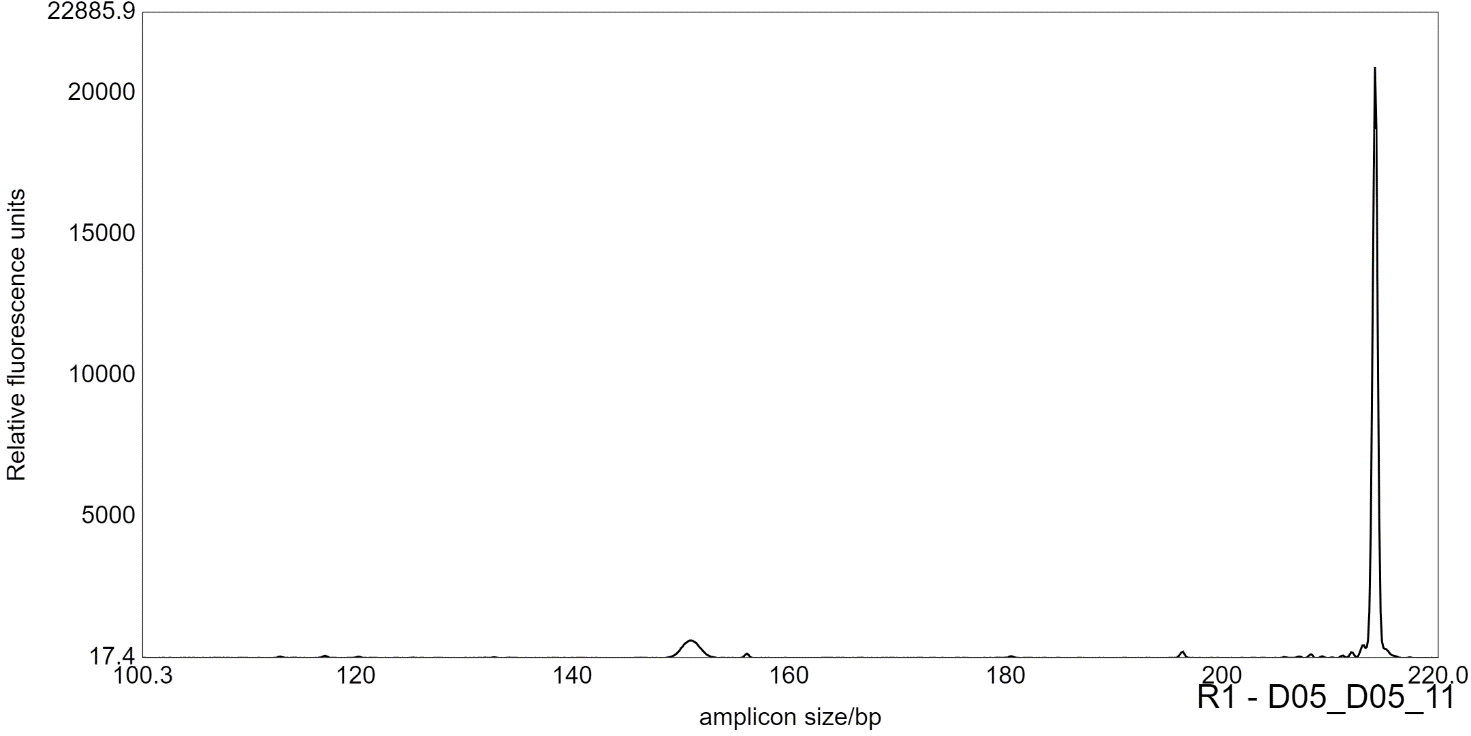
**

**
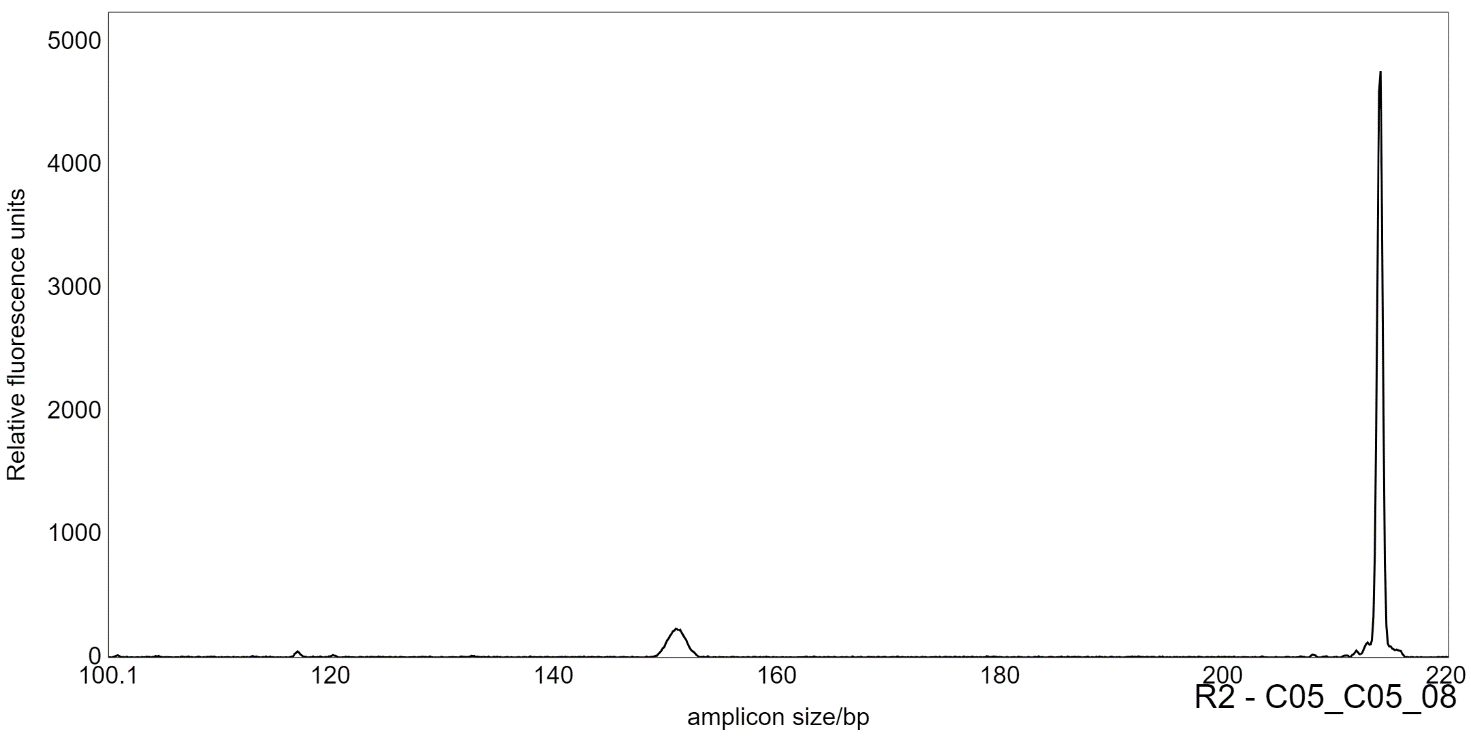
**

**
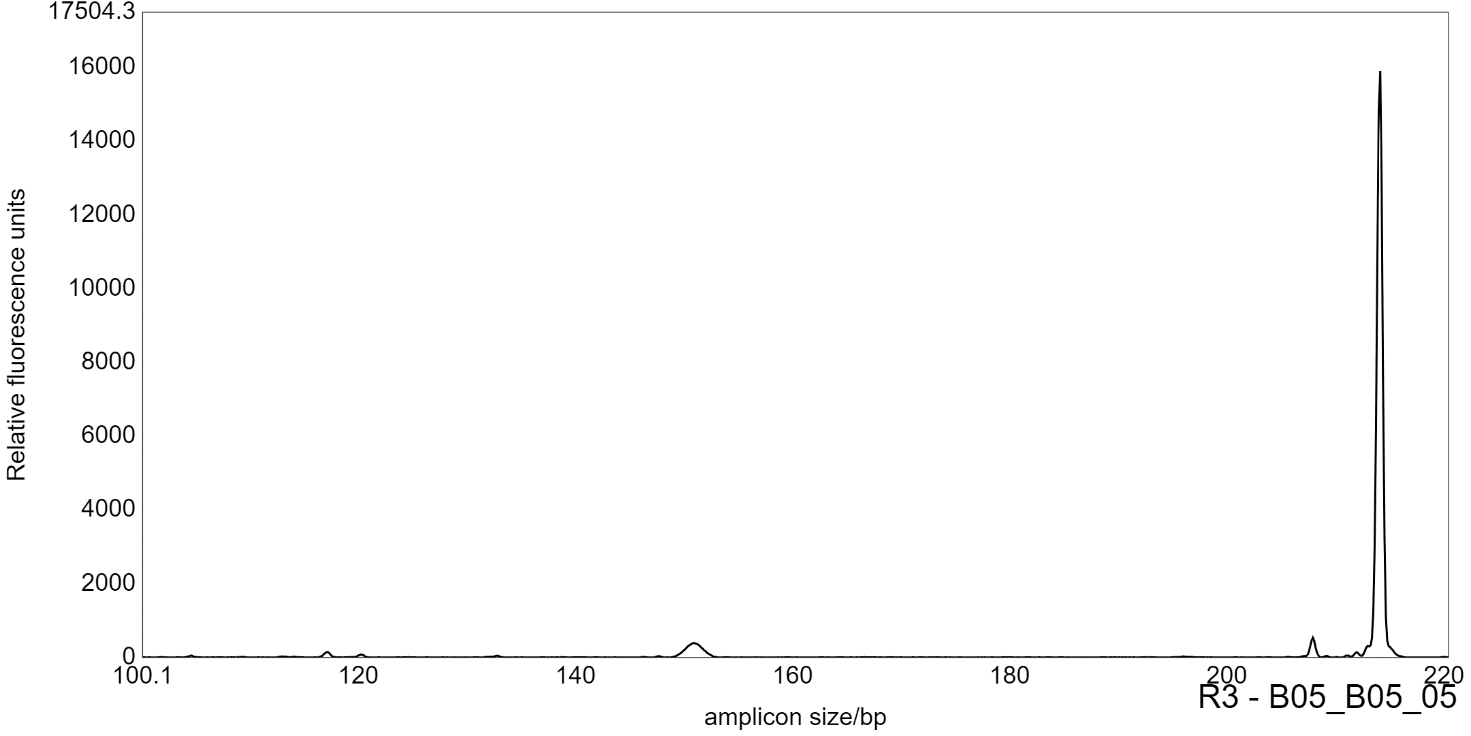
**

**
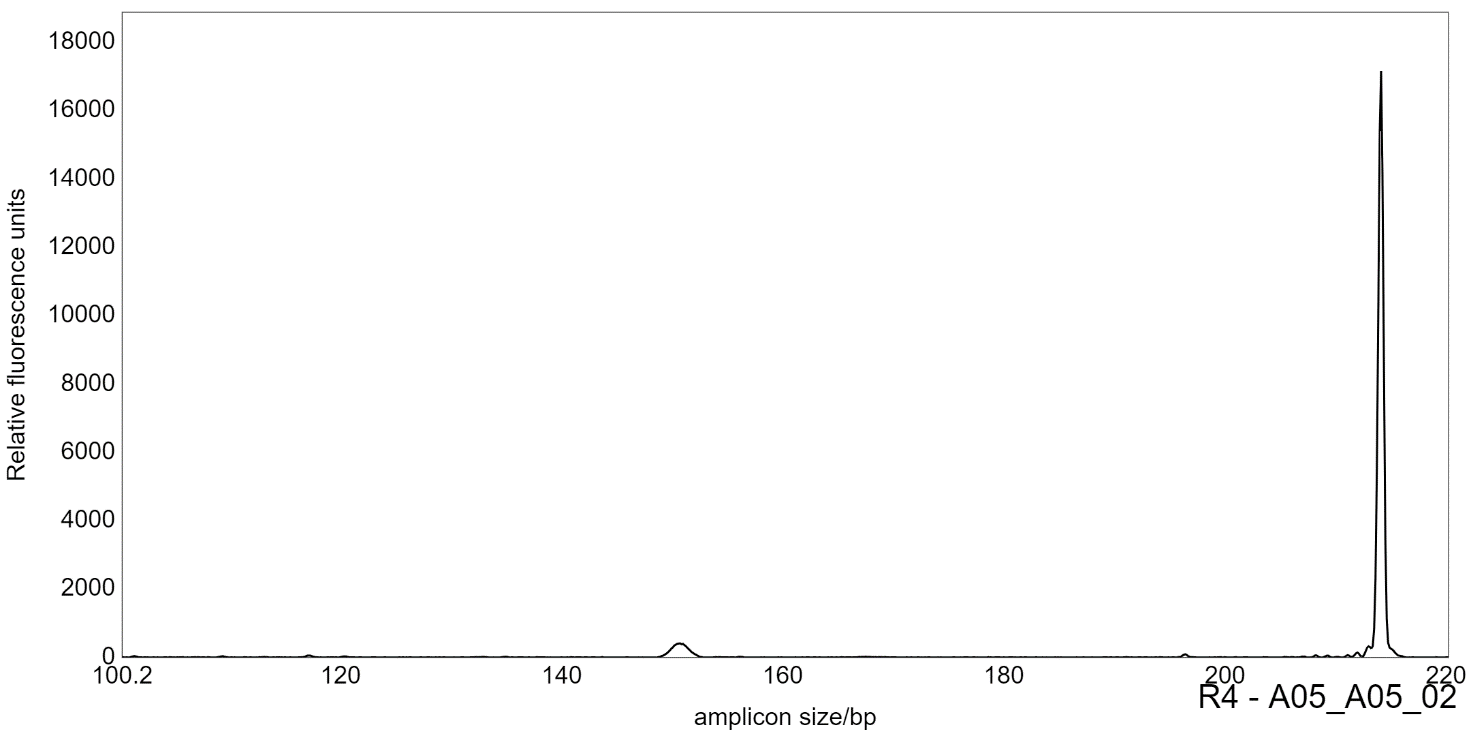
**

**
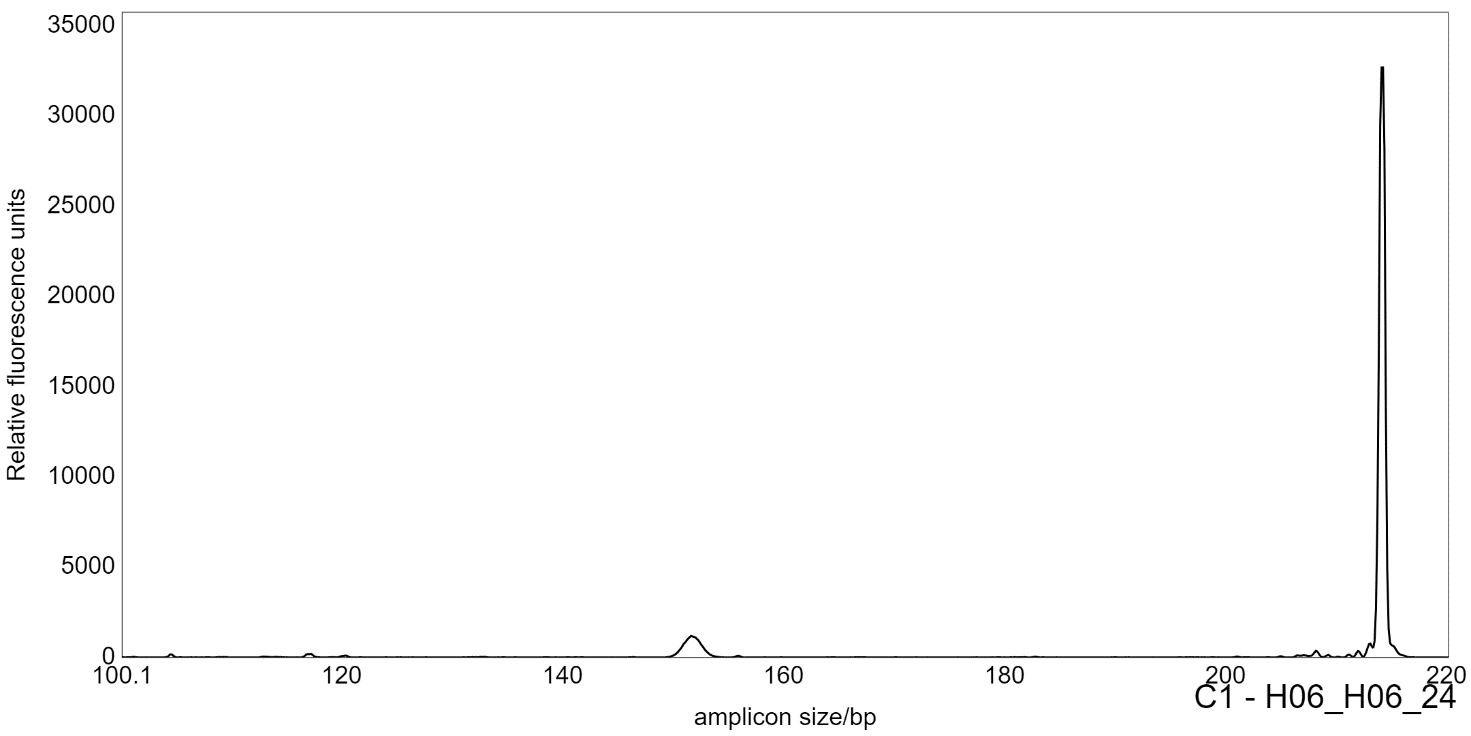
**

**
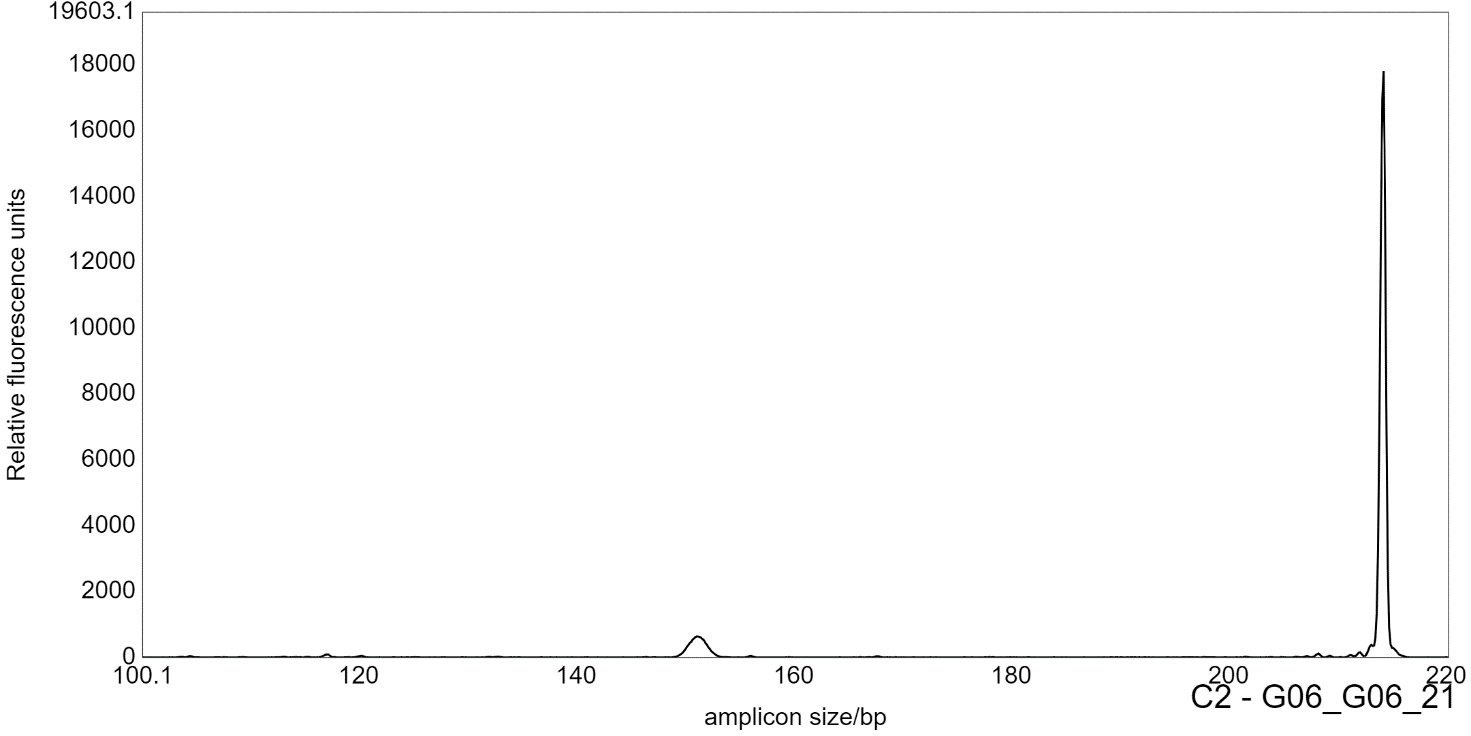
**

**
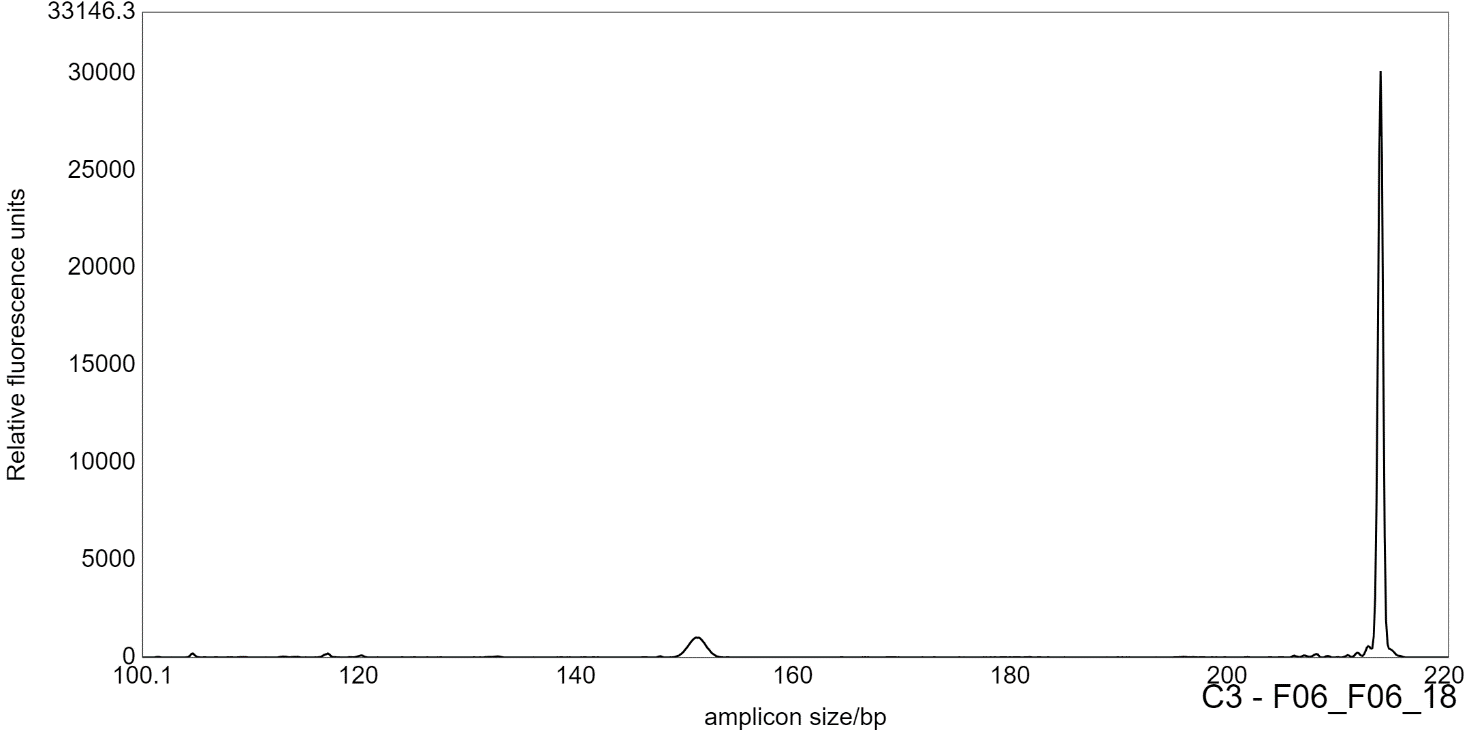
**

**
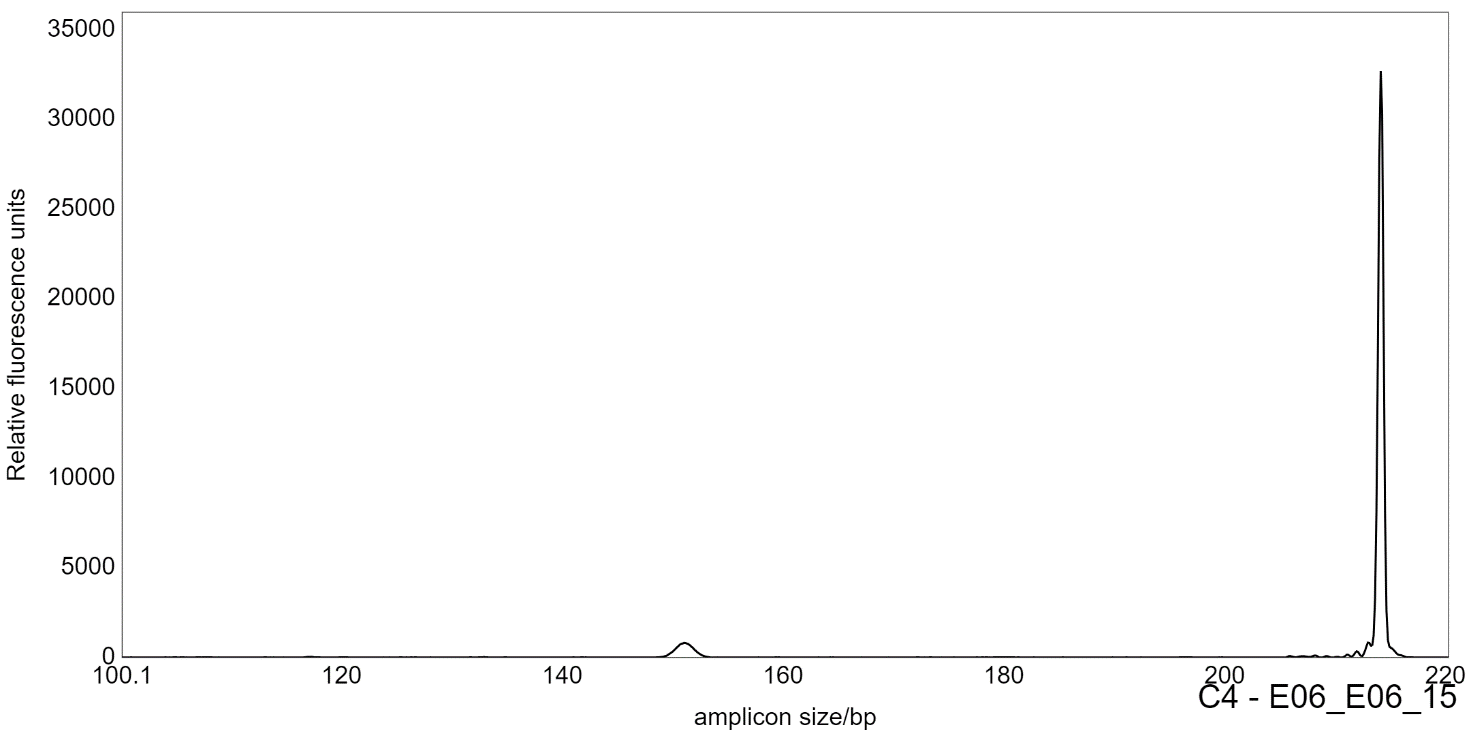
**

**
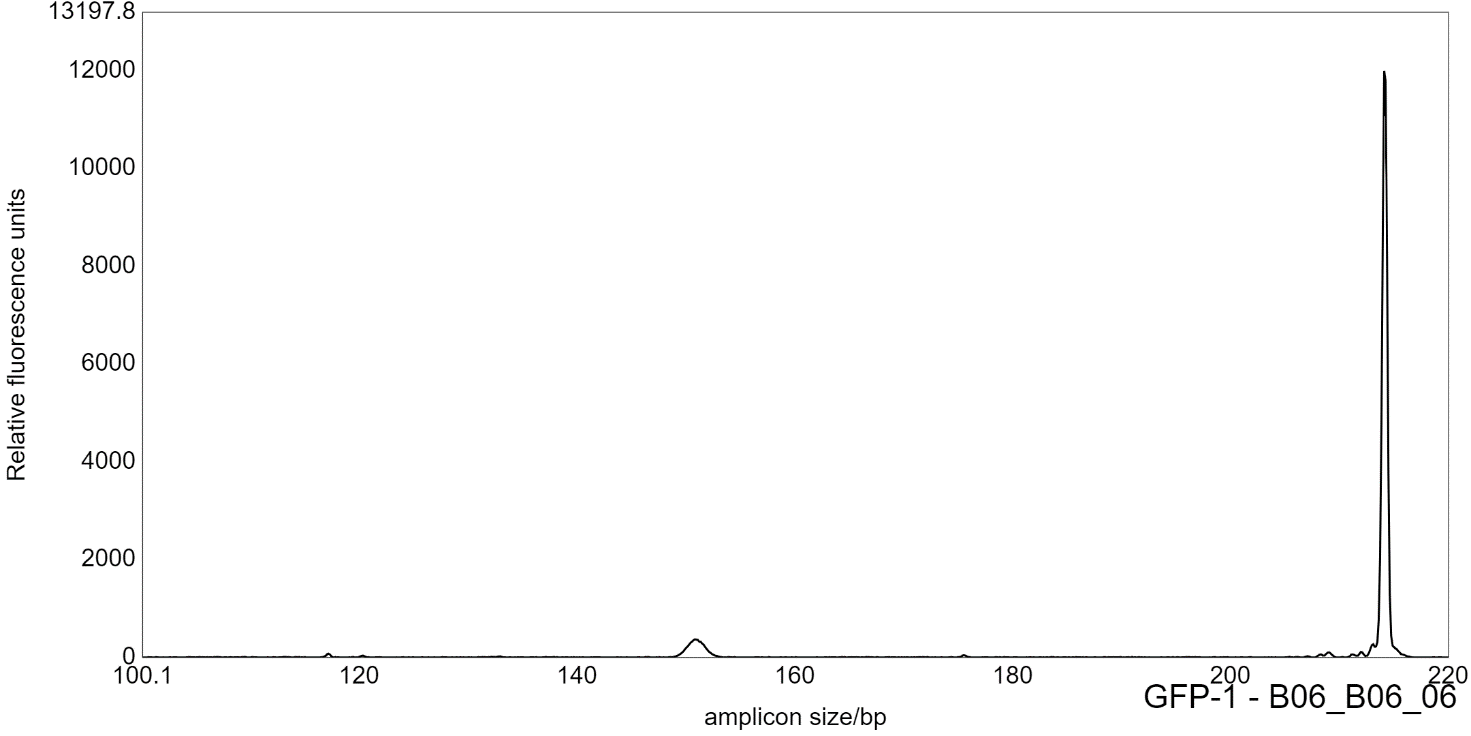
**

**
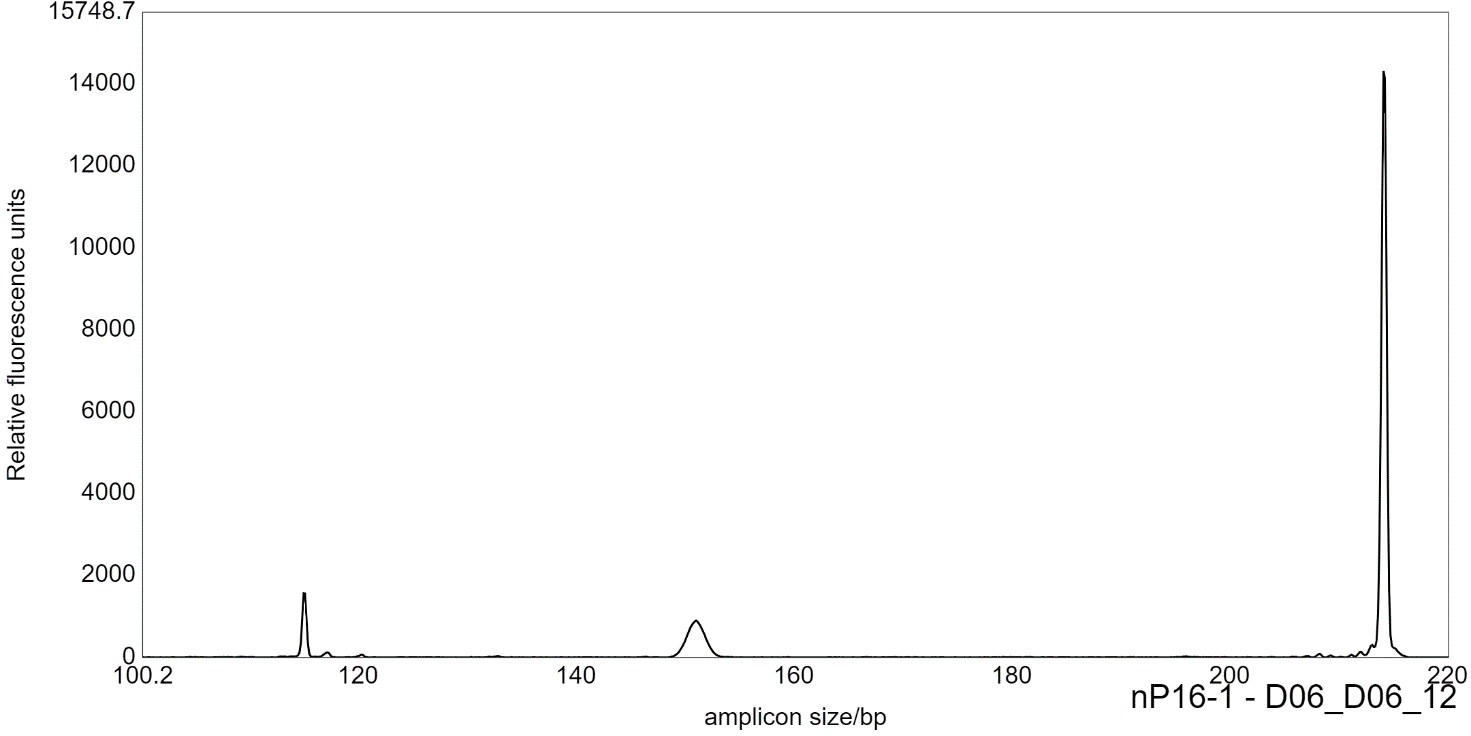
**

**
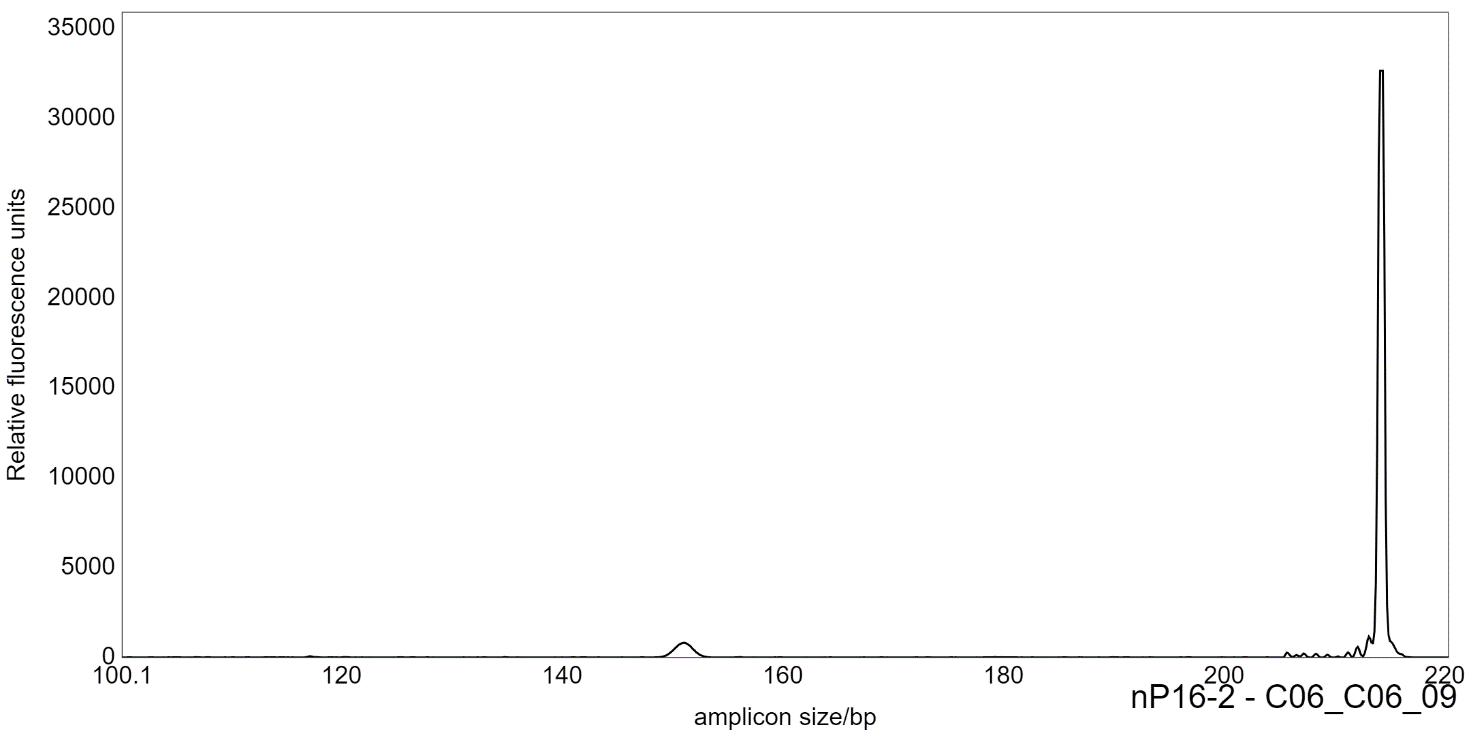
**

**Restriction digestion**

**BsrI digestion**

**
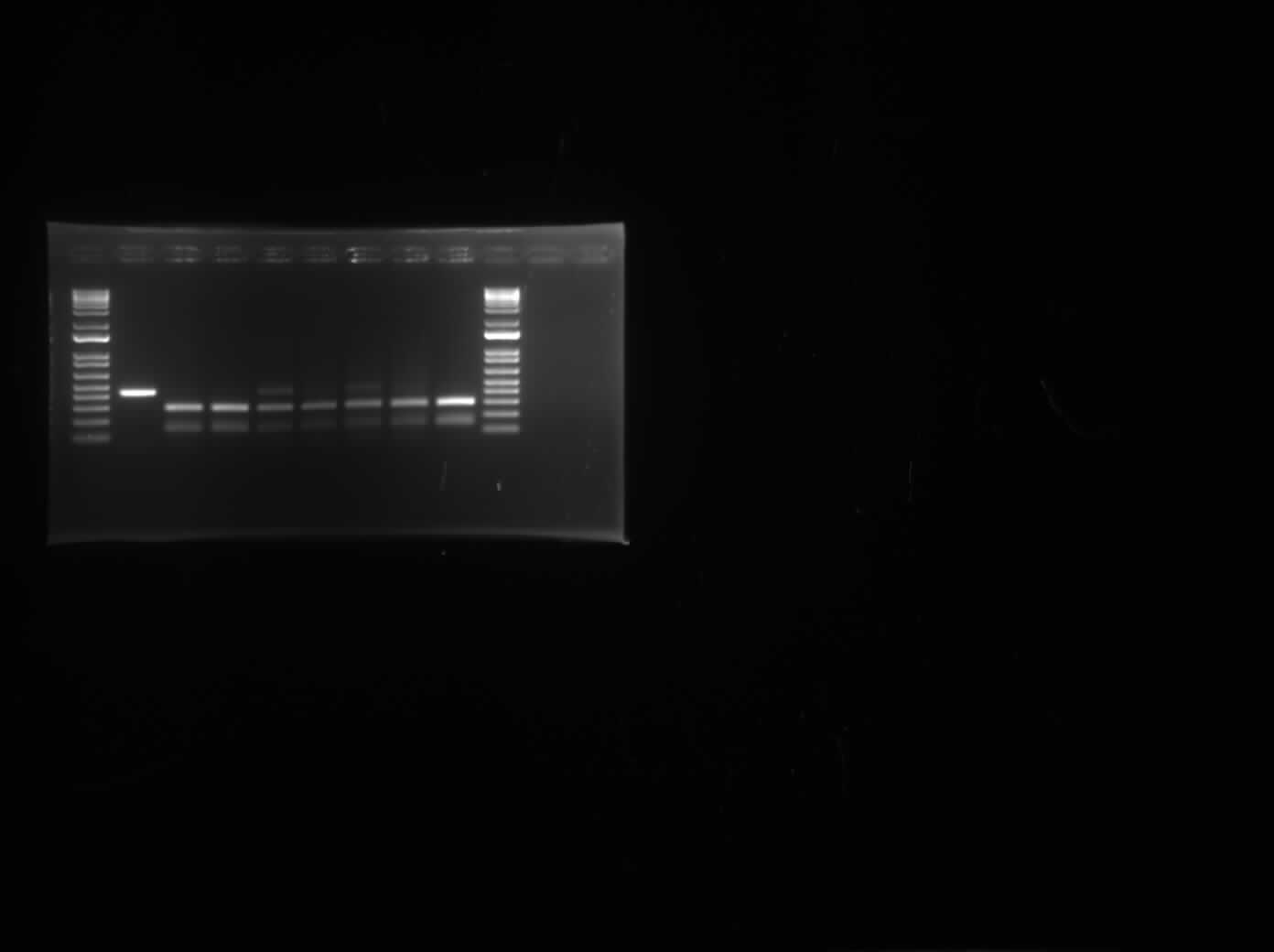
**

Figure A1. BsrI digestion of the 429 bp GBSS1 fragment amplified with primers 384 and 472 from gDNA extracted from transfected protoplasts. Lane 1: Ladder GeneRuler^TM^ 1 kb Plus DNA Ladder from ThermoFisher Scientific^TM^, Lane 2: Undigested GBSS1 fragment with no addition of BsrI enzyme, Lane 3: Protoplasts transformed with GFP, Lane 4: Protoplasts transformed with SpCas9/StU6-1::sgRNA1 nickase, Lane 5: protoplasts transformed with SpCas9/StU6-1::sgRNA1, Lane 6: Protoplasts transformed with A3A BE construct, Lane 7: Protoplasts transformed with rA1 BE construct, Lane 8: Protoplasts transformed with CDA1 BE construct.

**StyI digestion**

**
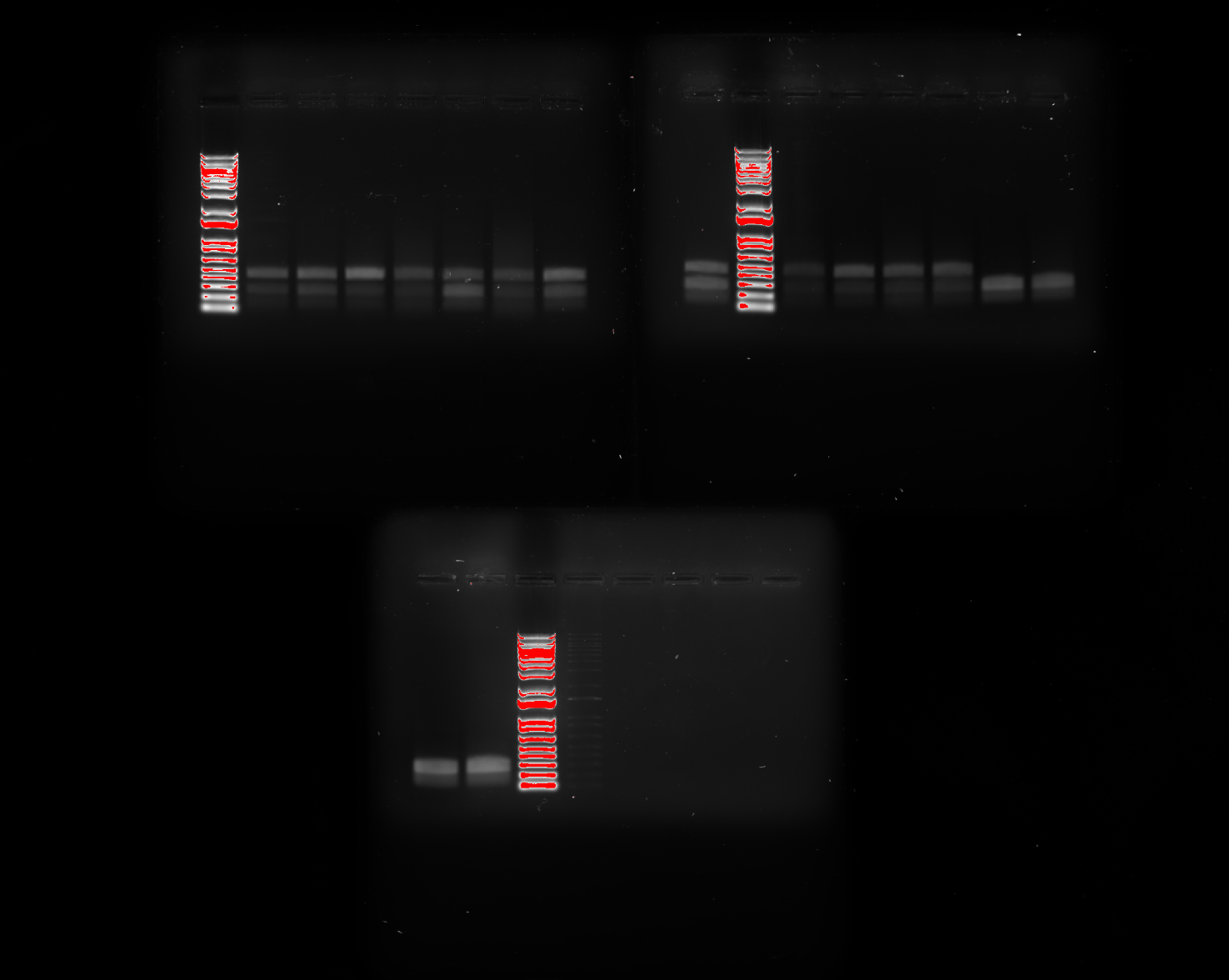
**

Figure A2. StyI digestion of the 429 bp GBSS1 fragment amplified with primers 384 and 472 from gDNA extracted from transfected protoplasts. Lane 1: Ladder GeneRuler^TM^ 1 kb Plus DNA Ladder from ThermoFisher Scientific^TM^. Lane 2: Protoplasts transformed with A3A BE construct replicate 1, Lane 3: Protoplasts transformed with A3A BE construct replicate 2, Lane 4: Protoplasts transformed with A3A BE construct replicate 3, Lane 5: Protoplasts transformed with A3A BE construct replicate 4, Lane 6: Protoplasts transformed with rA1 BE construct replicate 1, Lane 7: Protoplasts transformed with rA1 BE construct replicate 2, Lane 8: Protoplasts transformed with rA1 BE construct replicate 3.


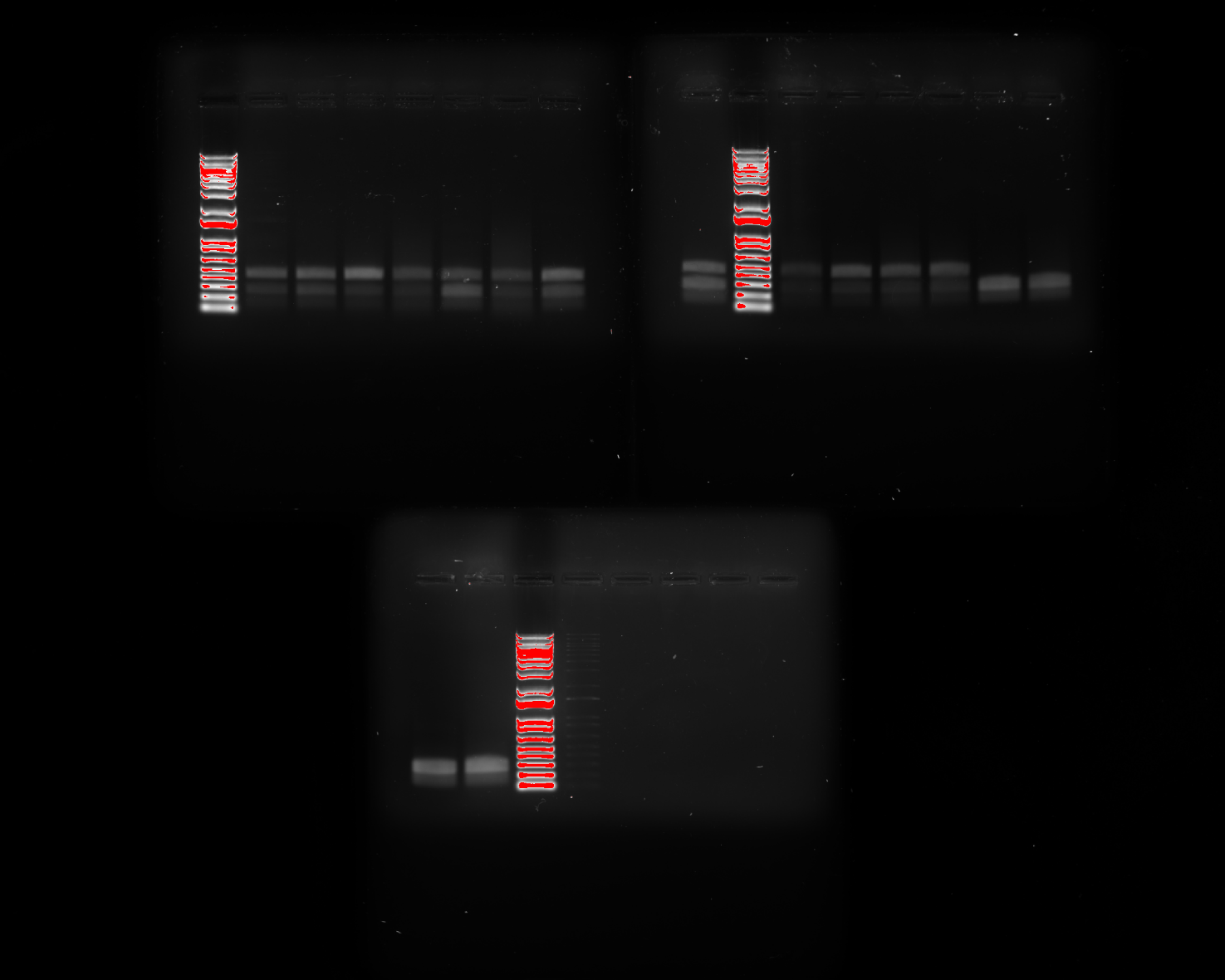


Figure A3. StyI digestion of the 429 bp GBSS1 fragment amplified with primers 384 and 472 from gDNA extracted from transfected protoplasts. Lane 1: Protoplasts transformed with rA1 BE construct replicate 4., Lane 2: Ladder GeneRuler^TM^ 1 kb Plus DNA Ladder from ThermoFisher Scientific^TM^, Lane 3: Protoplasts transformed with CDA1 BE construct replicate 1, Lane 4: Protoplasts transformed with CDA1 BE construct replicate 2, Lane 5: Protoplasts transformed with CDA1 BE construct replicate 3, Lane 6: Protoplasts transformed with CDA1 BE construct replicate 4, Lane 7: Protoplasts transformed with SpCas9/StU6-1::sgRNA1 nickase replicate 1, Lane 8: Protoplasts transformed with *Sp*Cas9/StU6-1::sgRNA1 nickase replicate 2.


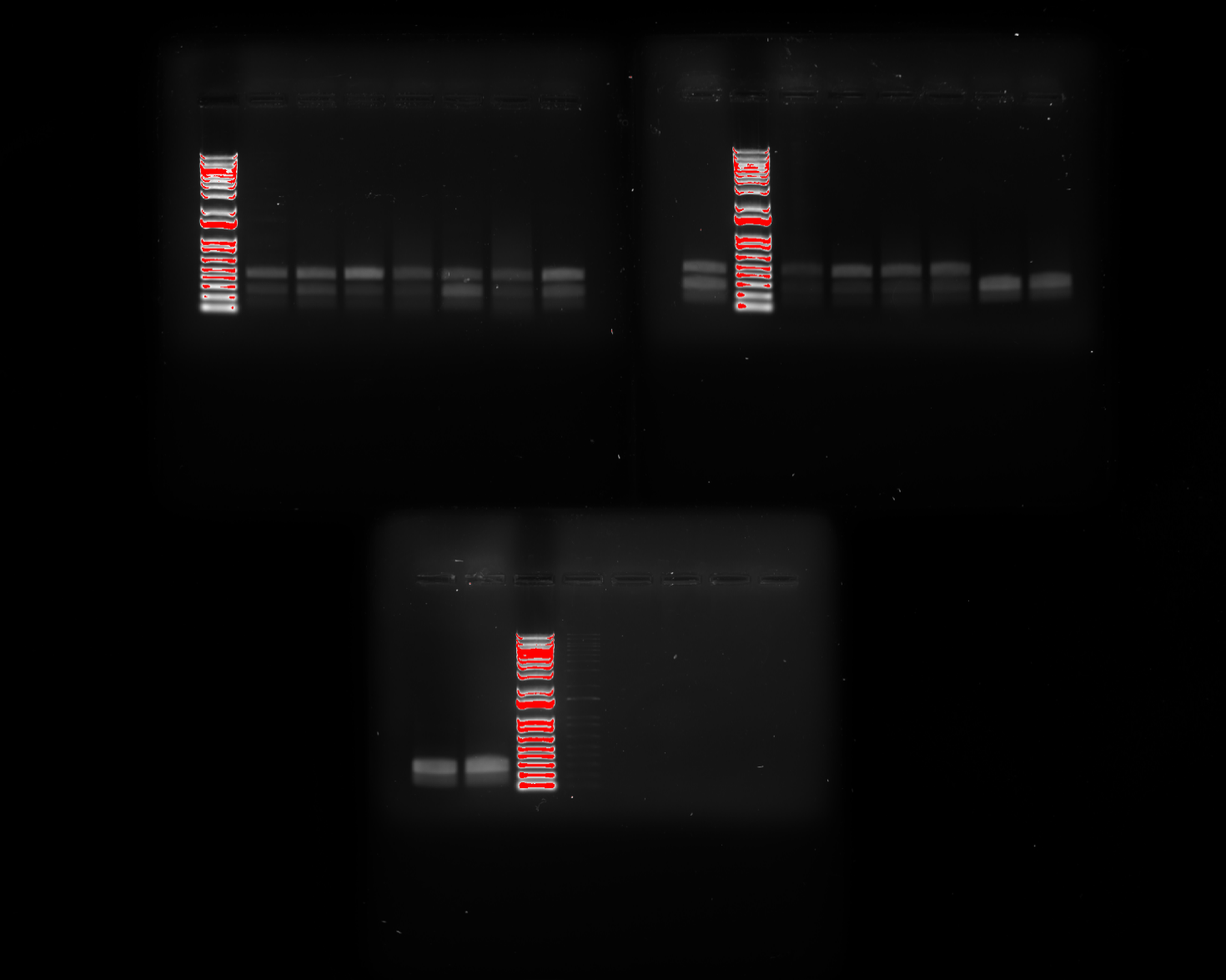


Figure A4. StyI digestion of the 429 bp GBSS1 fragment amplified with primers 384 and 472 from gDNA extracted from transfected protoplasts. Lane 1: Protoplasts transformed with GFP replicate 1, Lane 2: Protoplasts transformed with GFP replicate 2, Lane 3: Ladder GeneRuler^TM^ 1 kb Plus DNA Ladder from ThermoFisher Scientific^TM^.

**EditR results**

| A1 | A3A replicate 1 |
| --- | --- |
| A2 | A3A replicate 2 |
| A3 | A3A replicate 3 |
| A4 | A3A replicate 4 |
| R1 | rA1 replicate 1 |
| R2 | rA1 replicate 2 |
| R3 | rA1 replicate 3 |
| R4 | rA1 replicate 4 |
| C1 | CDA1 replicate 1 |
| C2 | CDA1 replicate 2 |
| C3 | CDA1 replicate 3 |
| C4 | CDA1 replicate 4 |
| GFP-1 | GFP replicate 1 |
| GFP-2 | GFP replicate 2 |
| nP16-1 | *Sp*Cas9/StU6-1::sgRNA1 nickase replicate 1 |
| nP16-2 | *Sp*Cas9/StU6-1::sgRNA1 nickase replicate 2 |

**A1**

**
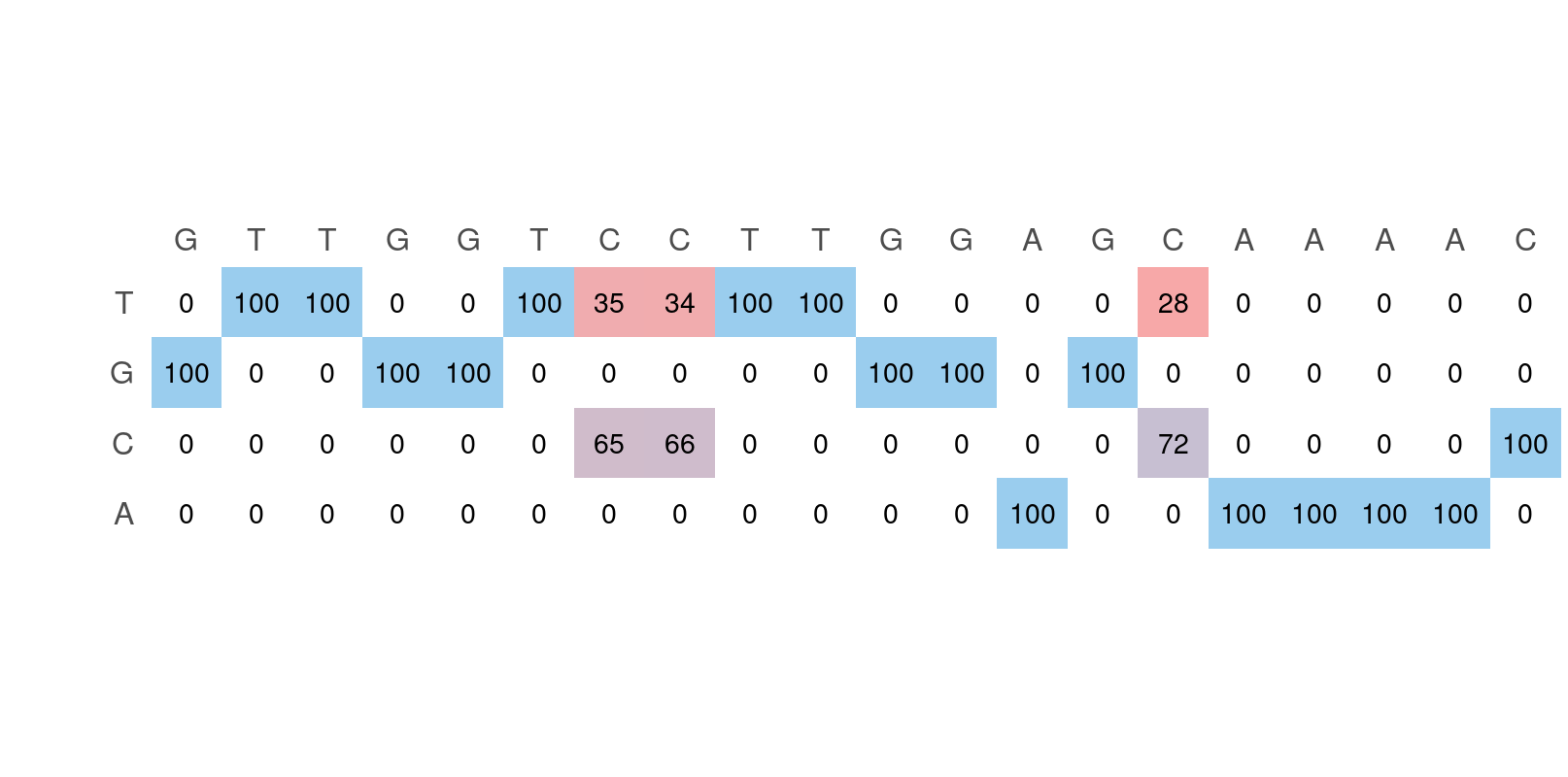
**

**A2**

**
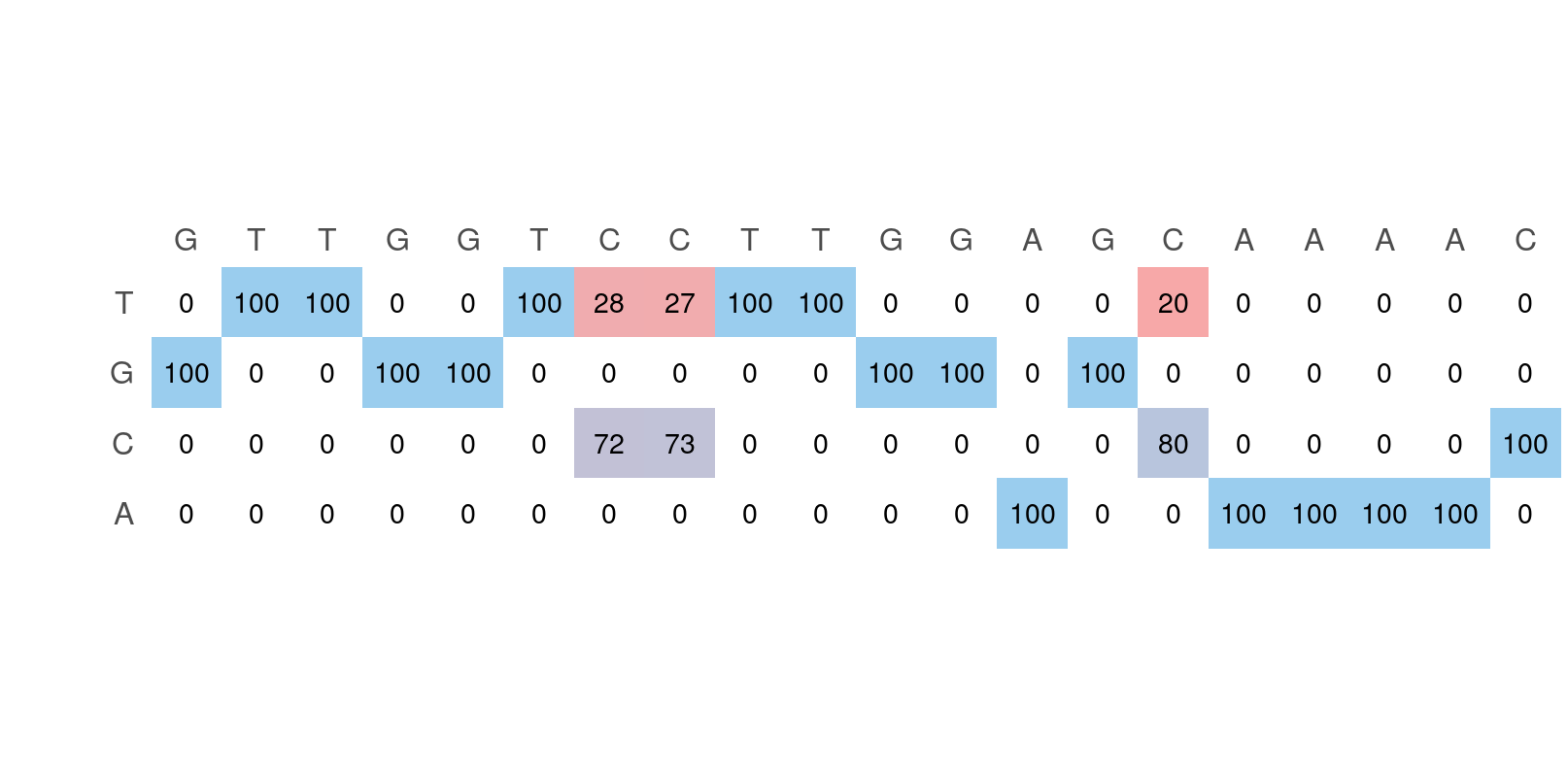
**

**A3**

**
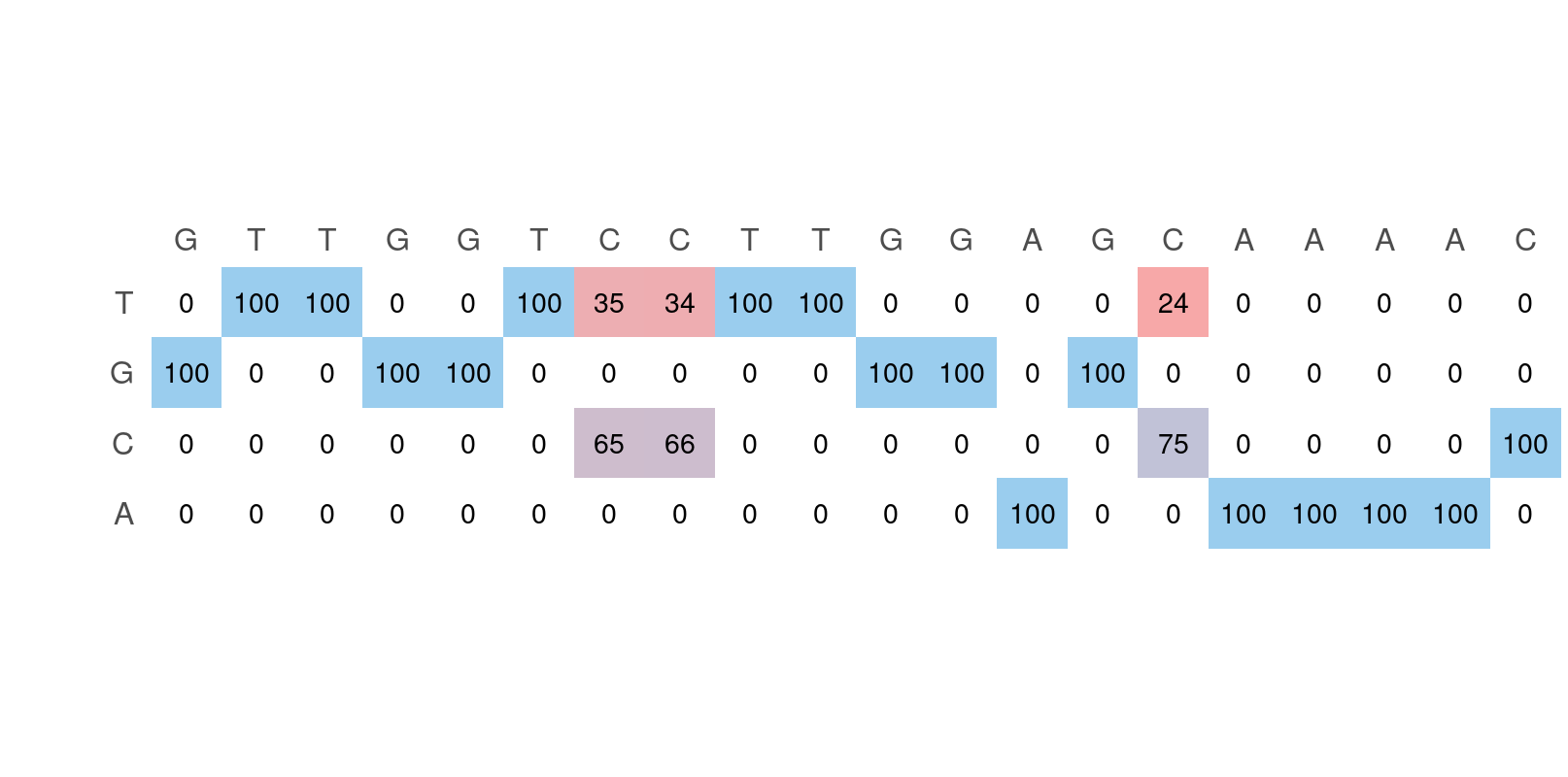
**

**A4**

**
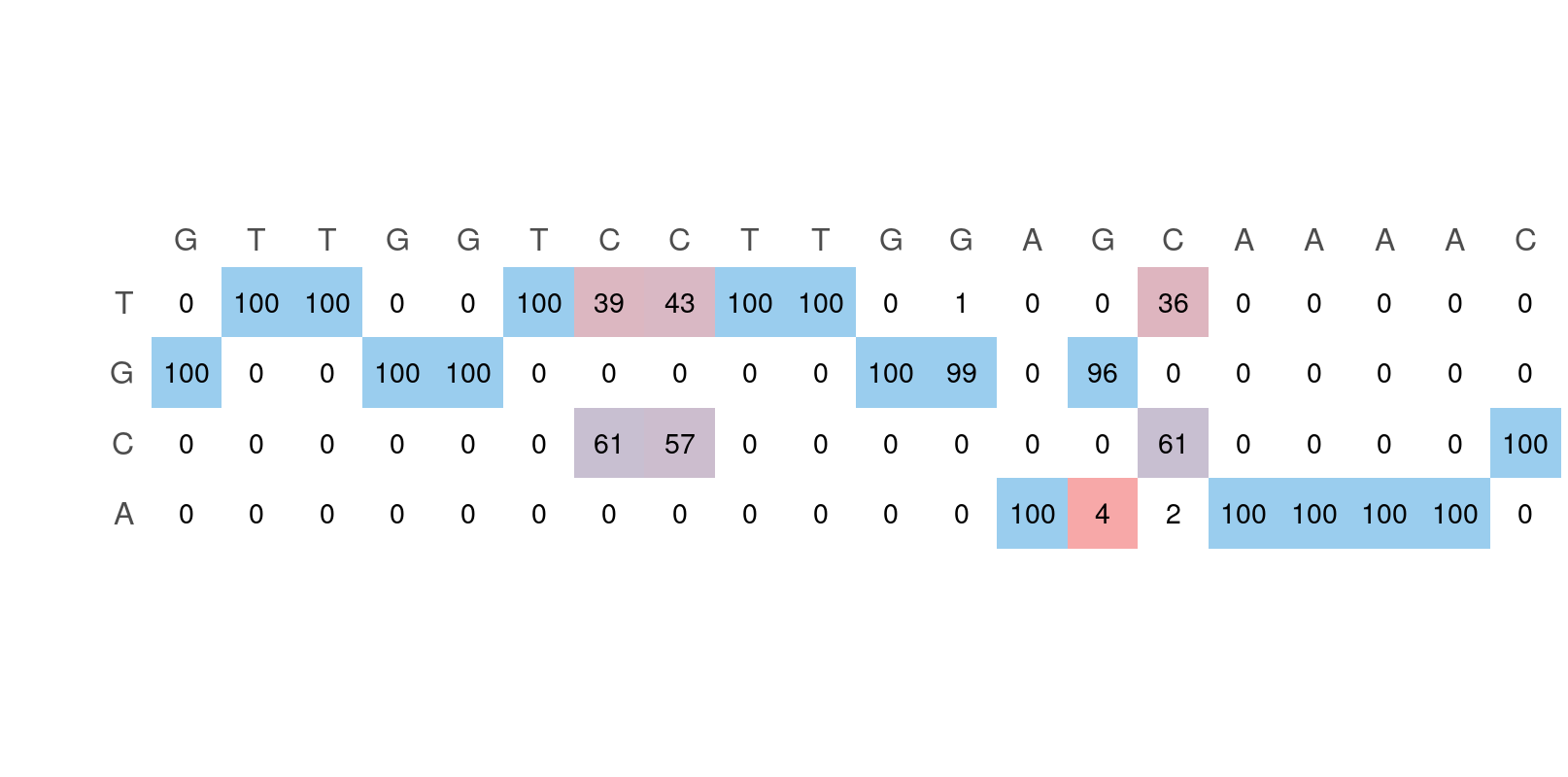
**

**R1**

**
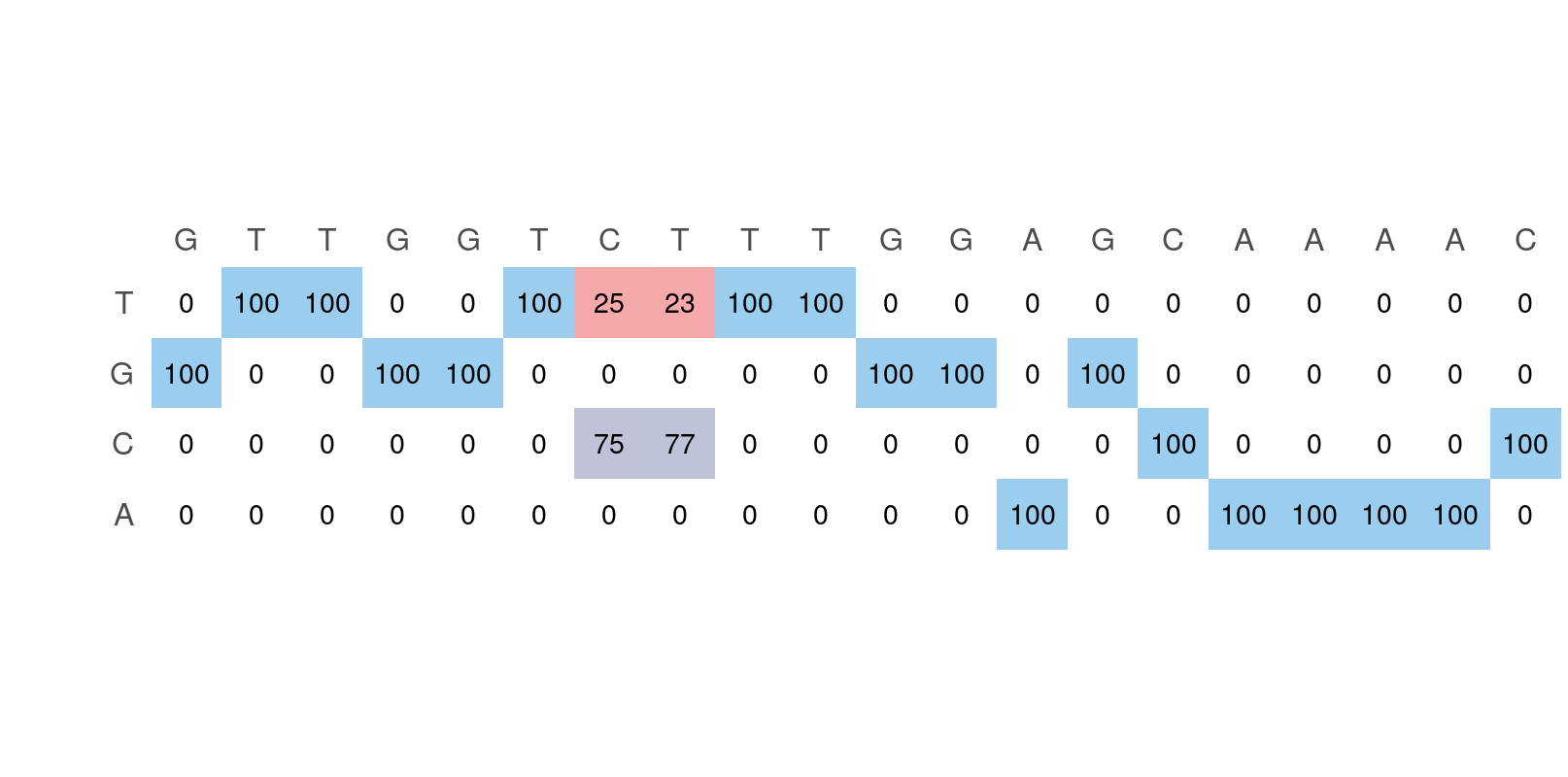
**

**R2**

**
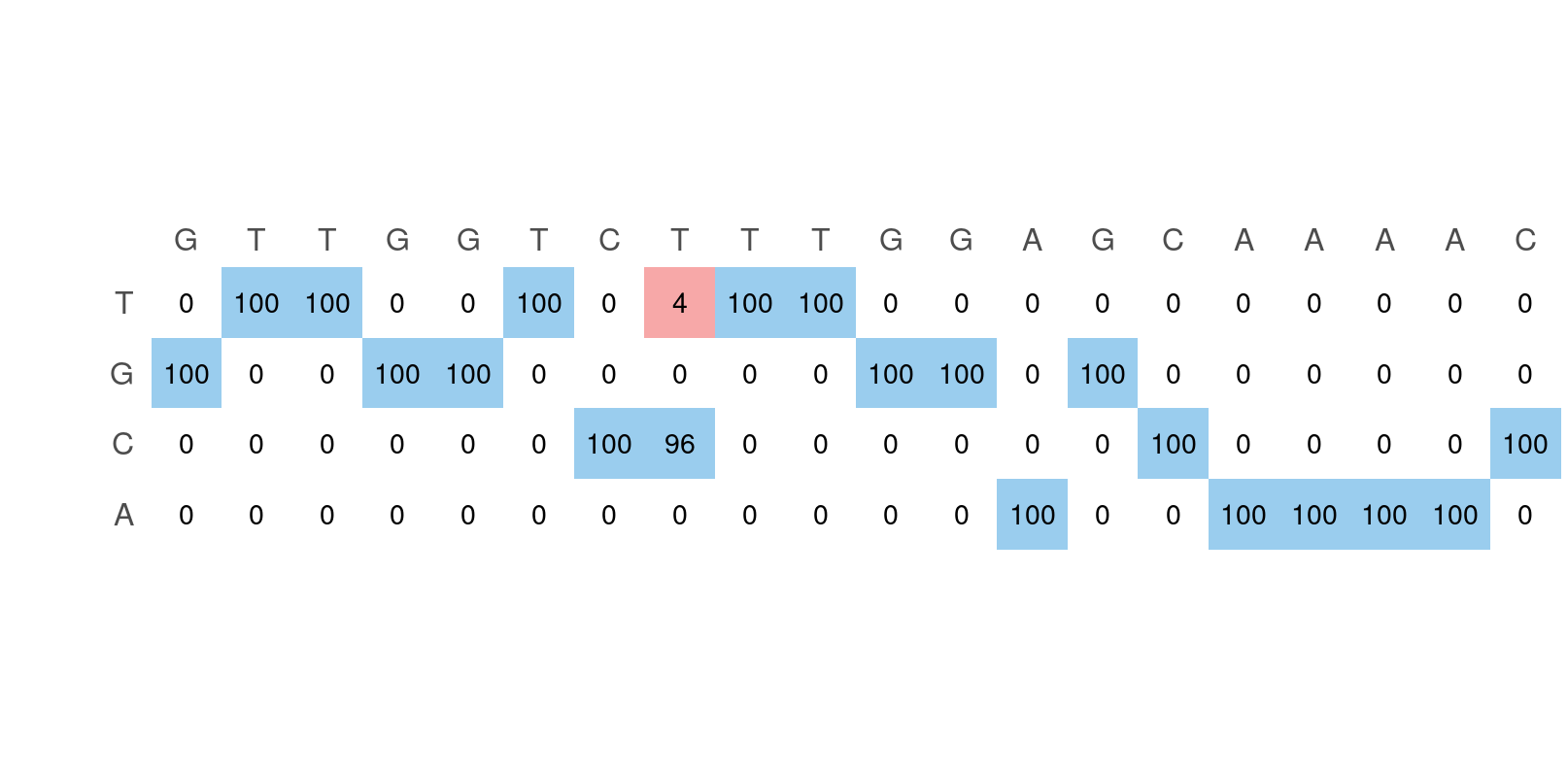
**

**R3**

**
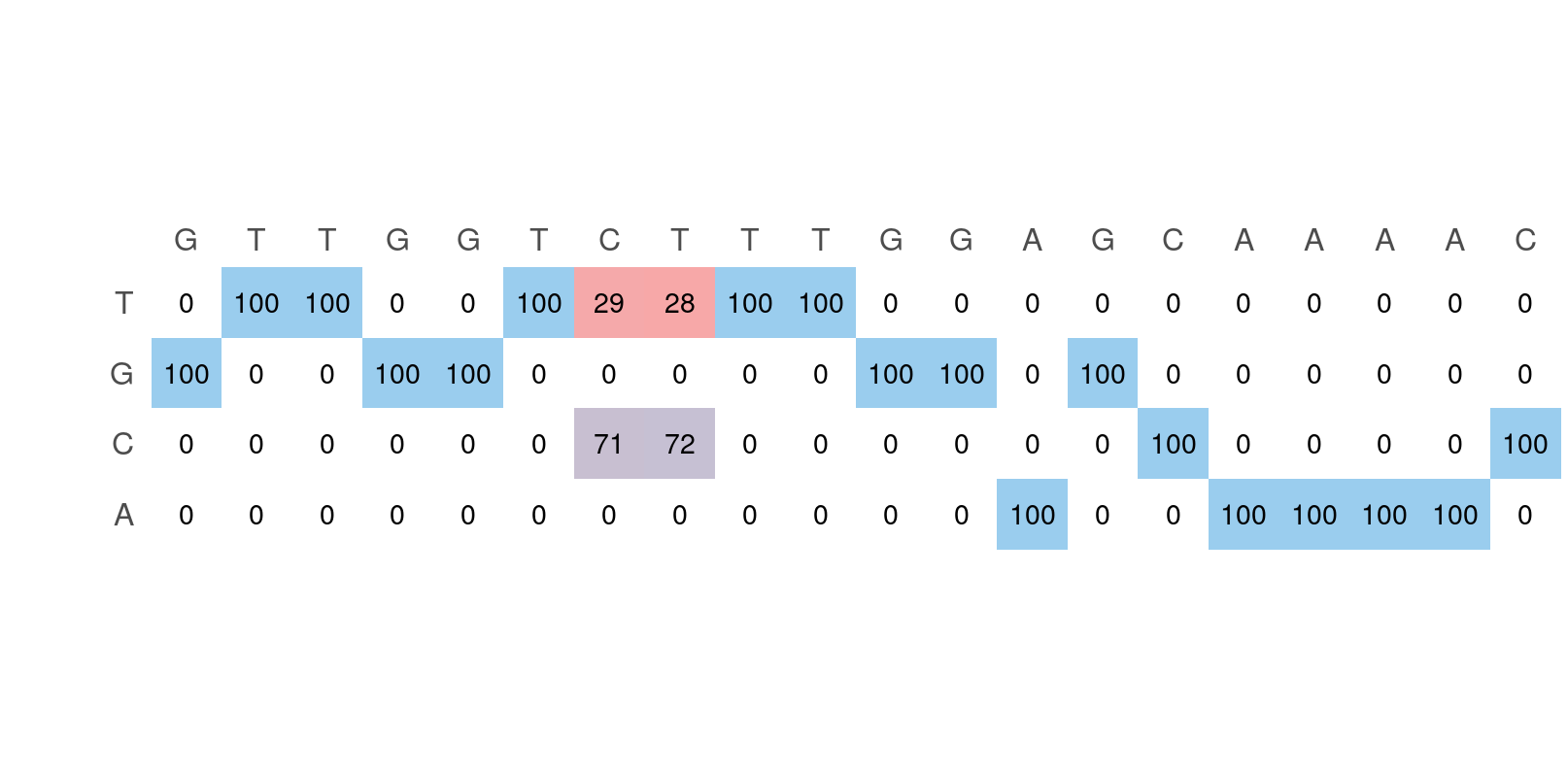
**

**R4**

**
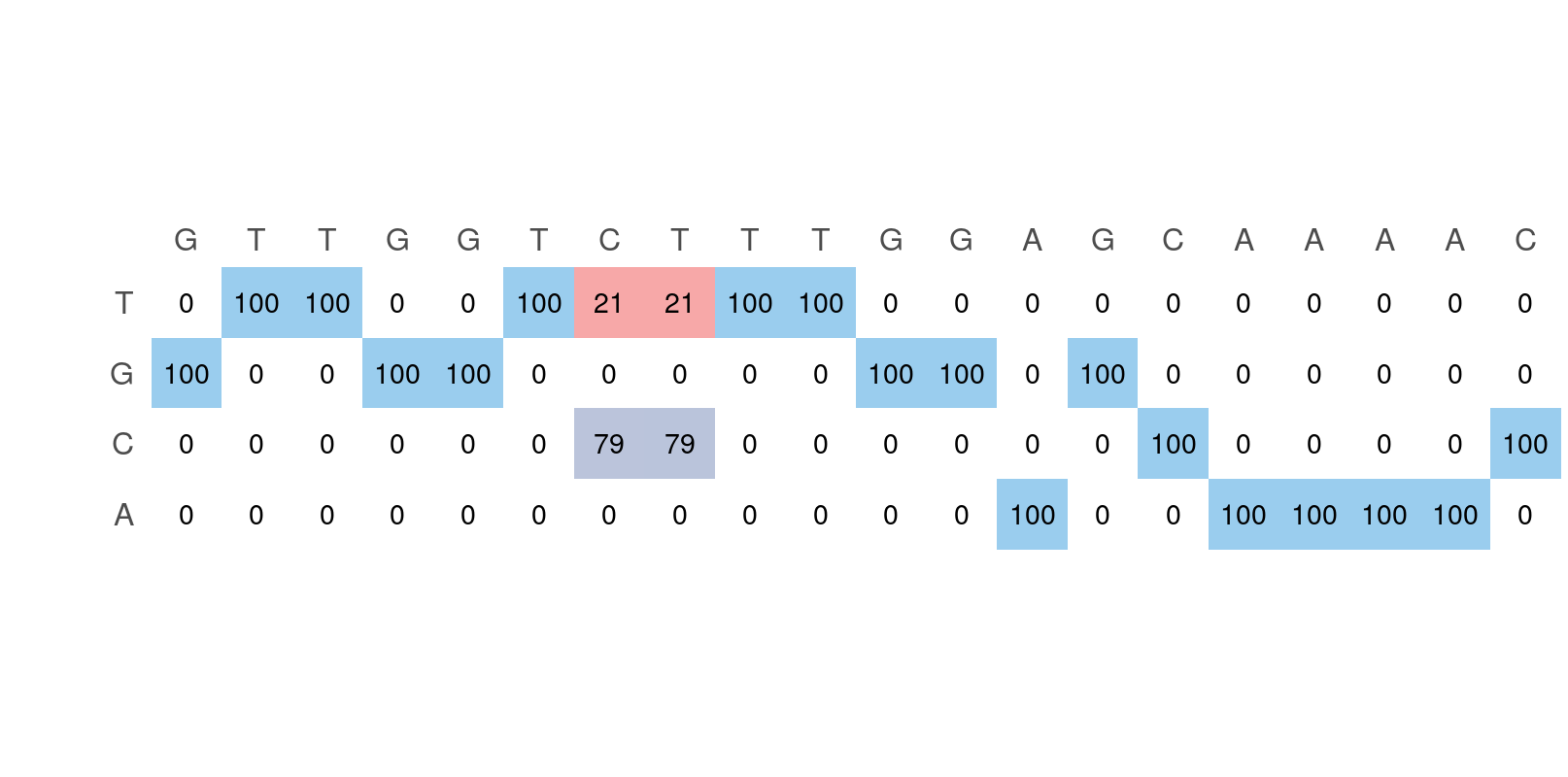
**

**C1**

**
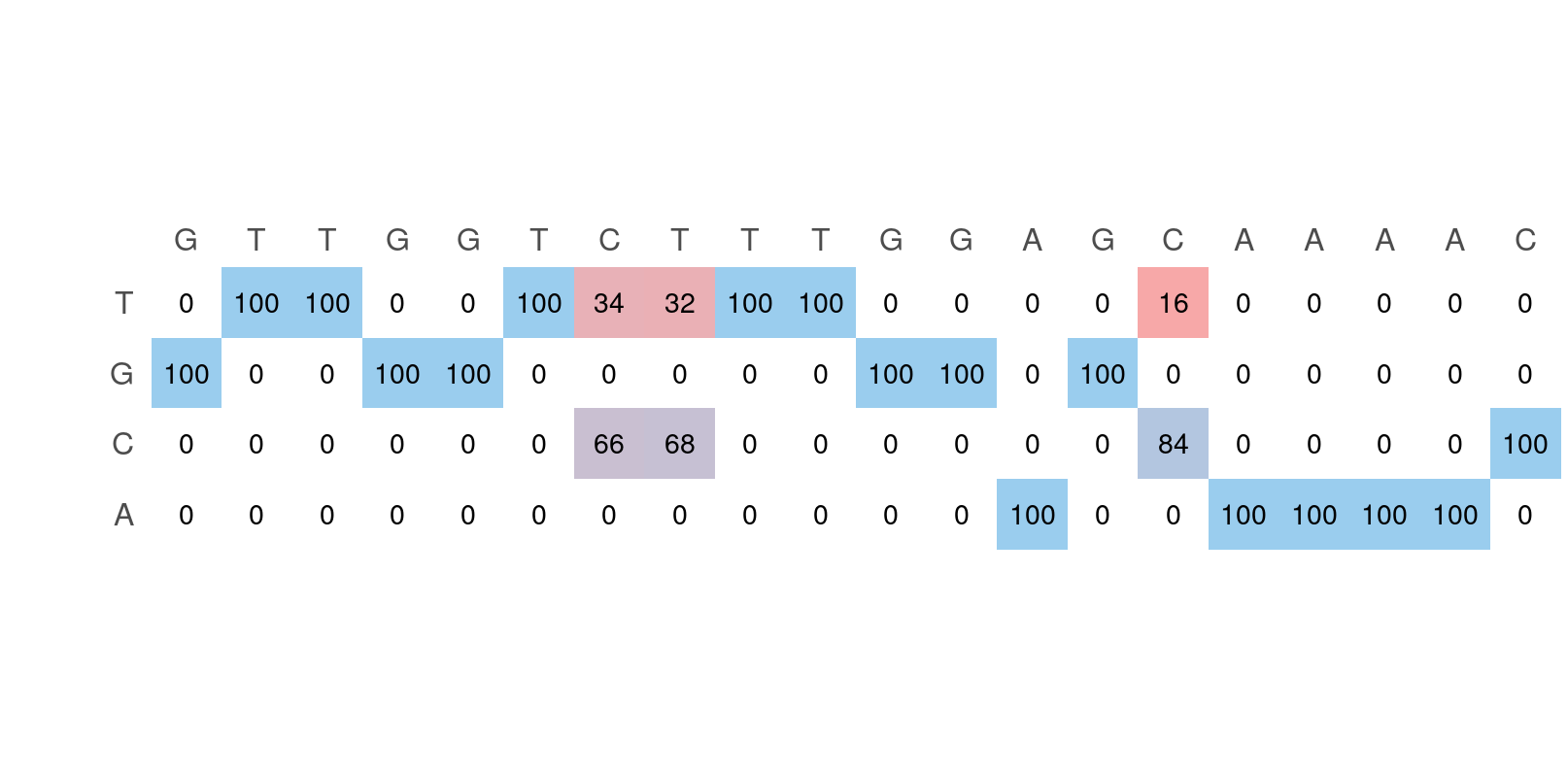
**

**C2**

**
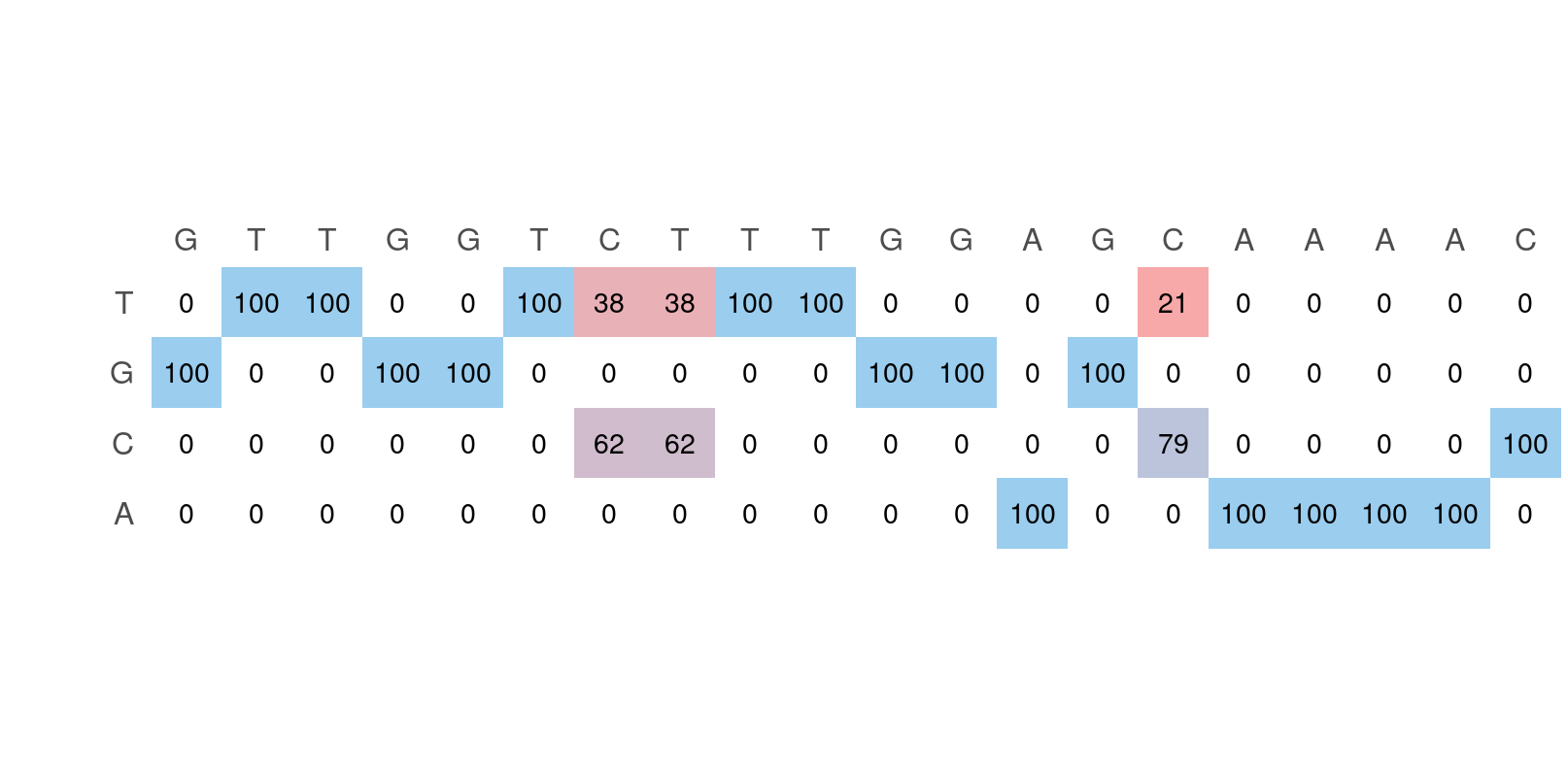
**

**C3**

**
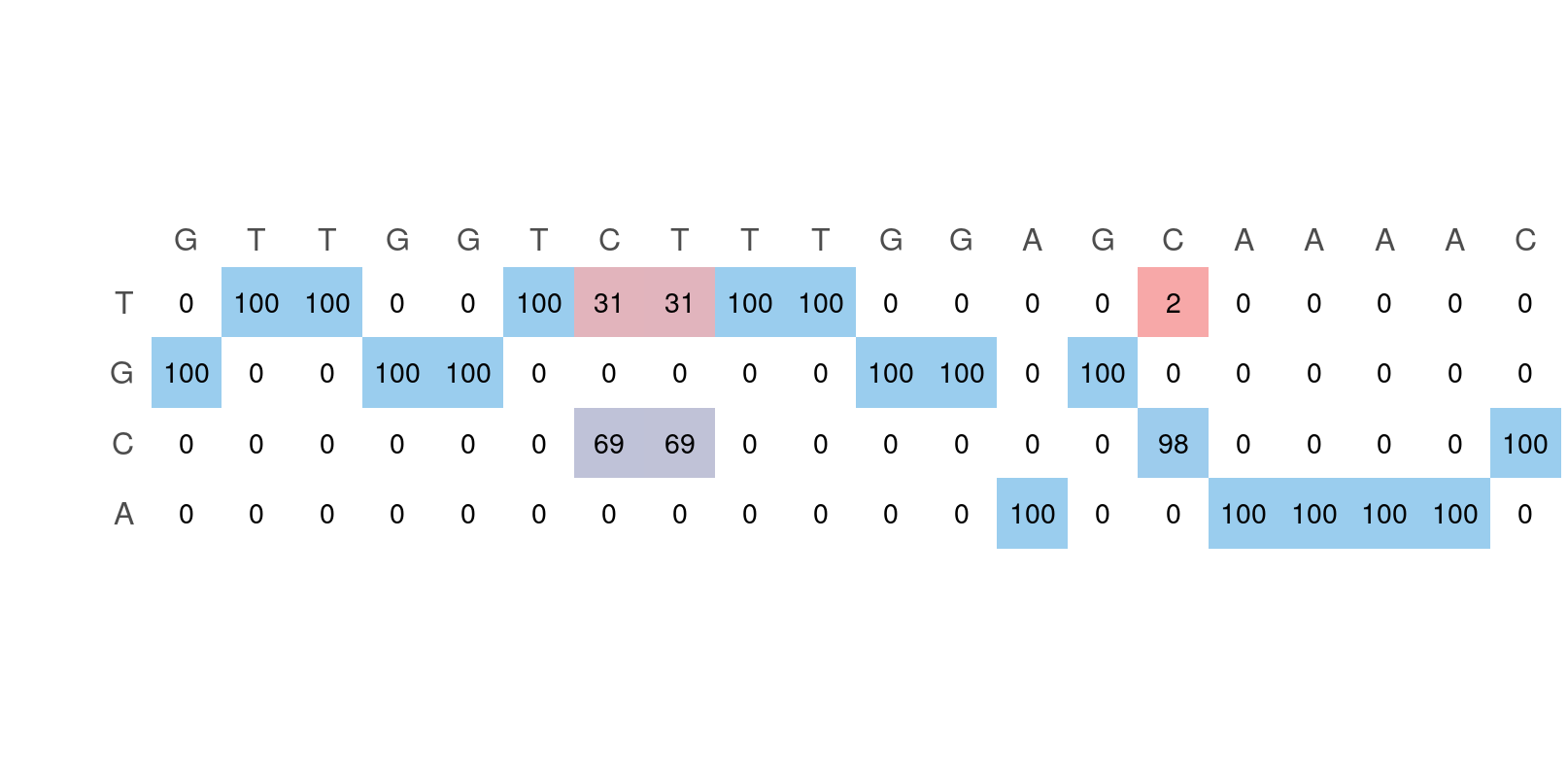
**

**C4**

**
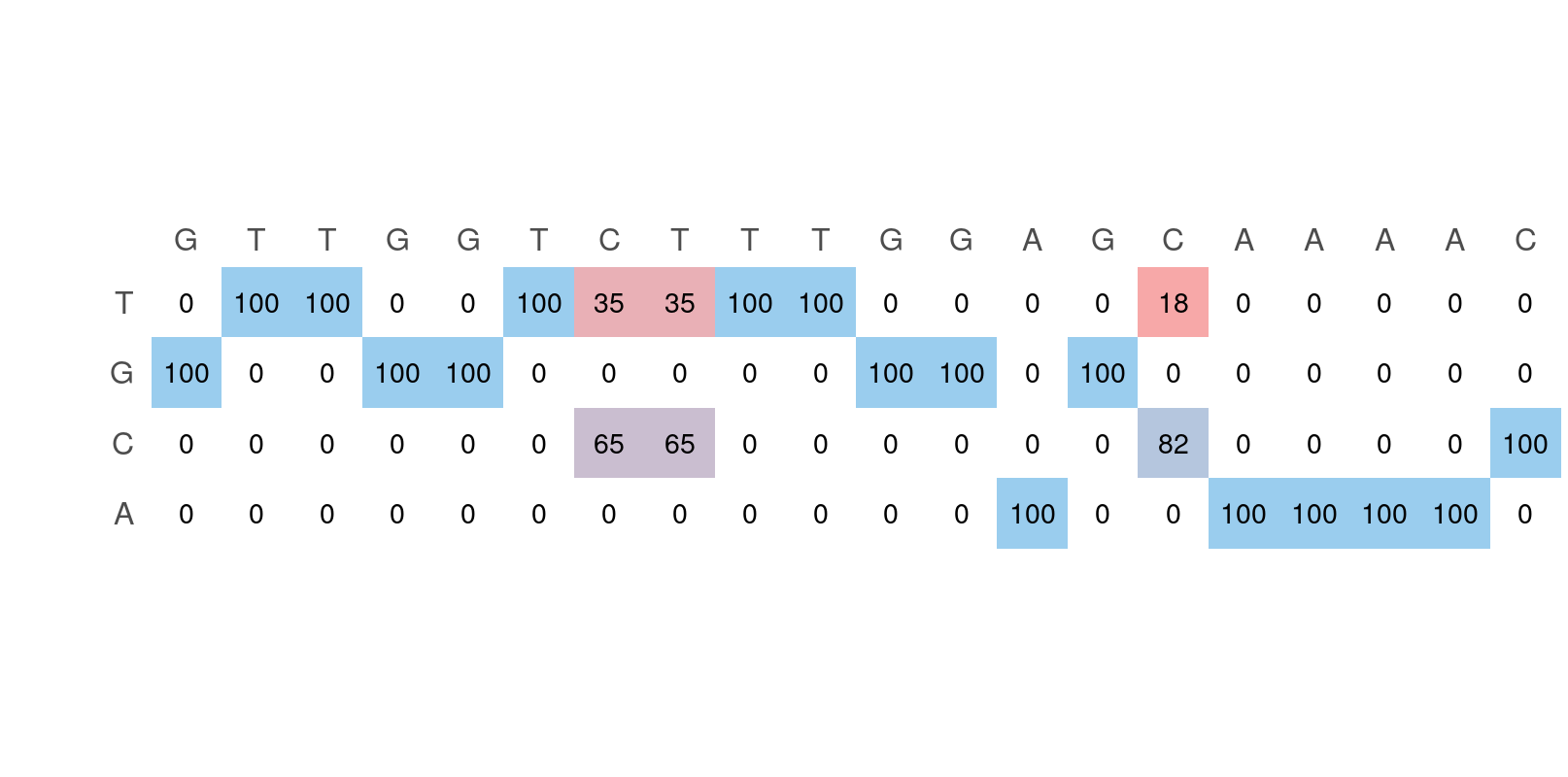
**

**GFP-1**

**
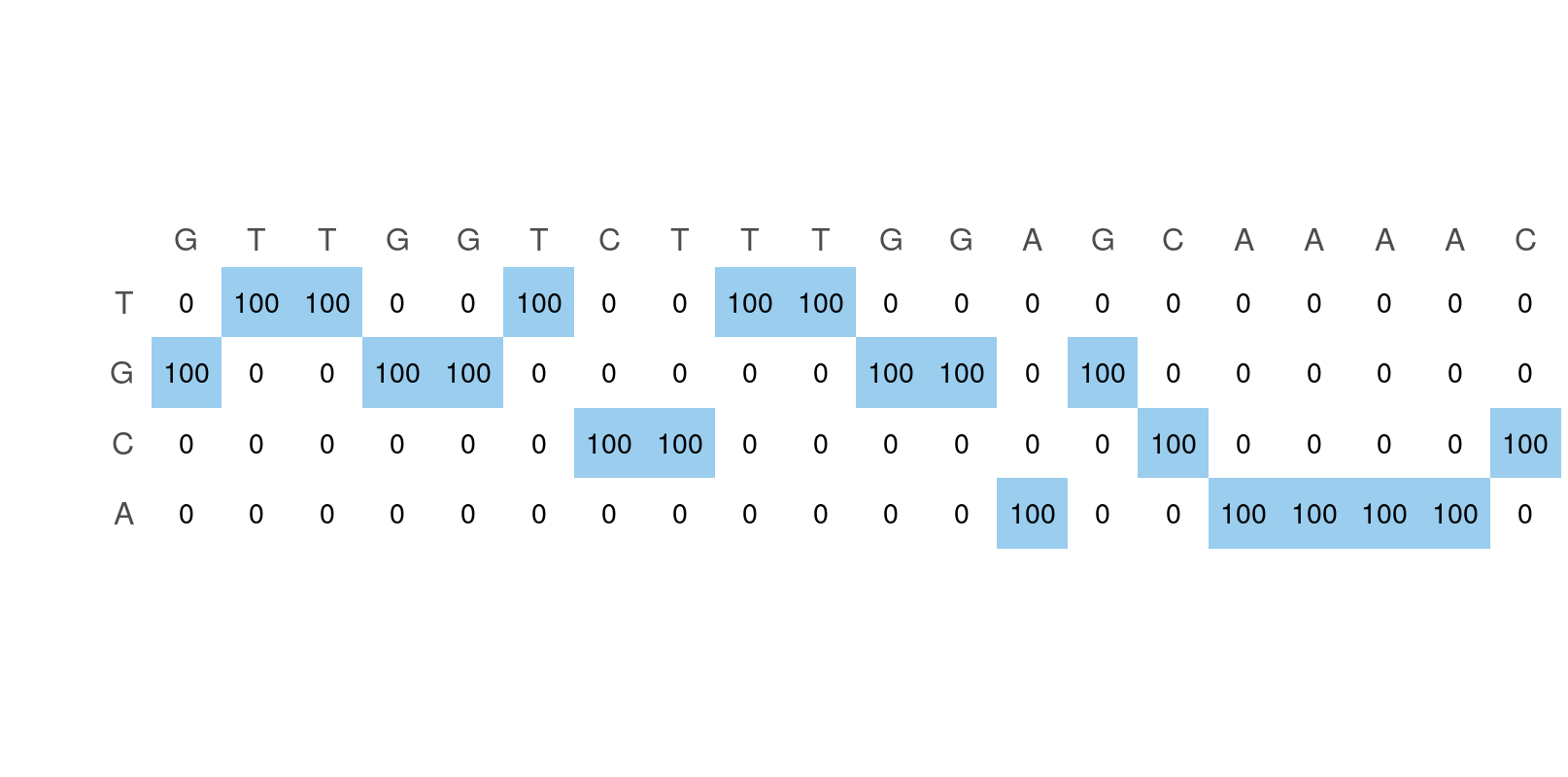
**

**GFP-2**

**
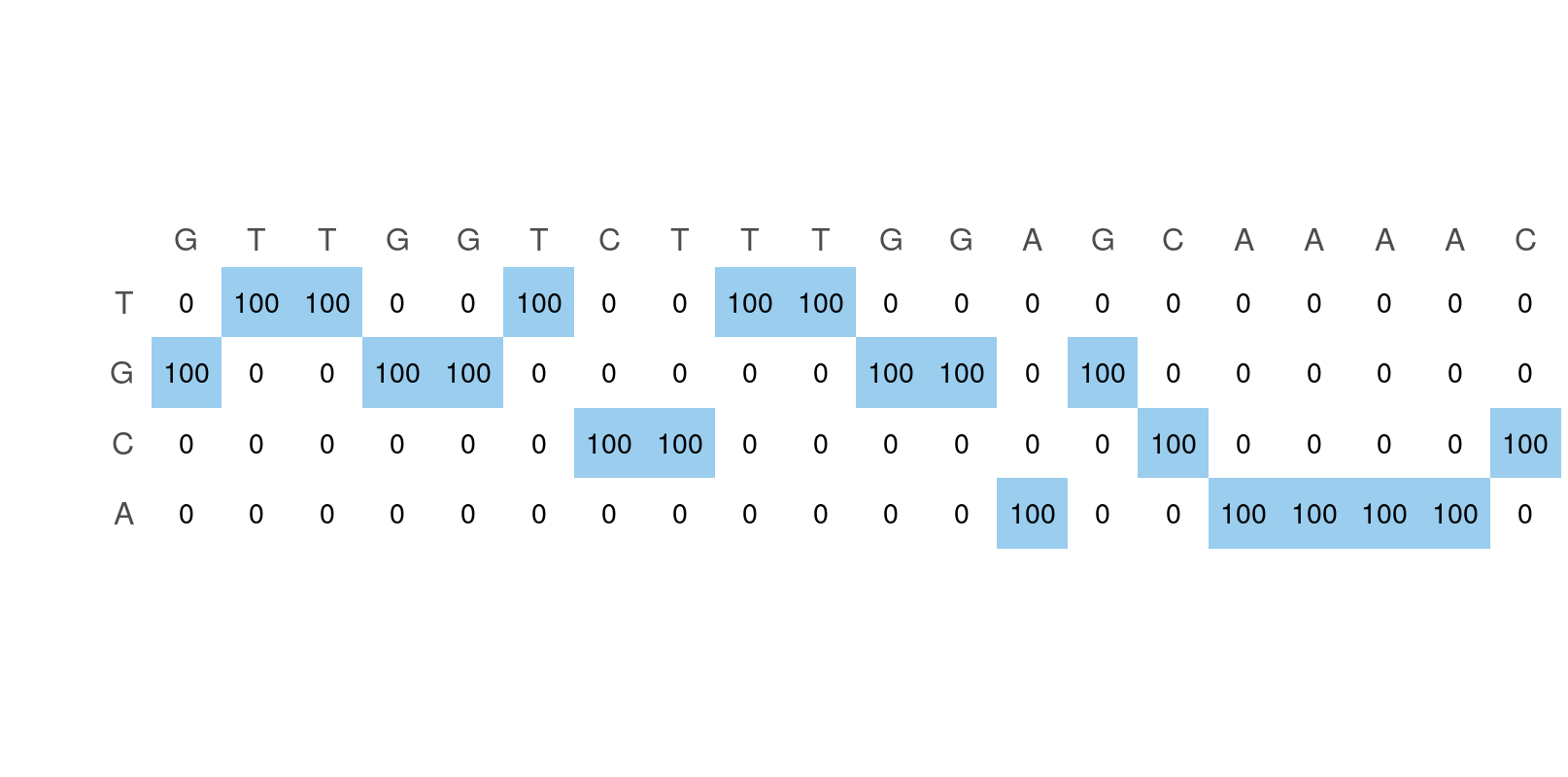
**

**nP16-1**

**
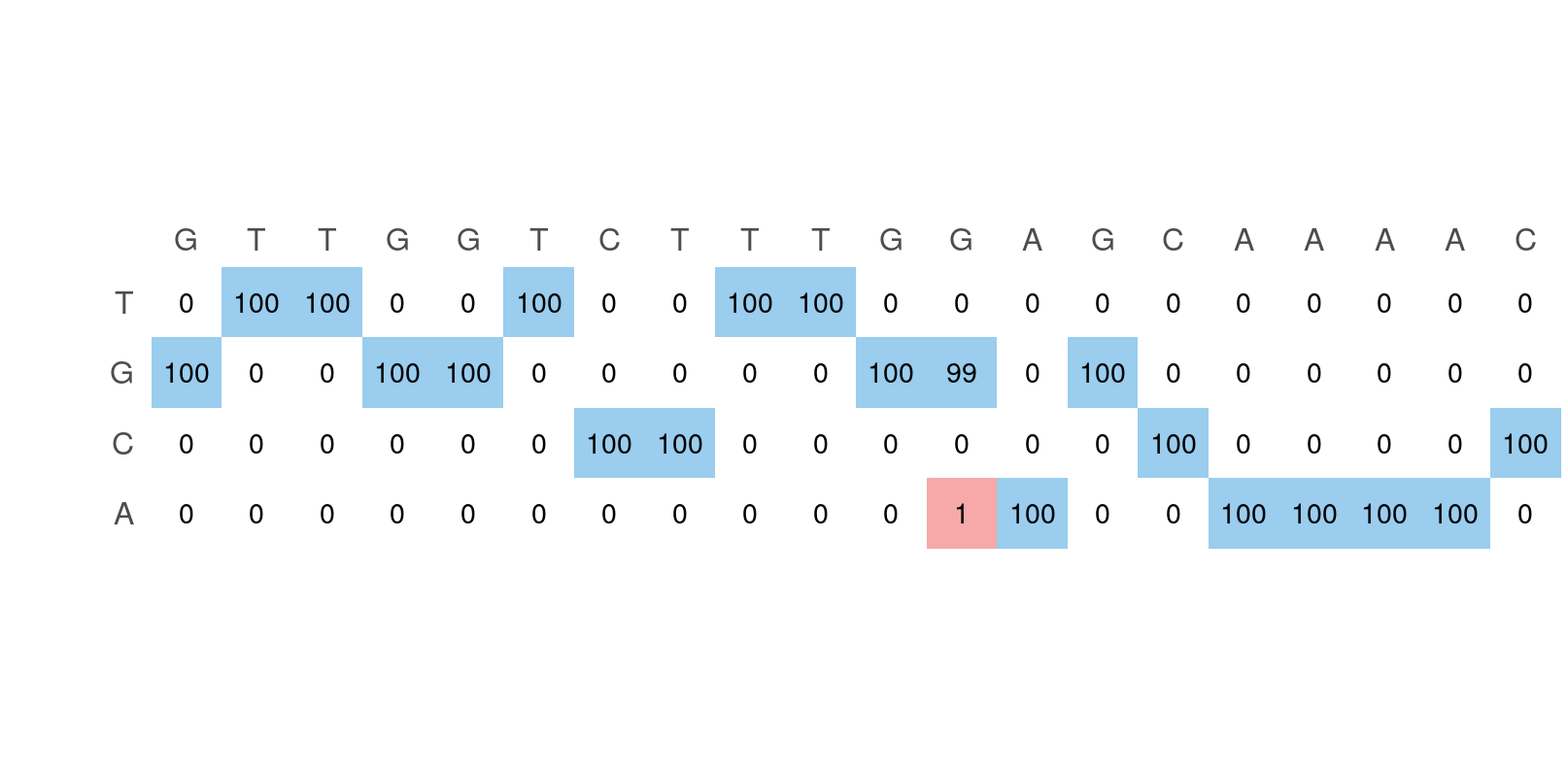
**

**nP16-2**

**
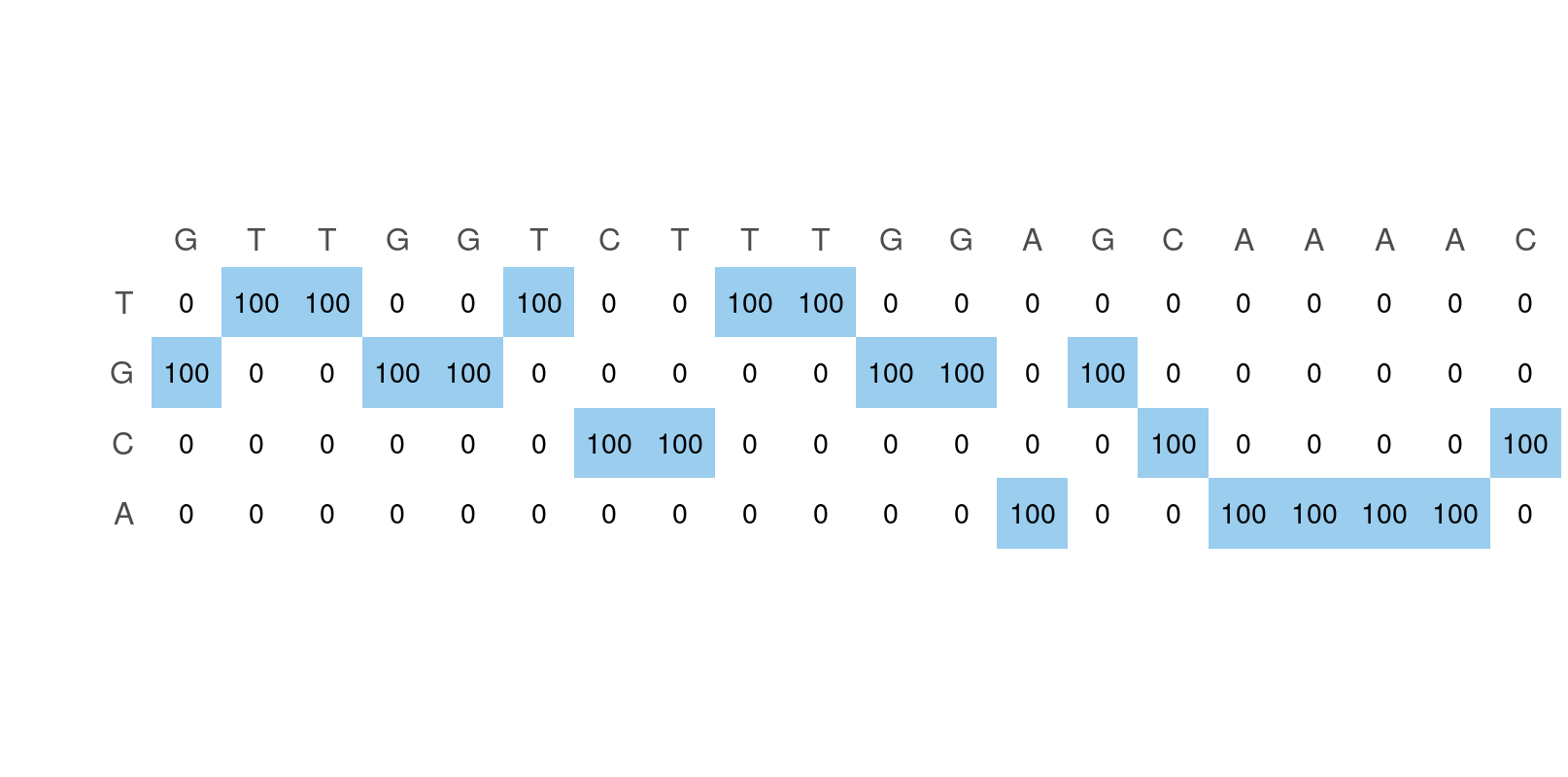
**

**Sequencing results of protoplast pool** (samples from Figure 2B)

| WT | Desiree wild type |
| --- | --- |
| A3A-4 | A3A replicate 4 |
| rA1-3 | rA1 replicate 3 |
| CDA1-2 | CDA1 replicate 2 |

**WT**

**
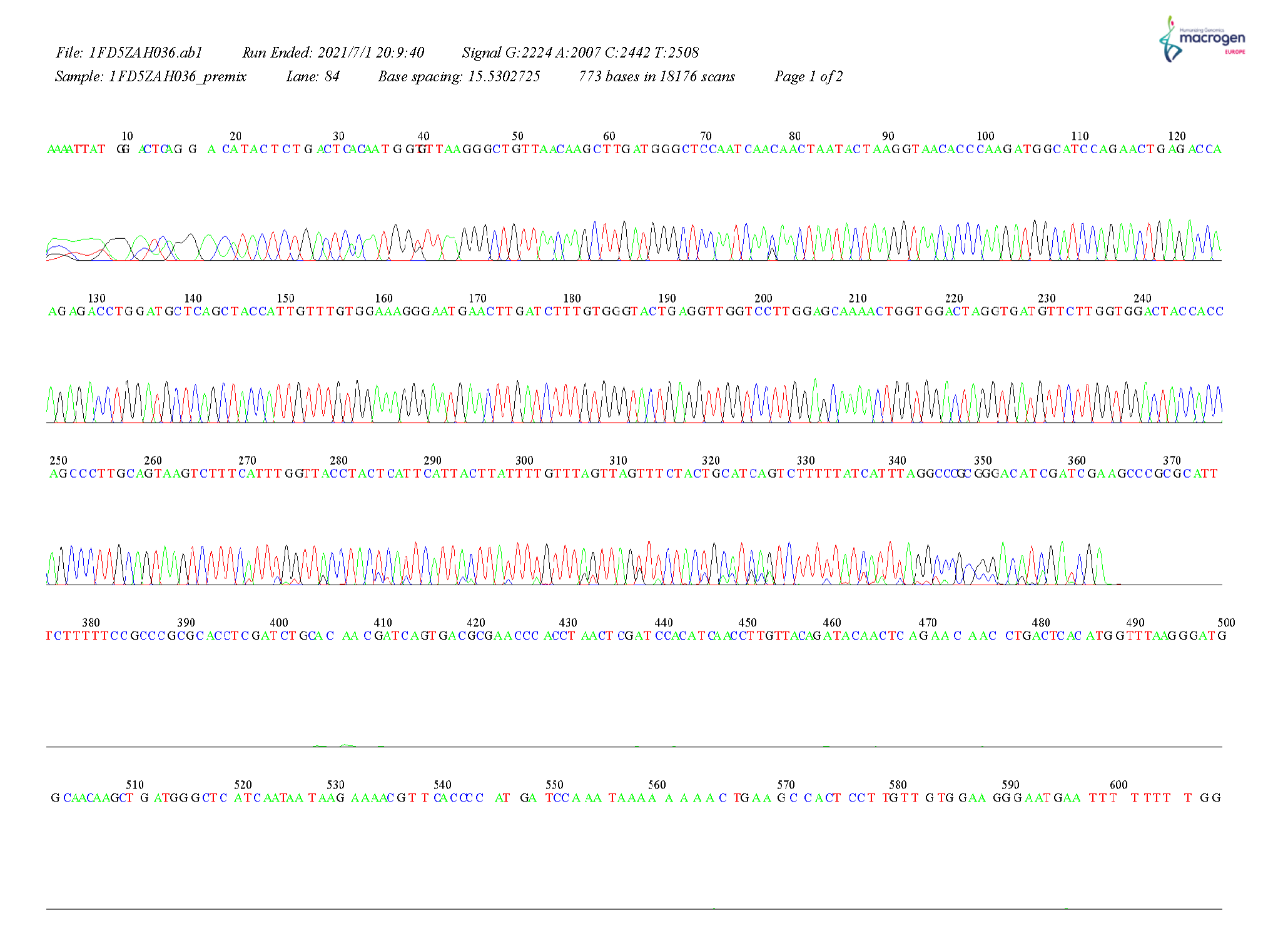
**

**A3A-4**

**
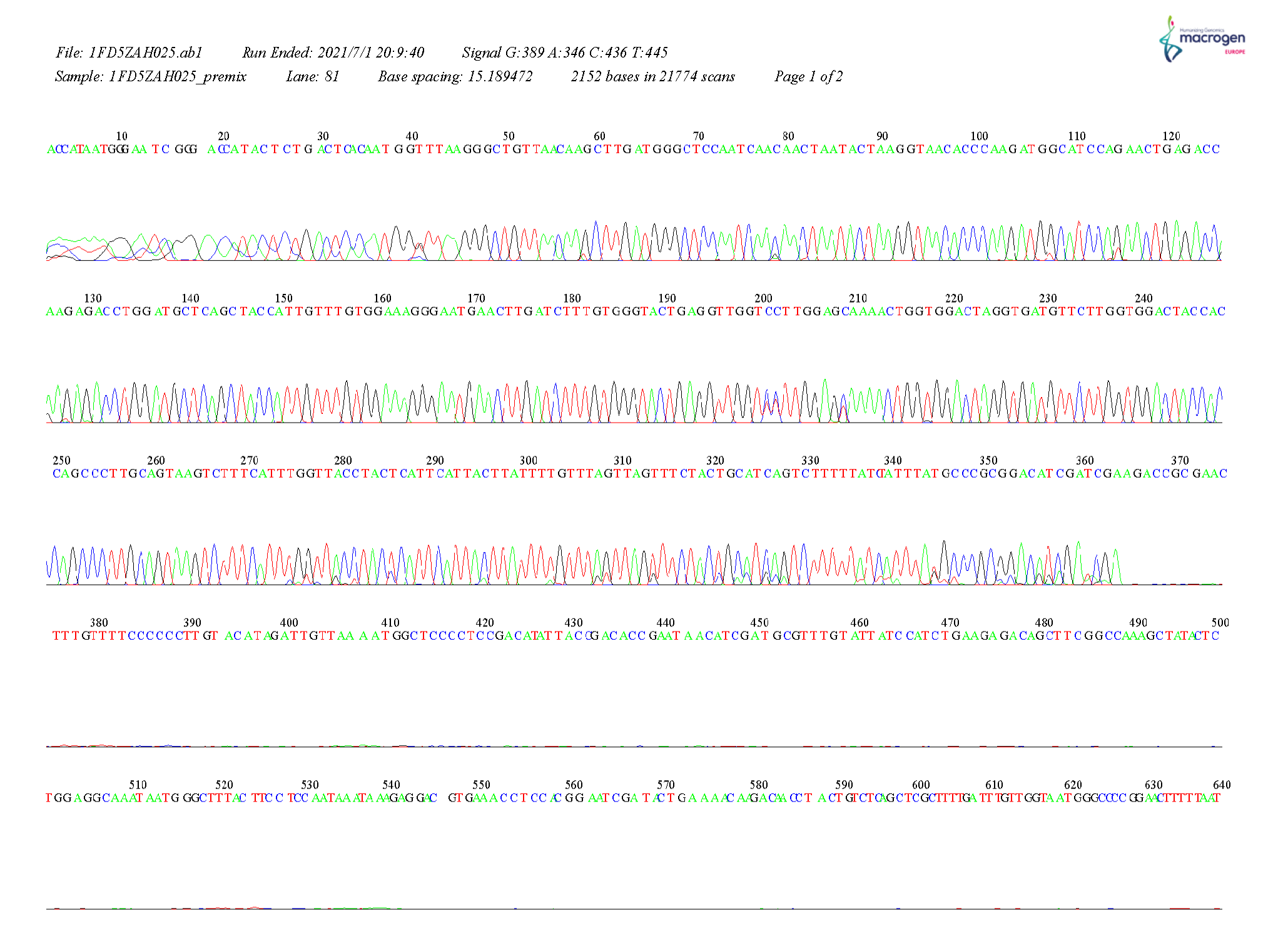
**

**rA1-3**

**
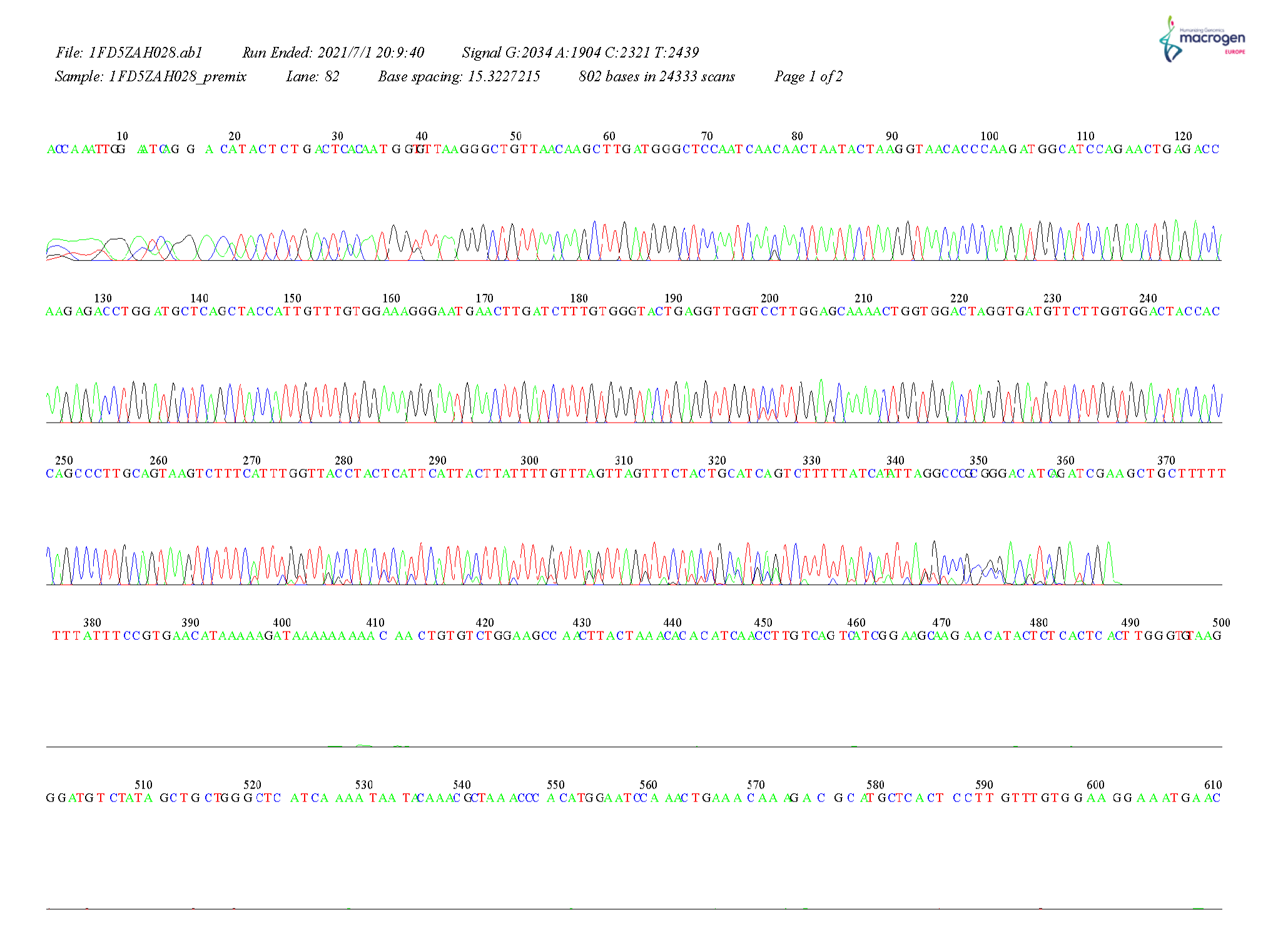
CDA1-2
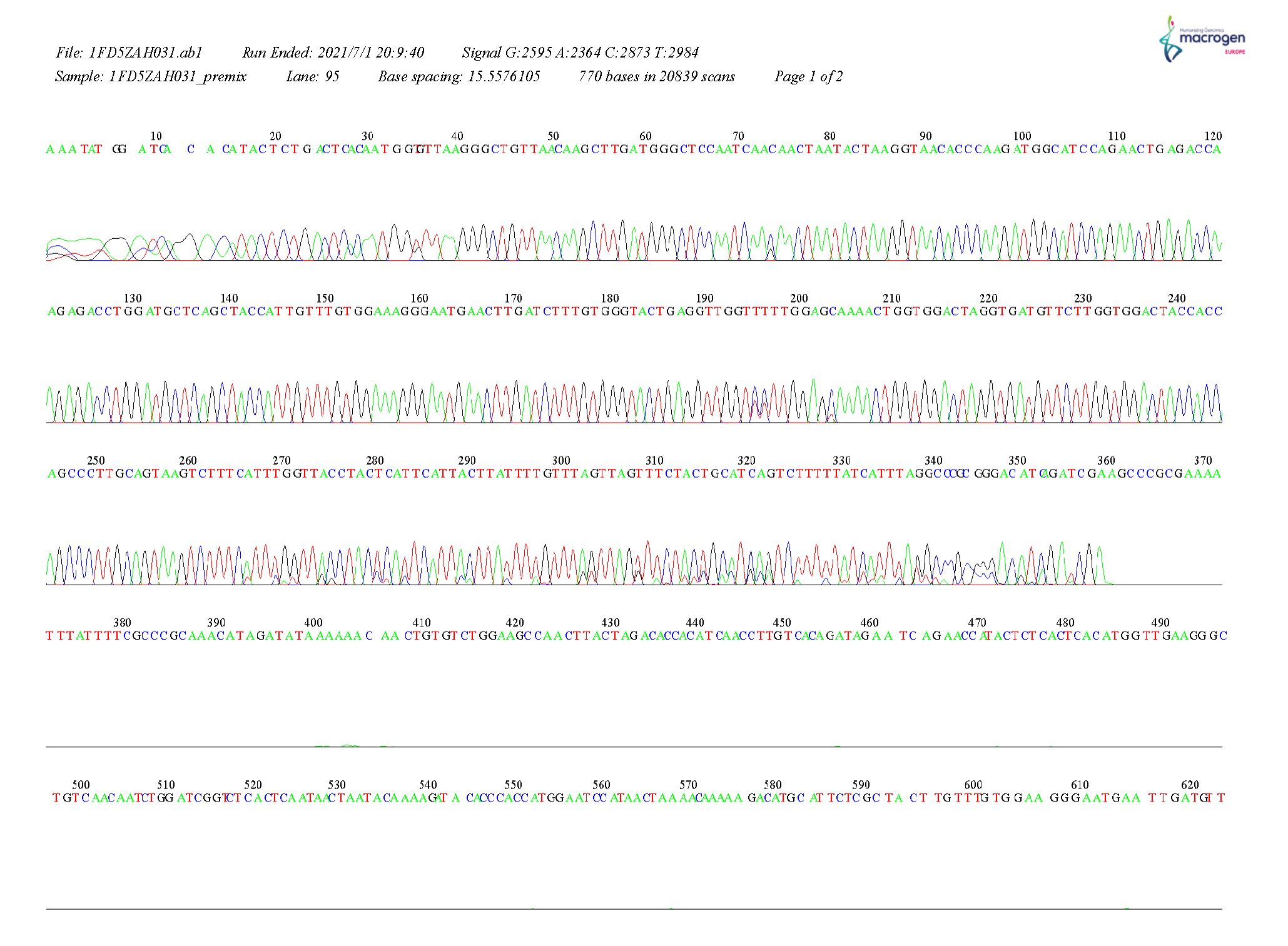
**

**Allele representation**

Direct sequencing using primer 589 on the protoplast pool transformed with A3A, rA1, and CDA1 (same samples as in Figure 2C) displaying the T/G (allele I + II + IV / allele III) and G/C polymorphisms (allele I + II / allele III + IV) shown in Figure 1, indicating a representation of all alleles. Four experimental replicates was aligned and assembled to WT Desiree sequence as reference. **
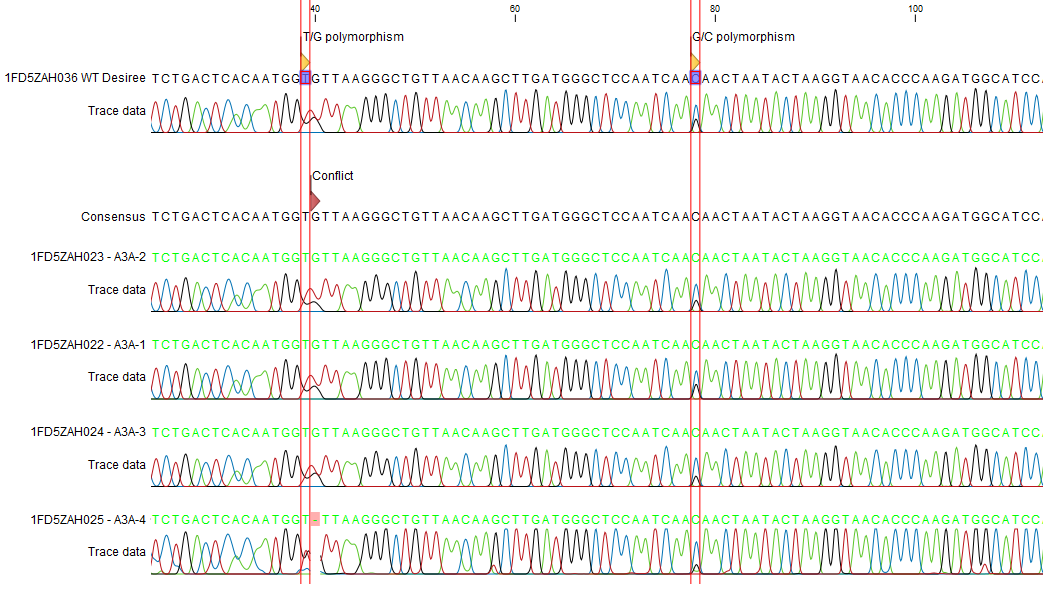
**
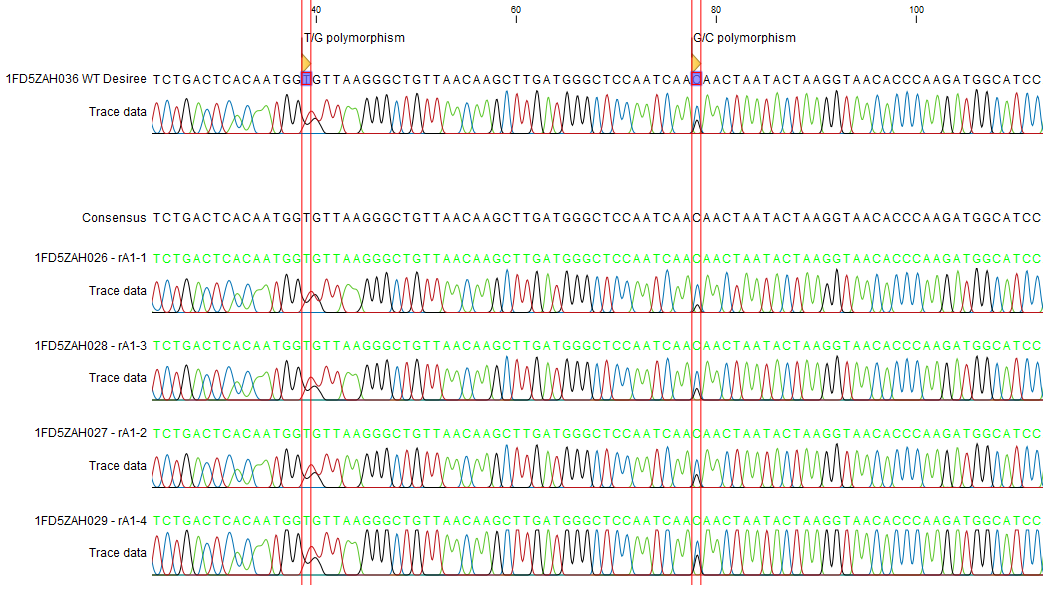

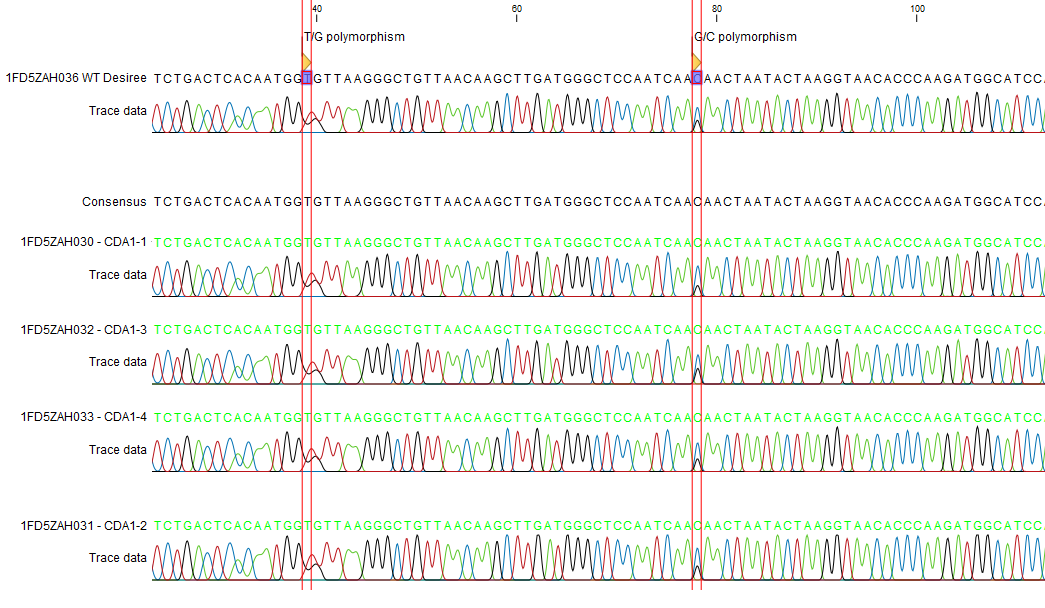

Supplement: Supplementary file 1 [file DataSheet1.docx]
